# Towards a Compositional Framework for Describing Human Phenotypes

**DOI:** 10.1002/advs.77730

**Published:** 2026-09-22

**Authors:** Wanting Hu, Yunchao Ling, Xinyue Hu, Wei Chen, Rong Zhou, Xinhao Zhuang, Yanfeng Jiang, Jialu Zhao, Xingdong Chen, Jingchun Luo, Li Jin, Guoqing Zhang

**Affiliations:** ^1^ Bio‐Med Big Data Center Shanghai Institute of Nutrition and Health University of the Chinese Academy of Sciences Chinese Academy of Sciences Shanghai China; ^2^ Human Phenome Institute Fudan University Shanghai China; ^3^ State Key Laboratory of Genetics and Development of Complex Phenotypes School of Public Health National Clinical Research Center for Aging and Medicine Huashan Hospital Fudan University Shanghai China; ^4^ Fudan University Taizhou Institute of Health Sciences Taizhou Jiangsu China; ^5^ State Key Laboratory of Genetic Engineering Collaborative Innovation Center for Genetics and Development School of Life Sciences Fudan University Shanghai China

**Keywords:** anomaly screening, data element standardization, data quality assessment, integrative research, international human phenome project, large language model agents, phenotype assembly

## Abstract

Deep phenotyping increasingly spans scales from macro to micro, yet heterogeneity in measurement and documentation still constrains standardization and limits comparability across cohorts. This study introduces a compositional framework that integrates the Phenotype Assembly Method (PhenoAM), Phenome Data Elements (PhenoDE), and a suite of large language model (LLM)‐based PhenoAgents to support systematic, interpretable, and scalable phenotype standardization with quality assessment. PhenoAM decomposes phenotype descriptions into features and qualifiers to enable component‐level mapping, while PhenoDE provides standardized representations of 58 371 phenotype data elements across 22 measurement platforms of the International Human Phenome Project (IHPP); pooled mapping coverage was 98.1% within the 18‐platform analysis set and 99.8% across all 22 platforms. Using this representation, analyses of illustrative phenotypes from IHPP, National Health and Nutrition Examination Survey (NHANES), and the UK Biobank show that differences in features or qualifiers can influence data distributions and analytic outcomes, underscoring the importance of explicit measurement context in cross‐cohort studies. PhenoAgents support semantic parsing, terminology candidate retrieval, and data‐quality review through curator‐assisted modules. PhenoDEs and their statistical distributions within IHPP are accessible on the PhenoDE Portal.

## Graphical abstract

The Phenotype Assembly Method (PhenoAM) decomposes phenotype variables into measurable Features and typed Qualifiers, enabling standardized, machine‐readable Phenome Data Elements (PhenoDEs) that preserve measurement context. Applied in the International Human Phenome Project (IHPP), the framework yields 58 371 PhenoDEs and supports component‐level terminology linkage and semantic comparison. Large language model (LLM)‐based PhenoAgents further assist semantic decomposition, terminology mapping, and data profiling.

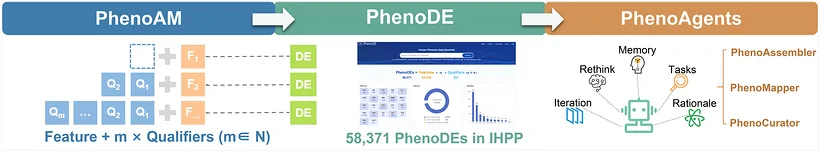

## 1 Introduction

The intricate mechanisms linking human genetics to disease phenotypes highlight the importance of deep and standardized phenotyping for advancing biomedical research [1, 2]. Advances in measurement and data acquisition have produced massive amounts of phenotype data (Figure S1), [3, 4] enabling population‐scale investigations in large longitudinal cohorts such as the UK Biobank [5] and the National Health and Nutrition Examination Survey (NHANES) [6]. Since 2018, the International Human Phenome Project (IHPP) has extended this effort by capturing phenotypes across more than twenty measurement platforms that span molecular omics, cellular profiling, imaging, and behavioral assessments [7, 8]. However, differences in measurement domains, evolving instruments, and protocol updates across platforms and cohort cycles introduce substantial heterogeneity, which constrains reproducibility, interoperability, and comparability in longitudinal cohort research.

Existing phenotype standards provide valuable building blocks for representing and exchanging phenotype information. The Human Phenotype Ontology (HPO) focuses on abnormal human phenotypes, while measurement context is not intrinsic to individual HPO terms [9]. Phenopackets provide a flexible machine‐readable schema for exchanging phenotypic and clinical information, although the completeness of measurement context depends on the populated record [10]. The National Institutes of Health (NIH) Common Data Elements (CDE) repository supports reusable data elements and forms for standardized data collection, while detailed measurement context may be specified separately [11]. Taken together, these approaches support complementary forms of standardization, but detailed measurement‐context semantics may remain embedded in annotations, supplied record content, or distributed fields rather than being represented as explicit and reusable semantic components. However, for accurate interpretation and comparison, cohort variables require information not only on what is measured but also on the context in which the measurement is performed, such as sample type, fasting status, or measurement modality. Such contextual factors can materially influence measurement results and reproducibility [12]. In practice, such contextual information may be embedded in variable names and definitions or distributed across associated metadata, and its representation can vary across studies. Consequently, apparently similar phenotype variables may differ in important measurement details, highlighting the need for explicit and fine‐grained phenotype descriptions.

Beyond semantic standardization, data quality is an equally critical challenge for cohort research [13]. Variations in study design, instrument performance, operator behavior, and participant compliance, as well as instrument drift, can introduce latent inconsistencies. The diversity of data modalities further complicates quality control, making systematic assessment both resource‐intensive and difficult to scale [14, 15]. A scalable and context‐aware approach to quality assessment is therefore needed to support reproducible and transparent cohort data management.

Here, we address these challenges with an integrated framework comprising the Phenotype Assembly Method (PhenoAM), standardized Phenome Data Elements (PhenoDE), and a family of large language model (LLM)‐based agents, collectively termed PhenoAgents. PhenoAM decomposes each phenotype into a core feature and several contextual qualifiers, yielding a structured and interpretable representation. On this foundation, PhenoDE systematizes metadata in the IHPP for 58 371 standardized phenotype data elements derived from 22 IHPP‐specific measurement platforms and organized into 14 controlled vocabularies. HPO, Phenopackets, NIH CDE, and PhenoAM/PhenoDE address complementary aspects of phenotype representation, with PhenoAM/PhenoDE focusing on compositional representation of cohort‐variable semantics through reusable feature‐qualifier components for cross‐cohort comparison (Note S1). Using this standardized structure, analyses of illustrative phenotypes from the IHPP, NHANES, and the UK Biobank show that differences in feature and qualifier specifications are associated with shifts in data distributions and analytical results, illustrating the importance of explicit measurement context. PhenoAgents then automate feature and qualifier extraction, terminology candidate retrieval, and interpretable data quality review, facilitating scalable and transparent cohort data management. Together, these components establish an integrated and compositional framework for phenotype standardization and data quality assessment, supporting more comparable and interpretable cohort analyses.

## 2 Results

### 2.1 A Unified Framework for Systematic Phenotype Standardization and Large Language Model‐Assisted Data Quality Assessment

**FIGURE 1:**
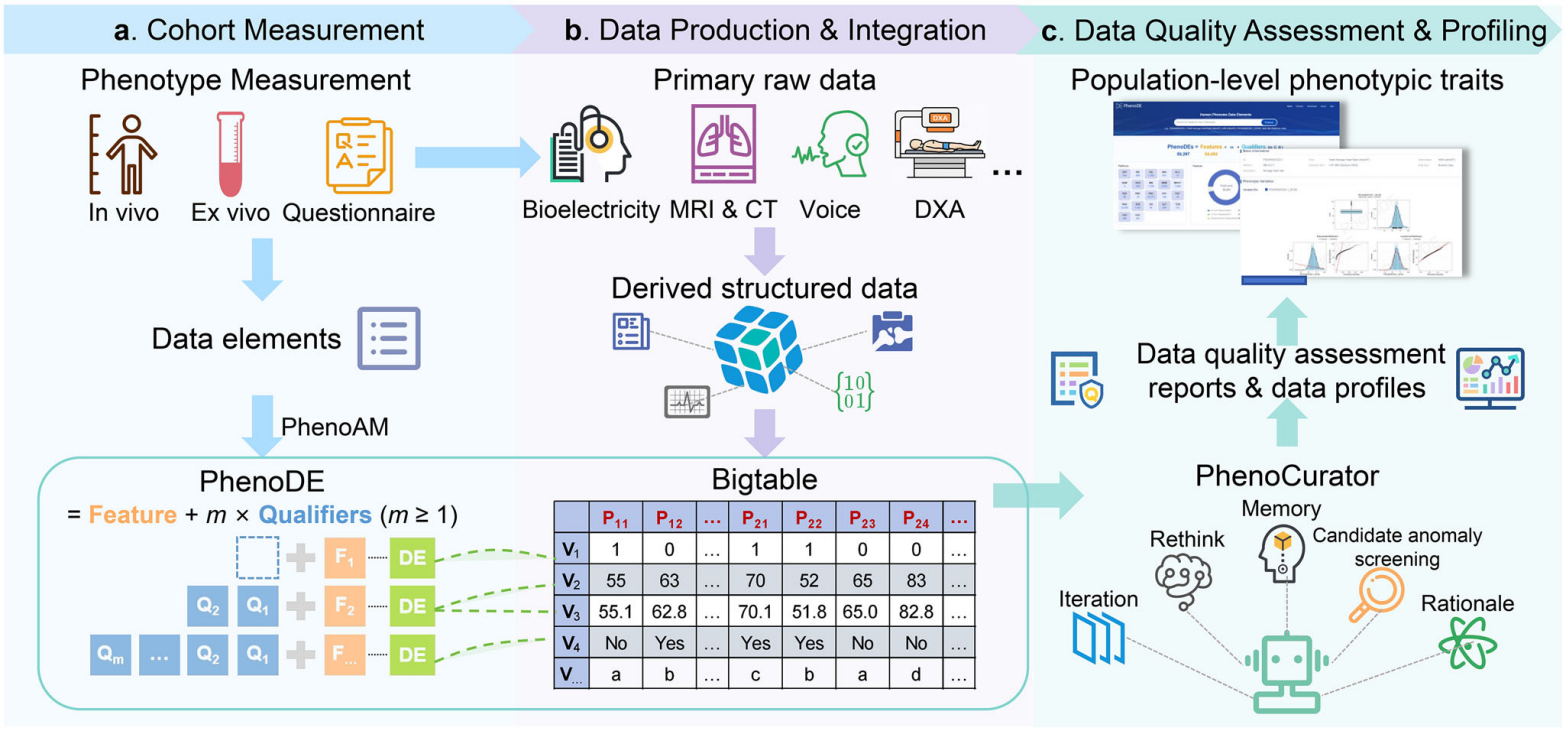
A unified framework for phenotype standardization and large language model (LLM)‐assisted data quality assessment. (a) Phenotypes from in vivo, ex vivo, and questionnaire modalities are decomposed by PhenoAM into a core feature and a set of qualifiers, which are organized into standardized PhenoDEs. PhenoAM, Phenotype Assembly Method; PhenoDE, Phenome Data Elements. (b) Heterogeneous data from multiple sources (Bioelectricity, magnetic resonance imaging (MRI), computed tomography (CT), voice, dual‐energy X‐ray absorptiometry (DXA), etc.) are transformed into structured matrices and merged into a Bigtable linked to participants, samples, and temporal information. Each real‐world variable, including repeated or condition‐specific measurements, is semantically mapped to its corresponding PhenoDE. (c) PhenoCurator, an LLM‐based agent, uses memory‐enhanced reasoning to iteratively assess the Bigtable and generate data quality assessment reports, data profiles, and summaries accessible via the PhenoDE Portal.

Human phenotype data are inherently multidimensional and heterogeneous, which creates persistent challenges for standardized representation, data integration, and quality evaluation. In most cohort projects, researchers primarily access final analytical datasets, while upstream steps such as measurement design, semantic normalization, and data validation remain largely invisible. To improve transparency and interpretability, we developed a unified framework that links cohort measurement, data integration, and data quality assessment (Figure 1). The framework comprises three main components: PhenoAM for semantic decomposition, PhenoDE for standardized data representation, and PhenoAgents, a family of LLM‐based agents including PhenoCurator for data quality assessment.

During the cohort measurement stage (Figure 1a), study‐specific data elements are defined by integrating measurement protocols and metadata schemas that specify the study design, measurement modality, and data attributes. To achieve fine‐grained description and semantic consistency, PhenoAM decomposes each phenotype into a feature that defines the measurable property and a set of qualifiers that describe contextual dimensions related to the measurement object, process, or result. This explicit, hierarchical representation captures details that are often omitted in traditional variable definitions and supports semantic comparability within and across cohorts. The resulting PhenoDEs form a standardized vocabulary that enables consistent interpretation of phenotype metadata.

In the data production and integration stage (Figure 1b), heterogeneous data from multiple measurement domains are transformed into structured phenotype matrices and linked to participant, sample, and temporal information to construct an integrated Bigtable. Real‐world cohort data frequently include repeated or longitudinal measurements. For example, body weight may be recorded multiple times using different instruments in different settings, and blood pressure is often measured repeatedly to ensure accuracy [16]. Each measurement is mapped to the PhenoDE corresponding to its semantic definition, allowing repeated measurements to be linked while preserving differences in instrument, setting, or other contextual qualifiers. This mapping consolidates fragmented datasets into a coherent and semantically aligned structure that supports both cross‐sectional and longitudinal analyses.

Reliable downstream analysis requires rigorous evaluation of data completeness, consistency, and plausibility. PhenoCurator performs automated candidate screening and interpretable assessments of the integrated Bigtable (Figure 1c). It classifies variable types, identifies candidate missing values and anomalous values, and flags potential inconsistencies based on both statistical patterns and semantic context. The resulting data quality assessment reports and data profiles summarize candidate quality‐control issues and distributional characteristics of each phenotype variable, providing a basis for population‐level analyses and cross‐cohort comparisons.

Overall, this framework offers a transparent, semantically standardized, and LLM‐assisted approach to cohort data management. By unifying standardized representation, structured data organization, and interpretable quality assessment, the framework enhances interoperability, reproducibility, and quality review in large and multi‐cohort studies.

### 2.2 Systematic Characterization of Phenotype Features and Qualifiers Through Phenotype Assembly Method and Phenome Data Elements

**FIGURE 2:**
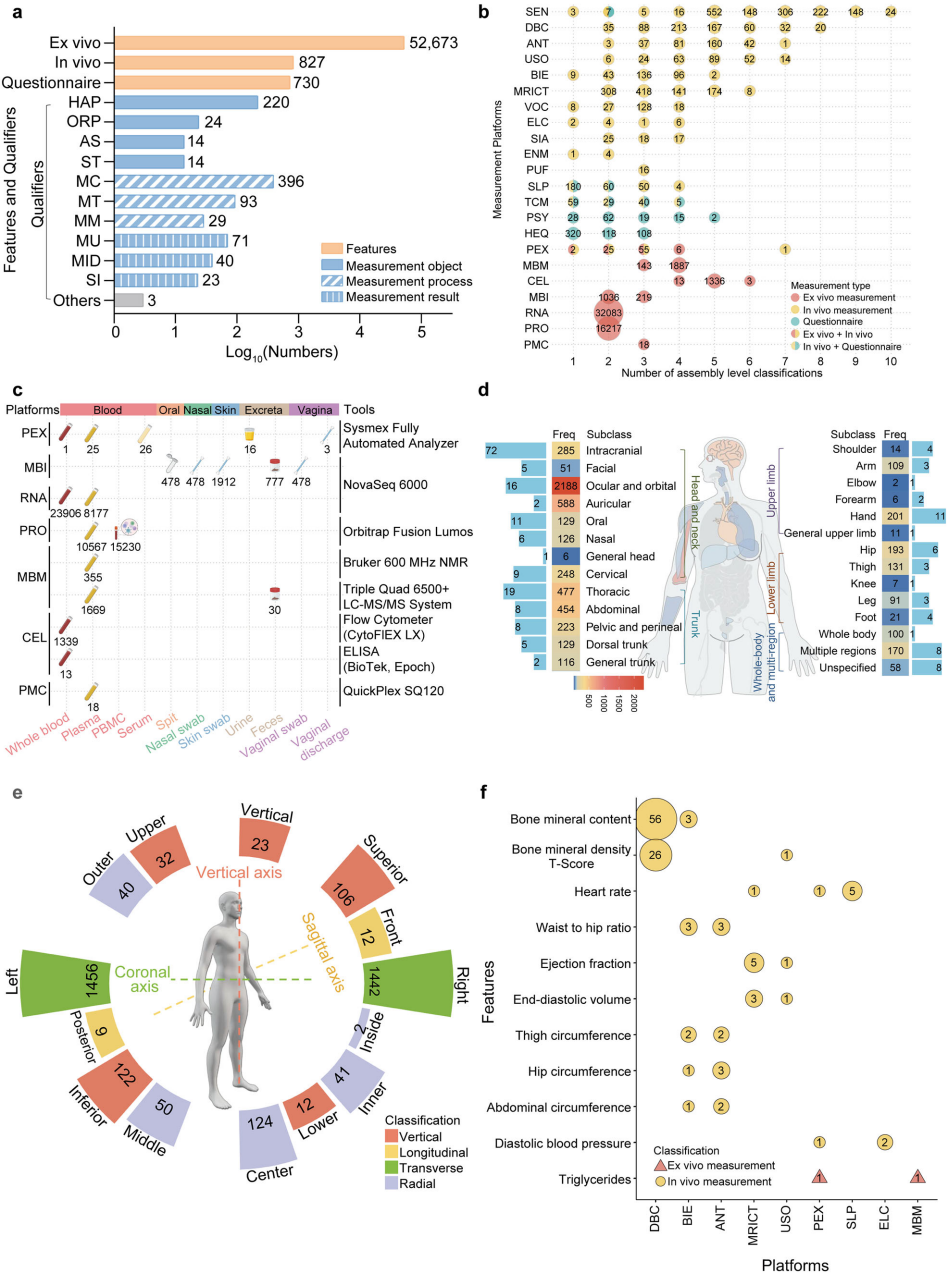
Systematic profiling of measurement objects, processes, and results with PhenoAM and PhenoDE. (a) Classification and counts of unique features and qualifiers in PhenoDE. Feature categories include ex vivo, in vivo, and questionnaire measurements. Qualifier categories include HAP (human anatomical parts), ORP (orientation and relative positions), AS (applicable subjects), ST (sample types), MC (measurement conditions), MT (measurement tools), MM (measurement methods), MU (measurement units), MID (measurement indicators), SI (statistical indicators), and Others. (b) Assembly depth of feature‐qualifier combinations across International Human Phenome Project (IHPP) phenotype measurement platforms. Assembly depth indicates the number of qualifiers combined with each feature, with bubble size representing the number of PhenoDEs and colors indicating measurement types. Row totals represent platform‐specific PhenoDEs. SEN, Sensory; DBC, DXA body composition; ANT, Anthropometry; USO, Ultrasonography; BIE, Bioelectricity; MRICT, MRI & CT; VOC, Vocal; ELC, Eligibility check; SIA, Skin and its appendages; ENM, Energy metabolism; PUF, Pulmonary function; SLP, Sleep; TCM, Traditional Chinese Medicine; PSY, Psychology; HEQ, Health questionnaire; PEX, General physical examination with blood laboratory tests; MBM, Metabolome; CEL, Cell; MBI, Microbiome; RNA, Total RNA and small RNA; PRO, Proteome; PMC, Plasma metabolic factors and cytokines. (c) Profiling of sample types and measurement tools for ex vivo phenotypes. Eight measurement tools were used to assess 11 sample types from 6 sources to characterize ex vivo phenotypes across 7 IHPP platforms. The skin swab category includes scalp, forehead, back, and ventral forearm. (d) Anatomical variability in detectable phenotypes. The 220 unique HAP qualifiers were distributed across 27 body region subclasses within five descriptive groups, with 6134 total occurrences. Colored cells indicate the frequency of HAP qualifier occurrences, whereas blue labels indicate the number of unique HAP qualifiers in each subclass. The grouping is used only for descriptive visualization and does not constitute a formal anatomical hierarchy. Detailed classification procedures are provided in Note S2. The central human heatmap used HAP‐related data, visualized via the MOAHIT website (https://smuonco.shinyapps.io/MOAHIT). (e) Orientation of human axes in in vivo phenotypes. In vivo phenotype orientations mainly include vertical, longitudinal (sagittal), transverse (coronal), and radial orientations. The 3D human figure was visualized using the Meshcapade editor (https://me.meshcapade.com/editor). (f) Feature distinctions across application scenarios. Variation in measurement objectives and processes causes the same features to describe analogous phenotypes across applications and platforms.

To achieve a standardized and fine‐grained representation of human phenotypes, we implemented PhenoAM and PhenoDE within the IHPP. In PhenoAM, phenotype features are classified by measurement type into ex vivo, in vivo, and questionnaire categories, while qualifiers are organized into eleven categories that describe the measurement object, process, and result (Figure 2a and Methods). This hierarchical system provides a precise and scalable foundation for representing phenotypes across biological levels, from molecular to organismal scales.

To quantify the structural complexity of phenotype descriptions, we defined assembly depth as the number of qualifier categories required to describe a given phenotype. Platforms such as Sensory and DXA body composition show the greatest depth, frequently involving seven or more qualifier categories (Figure 2b). For instance, “Optic disc layer thickness” measured on the Sensory platform has an assembly depth of ten and is recorded in the PhenoDE Portal (e.g., PDE057024733.1). Greater assembly depth indicates a more finely resolved phenotype, as additional qualifiers encode increasingly detailed measurement attributes and capture the higher resolution required in deep phenotyping. This multidimensional representation enables comprehensive characterization of complex phenotypes and supports systematic design of measurement items through scalable assemblies of features and qualifiers.

Ex vivo phenotypes are particularly sensitive to sample types and measurement technologies. Within the PhenoDE system, these phenotypes are captured across eleven sample types and eight related measurement instruments, explicitly linked through the sample types (ST) and measurement tools (MT) qualifiers (Figure 2c). Plasma samples alone provide more than 20 000 measurable molecular phenotypes, including metabolites, proteins, and RNAs, while the Microbiome platform involves five distinct sample types. By linking qualifiers to specific measurement contexts, this framework enables quantitative assessment of phenotype detectability and measurement diversity across platforms.

In vivo phenotypes are highly dependent on the anatomical region studied. To describe localization, we standardized these measurements using two qualifiers: human anatomical parts (HAP) and orientation and relative positions (ORP) qualifiers, which specify the targeted region and measurement direction, respectively. The 220 unique HAP qualifiers occurred a total of 6134 times and were summarized by body region into five descriptive groups and 27 subclasses (Figure 2d), whereas ORP qualifiers were mainly categorized into four orientation groups: vertical, longitudinal (sagittal), transverse (coronal), and radial orientations (Figure 2e). Together, these qualifiers facilitate consistent identification of anatomical regions across studies and platforms, and support phenotype‐wide association studies (PWAS) in domains such as sleep, bioelectricity, and imaging. For example, the PhenoDEs include 285 brain‐related phenotypes associated with 72 qualifiers, including “Brain” and 71 specific brain‐related qualifiers. More refined qualifiers are required for precise anatomical description in measurement‐intensive fields, such as Anthropometry, Ultrasonography, and Sensory assessment. In the Sensory platform, the qualifier “Center” denotes the macula as a central point, whereas in Ultrasonography, the qualifier “Middle” specifies the mid‐segment of the carotid artery within a defined region. The ORP qualifier is essential for direction‐sensitive phenotypes. Measurements of body composition and strength symmetry, obtained from circumferences and resistances on opposite sides of the body, can reveal biological balance and training effects [17]. Prior studies report that kettlebell athletes show enhanced upper body strength symmetry, that increased breast asymmetry in women is associated with lower salivary immunoglobulin A levels [18], and that hemispheric asymmetry in the brain relates to language, spatial orientation, communication, and fear processing [19, 20]. In brain research, applying ORP in magnetic resonance imaging (MRI) and related modalities is therefore crucial for linking regional structure and function to cognitive and behavioral phenotypes [19, 20].

At the measurement process level, we examined dominant features within individual platforms and shared features across multiple platforms to evaluate measurement specialization and generalization. We quantified feature frequency on each platform and the proportion of PhenoDEs contributed by each feature to identify platform‐specific dominance (Figure S2a). Platforms with dominant features often adapted their measurement procedures to capture multidimensional phenotypes. In particular, “Optic disc layer thickness” is represented by 590 PhenoDEs that combine qualifiers such as ORP, measurement conditions (MC), and measurement methods (MM). Detailed retinal measurements help reveal histological changes in myopic populations and support early detection of pathological alterations [21]. Reductions in macular ganglion cell‐inner plexiform layer (mGCIPLT) thickness and the macular retinal nerve fiber layer thickness can indicate retinal neurodegeneration, highlighting the association between blood pressure, arterial stiffness, and retinal health [22]. In contrast, some non‐dominant features are measured across multiple platforms for distinct purposes (Figure 2f). “Heart rate”, for instance, is recorded on the General Physical Examination with Blood Laboratory Tests platform as a daily vital sign described by HAP: heart and measurement units (MU): bpm, while on the Sleep platform, it is monitored across sleep stages as a key parameter for sleep disorder diagnosis and sleep/wake cycle analysis [23]. On the MRI & CT platform, average heart rate derived from cardiovascular magnetic resonance is used to assess cardiac function and overall health [24]. To capture these diverse contexts, heart rate must be paired with additional qualifiers, such as MT and MC, forming standardized PhenoDEs tailored to each platform.

At the measurement result level, derived or computed phenotypes require explicit documentation of analytical parameters, which can complicate method reporting. PhenoAM addresses this challenge by introducing the statistical indicators (SI) qualifier in PhenoDEs (Figure S2b). On the Vocal platform, SI records indicators such as the 0th coefficient of Mel‐frequency cepstral coefficients, refining the definition of acoustic phenotypes [25, 26]. In the Cell platform, SI is applied to flow cytometry data to define immune cell subsets through specific gating strategies and marker expression patterns [27]. By integrating features with the appropriate qualifiers, PhenoDE provides a unified and extensible representation that links measurement objects, processes, and results across platforms.

### 2.3 Assessment of the Standardization and Integrability of Phenome Data Elements

**FIGURE 3:**
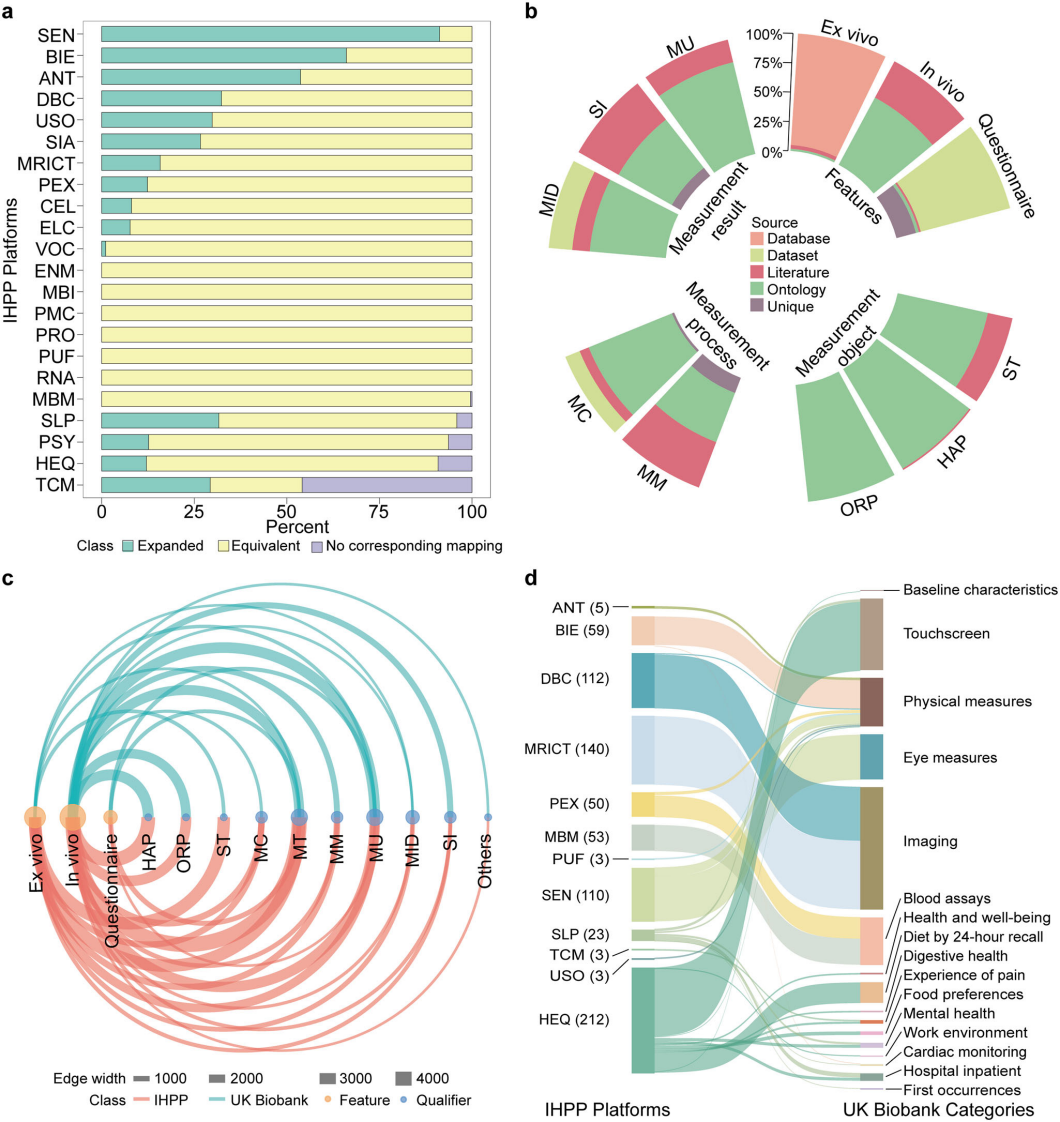
Cross‐referencing and standardization of PhenoDE. (a) Overview of PhenoDE standardization across measurement platforms within the International Human Phenome Project (IHPP), categorized as expanded, equivalent, and no corresponding mapping to assess PhenoAM refinement outcomes (see Methods). Percentages of refinement situations are shown for each platform. SEN, Sensory; BIE, Bioelectricity; ANT, Anthropometry; DBC, DXA body composition; USO, Ultrasonography; SIA, Skin and its appendages; MRICT, MRI & CT; PEX, General physical examination with blood laboratory tests; CEL, Cell; ELC, Eligibility check; VOC, Vocal; ENM, Energy metabolism; MBI, Microbiome; PMC, Plasma metabolic factors and cytokines; PRO, Proteome; PUF, Pulmonary function; RNA, Total RNA and small RNA; MBM, Metabolome; SLP, Sleep; PSY, Psychology; HEQ, Health questionnaire; TCM, Traditional Chinese Medicine. (b) Data specification categories for mapping features and qualifiers. Percentages represent the proportions mapped to each of four source categories by each feature or qualifier class. Databases include GENCODE, UniProt, and other specialized databases. Datasets mainly refer to cohort studies like UK Biobank (UKB) and China Kadoorie Biobank (CKB). Literature encompasses biomedical publications and instrument manuals. Ontologies include the National Cancer Institute Thesaurus (NCIT), the Foundational Model of Anatomy (FMA), and other ontologies. ‘Unique’ denotes novel phenotypes identified by IHPP without existing data source matches. Measurement tools (MT) qualifiers are not included in data standardization mapping. MT qualifiers were retained in PhenoDE but excluded from the present mapping evaluation; therefore, PhenoDEs containing MT as their sole qualifier had no eligible qualifier mapping result. Qualifiers ‘Others’ are not displayed. HAP, human anatomical parts; ORP, orientation and relative positions; ST, sample types; MC, measurement conditions; MM, measurement methods; MID, measurement indicators; SI, statistical indicators; MU, measurement units. (c) Comparison of feature‐qualifier combinations between IHPP and UK Biobank. The upper figure shows UK Biobank combinations, while the lower illustrates IHPP. Circle size reflects quantities, and line widths indicate the number of feature‐qualifier pairings. HAP, human anatomical parts; ORP, orientation and relative positions; ST, sample types; MC, measurement conditions; MT, measurement tools; MM, measurement methods; MU, measurement units; MID, measurement indicators; SI, statistical indicators. (d) Mapping of shared measurements between IHPP and UK Biobank, covering 12 IHPP platforms and 16 UK Biobank ‘Level 1’ categories (data as of September 2023). Notably, UK Biobank does not distinguish individual proteins as separate fields; therefore, the Proteome platform in IHPP is excluded.

Systematic characterization of human phenotypes requires detailed, multidimensional specifications that can be applied across diverse studies. Existing ontologies and phenotype resources provide an essential foundation yet do not fully capture complex traits. To enable structured semantic representation and component‐level mapping, we applied PhenoAM to decompose terms into features and qualifiers and generated controlled vocabulary (CV) mapping records by linking these components to external resources (https://github.com/BioMedBigDataCenter/PhenoAgents). This procedure enabled the assessment of feature and qualifier correspondence as exact match, partial match, or no match across heterogeneous data specifications. Based on the combined feature and qualifier mapping results, PhenoDE‐level outcomes were classified into three categories: expanded, equivalent, and no corresponding mapping (see Methods). The manual refinement procedure demonstrated high agreement with the original CV‐derived classifications (Cohen's kappa = 0.973, Tables S1–S3), supporting the reliability of the classification process. An 18‐platform analysis set included all non‐molecular platforms and the Cell platform. The four molecular platforms (Metabolome, Microbiome, Proteome, and Total RNA and small RNA) were not included in this analysis set because their terms generally corresponded directly to features and these features were mapped predominantly to dedicated molecular databases, while measurement‐context information was represented separately through qualifiers. These molecular platforms were included in the all‐22‐platform analysis reported in Tables S4 and S5. The Cell platform was retained because its features were mainly mapped to the Cell Ontology and supporting literature, and some cell phenotypes additionally involved SI qualifiers, such as those describing cell proportions. Among phenotypes from the 18‐platform analysis set, 36.3% (2461/6786) were categorized as expanded and 61.8% (4194/6786) as equivalent, with pooled mapping coverage of 98.1% (6655/6786) and an unweighted macro‐average mapping rate of 96.4% (Figure 3a and Table S4). Across all 22 platforms, pooled mapping coverage was 99.8% (58 231/58 371), with an unweighted macro‐average mapping rate of 97.0%. The pooled value combines heterogeneous external resource types and should therefore be interpreted as cross‐resource coverage rather than single‐ontology mapping accuracy. A data element term here refers to the original phenotype variable term as defined in the source metadata before feature‐qualifier decomposition by PhenoAM. In addition, among data element terms from the non‐molecular platforms, 20.5% were classified as no match when directly mapped to ontologies (Table S31). When the corresponding PhenoDEs were mapped to multiple external resources, 2.4% were classified as no corresponding mapping, while 97.6% were classified as equivalent or expanded. These results illustrate how representation granularity influences mapping outcomes, as feature‐qualifier‐based linkage can reveal semantic details that may not be captured when data element terms are matched as single units. For example, “Intima‐media thickness of left distal common carotid artery” (PDE078057999.1) only partially matches “Carotid artery intima media thickness” in the Experimental Factor Ontology (EFO).

Standardization of features and qualifiers required links to multiple external data specifications (Figure 3b). In the feature CV for ex vivo measurements, approximately 95% of ex vivo feature mappings were linked to specialized resources such as GENCODE and UniProt, whereas 64.9% of in vivo feature mappings were linked to ontologies and 35.1% to biomedical literature. Most features derived from the MRI & CT platform aligned with the Radiomics Ontology. Questionnaire‐based features mainly mapped to large population cohorts, including the UK Biobank [5] and the China Kadoorie Biobank [28], with 19.6% representing IHPP phenotypes such as microbiota‐related traits. Qualifiers were even more tightly standardized with 80.8% mapped to ontologies and 10.1% supported by literature sources, especially for measurement‐related qualifiers. Within the qualifier HAP, 98.6% mapped to ontologies and 76.4% were linked to the Foundational Model of Anatomy (FMA). A few anatomical qualifiers, such as “Cheilion”, were traced through literature evidence. PhenoAM transformed PhenoDEs into a broadly standardized set of features and qualifiers, and remaining phenotypes without links to existing ontologies, databases, or literature are classified as novel within the current mapping framework. Overall, PhenoAM supports phenotype standardization through systematic component‐level mapping to relevant resources across specialized domains.

Applying PhenoAM to the UK Biobank phenotypes revealed both shared and distinct emphases relative to IHPP. The PhenoAM categories were applied to UK Biobank phenotype descriptions, and assembly of features and qualifiers highlighted common patterns across projects that were largely driven by measurement type (Figure 3c). For instance, in vivo measurements were combined flexibly with nine qualifier groups other than ST. Embedding UK Biobank within PhenoAM enabled systematic mapping to IHPP platforms and exposed fundamental differences in data organization and study design. UK Biobank aggregates imaging phenotypes into a Level 2 Imaging category that includes DXA assessments and three types of MRI‐related measurements, whereas IHPP separates DXA body composition and MRI & CT into dedicated platforms (Figure 3d). PhenoAM makes these contrasts explicit.

IHPP covers a wider spectrum of abdominal imaging phenotypes, including the liver, while UK Biobank provides more detailed brain imaging. Additionally, PhenoAM also illustrates how the same features coupled with different qualifiers encode distinct measurement choices. UK Biobank measures the signal‐to‐noise ratio of the left ear (Field ID: 4233) as an average value, while IHPP records the same feature under diverse measurement conditions. Through decomposition of complex phenotypes into features and qualifiers, PhenoAM provides structured cross‐references between PhenoDEs and heterogeneous phenotype datasets and makes differences in measurement context explicit.

### 2.4 Influence of Descriptive Granularity in Phenotype Data on Population Study Results

In population‐based studies, statistical models typically adjust for covariates such as age, sex, and ethnicity, whereas variation in measurement procedures is rarely considered explicitly. Differences in measurement design, instruments, and protocols can shift data distributions and, in some cases, alter the direction or significance of associations. PhenoAM makes these differences explicit by decomposing phenotypes into features and qualifiers, which allow differences in measurement context to be described, compared, and traced across cohorts.

**FIGURE 4:**
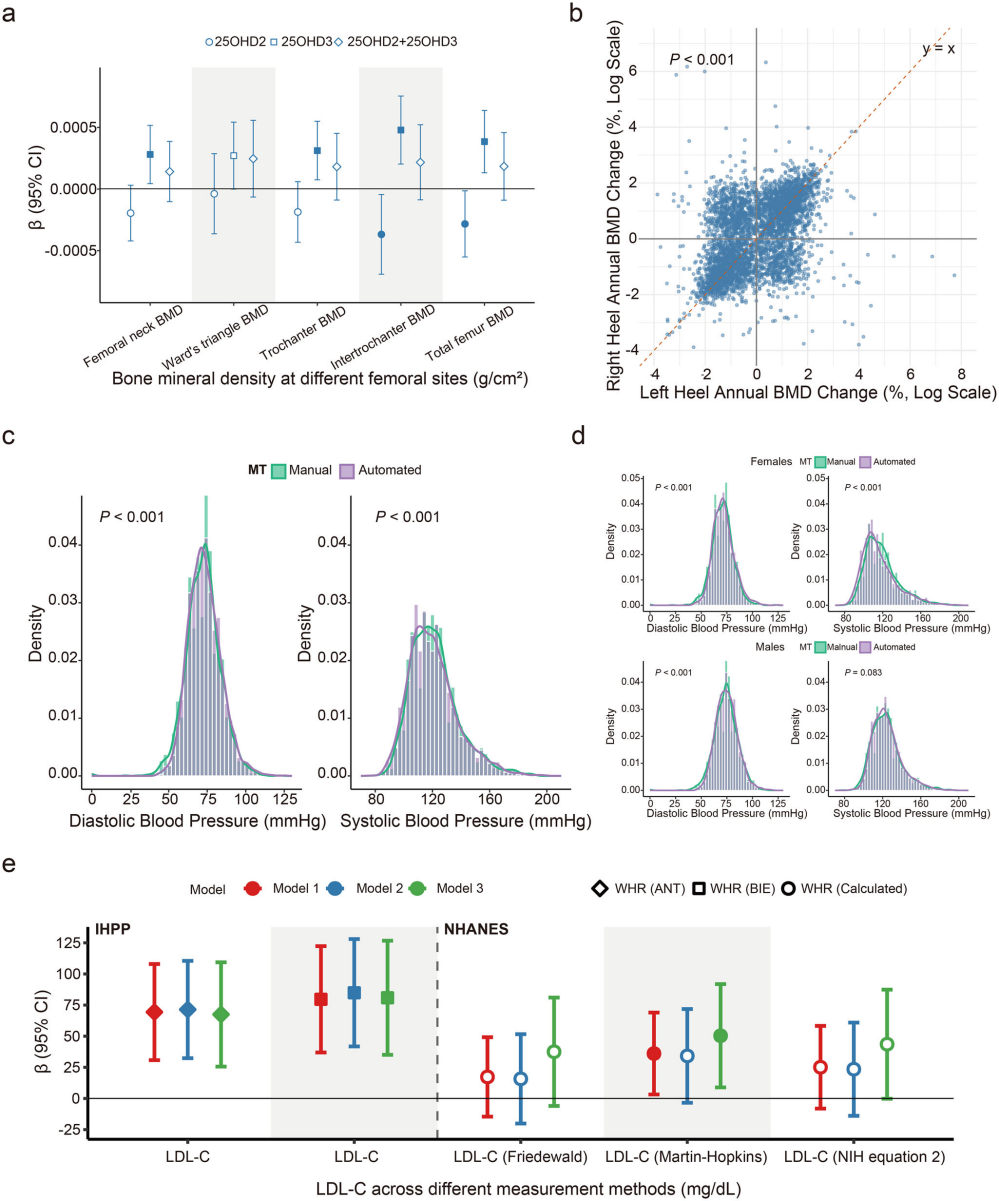
Feature and qualifier distinctions associated with variation in phenotype data characteristics and analytical results. (a) Feature differences in vitamin D measurement in National Health and Nutrition Examination Survey (NHANES). Associations of serum 25‐hydroxyvitamin D_2_ (25OHD_2_), 25‐hydroxyvitamin D_3_ (25OHD_3_), and their total concentration (25OHD_2_ + 25OHD_3_) with bone mineral density (BMD) at four femoral sites and total femur in NHANES, showing different effect directions among vitamin D metabolites. Data are presented as β and 95% confidence intervals (CI). Models were adjusted for age, sex, race, poverty income ratio (PIR), and education. Filled points represent statistically significant associations (*p* < 0.05), while hollow points represent non‐significant associations. (b) Directional differences in BMD measurement in UK Biobank. Comparison of annual percentage changes in calcaneal BMD between left and right heels measured by ultrasound in UK Biobank. (c, d) Instrument differences in blood pressure measurement in NHANES. Characteristics of diastolic and systolic blood pressure in NHANES 2017–2018 measured using mercury sphygmomanometers (manual) and automated oscillometric devices (automated) showed significant differences in the overall population (c) and sex‐specific subgroups (d). Sex‐stratified analyses suggest that diastolic differences were more pronounced among women. MT, Measurement tools. (e) Instrument differences in waist‐to‐hip ratio (WHR) measurement in International Human Phenome Project (IHPP) and calculation method differences in low‐density lipoprotein cholesterol (LDL‐C) measurement in NHANES. In IHPP, WHR obtained from two instruments (Anthroscan Bodyscan from Anthropometry platform [ANT] and Body Composition Analyzer from Bioelectricity platform [BIE]) showed consistent positive associations with serum LDL‐C. Data are presented as β and 95% confidence intervals (CI). Models were adjusted sequentially: Model 1, age and sex; Model 2, Model 1 plus family annual income and education; Model 3, Model 2 plus body mass index (BMI), hypertension, and diabetes. In NHANES, LDL‐C values were calculated using three formulas: Friedewald equation, Martin‐Hopkins equation, and NIH Equation 2. Data are presented as β and 95% confidence intervals (CI). Models were adjusted sequentially: Model 1: age and sex; Model 2: Model 1 plus race, PIR, and education; Model 3: Model 2 plus BMI, hypertension, and diabetes. Filled points represent statistically significant associations (*p* < 0.05), while hollow points represent non‐significant associations.

Feature‐level distinctions can reveal association patterns that may be obscured when related analytes are aggregated or treated as interchangeable. In NHANES, vitamin D‐related phenotypes included serum 25OHD_2_, 25OHD_3_, and total 25OHD. Serum 25OHD_3_ shows positive associations with bone mineral density (BMD) at multiple femoral sites, whereas 25OHD_2_ shows generally negative associations at the same sites, and some of these do not reach statistical significance. Total 25OHD does not show significant associations with BMD (Figure 4a). 25OHD_2_ and 25OHD_3_ are distinct analytes, and the observed patterns may reflect genuine biological heterogeneity, residual confounding, or other unmeasured factors. The role of PhenoAM in this example is limited to preserving and exposing analyte‐specific distinctions so that they are not obscured in phenotype representation and can be addressed in subsequent analyses.

Qualifier‐level differences are also prominent in BMD and blood pressure measurements. For in vivo phenotypes, differences in measurement direction can produce distinct results. In the UK Biobank, annual changes in calcaneal BMD measured by ultrasound differ in magnitude between the left and right heels, and some participants exhibit opposite directions of change (Figure 4b). Similarly, DXA data from IHPP and the UK Biobank demonstrate systematic differences across multiple femoral sites that depend on measurement direction (Figure S3a–j). These findings indicate that even when the features are identical, variation in qualifiers such as direction or specific anatomical site can be associated with divergent estimates.

Measurement instruments, represented by the MT qualifier, provide another key source of heterogeneity. In NHANES 2017–2018, mean systolic and diastolic blood pressure differ significantly between measurements obtained with a mercury sphygmomanometer and an automated oscillometric device (Figure 4c). After stratification by sex, the difference in diastolic blood pressure is driven mainly by women (Figure 4d). These results show that measurements obtained with different instruments can differ in distribution within the same population.

In multi‐cohort analyses, the cumulative effect of feature and qualifier inconsistencies becomes more pronounced. In IHPP, waist‐to‐hip ratio (WHR) obtained from different measurement tools, captured by the MT qualifier, is positively associated with serum low‐density lipoprotein cholesterol (LDL‐C) (Figure 4e). In the UK Biobank, higher WHR is associated with higher levels of LDL‐C measured by both biochemistry and nuclear magnetic resonance (Figure S3k). In contrast, in NHANES, LDL‐C values calculated using three different formulas show variable significance of association with WHR (Figure 4e), illustrating variation in analytical outcomes across calculation methods captured by the MM qualifier. These examples demonstrate that differences in qualifiers can contribute substantially to heterogeneity across studies. When multiple dimensions of measurement vary simultaneously, comparability is further reduced, and results may be affected by both measurement bias and confounding bias, which calls for cautious interpretation.

Overall, these results show that variation in both features and qualifiers can shape phenotype distributions and association estimates in population studies. Applying PhenoAM and PhenoDE makes these differences explicit within a standardized representation, improves data consistency, and supports more comparable and interpretable analyses across cohorts.

### 2.5 PhenoAgents: Large Language Model‐Based Agents for Scalable Phenotype Standardization and Data Quality Assessment

**FIGURE 5:**
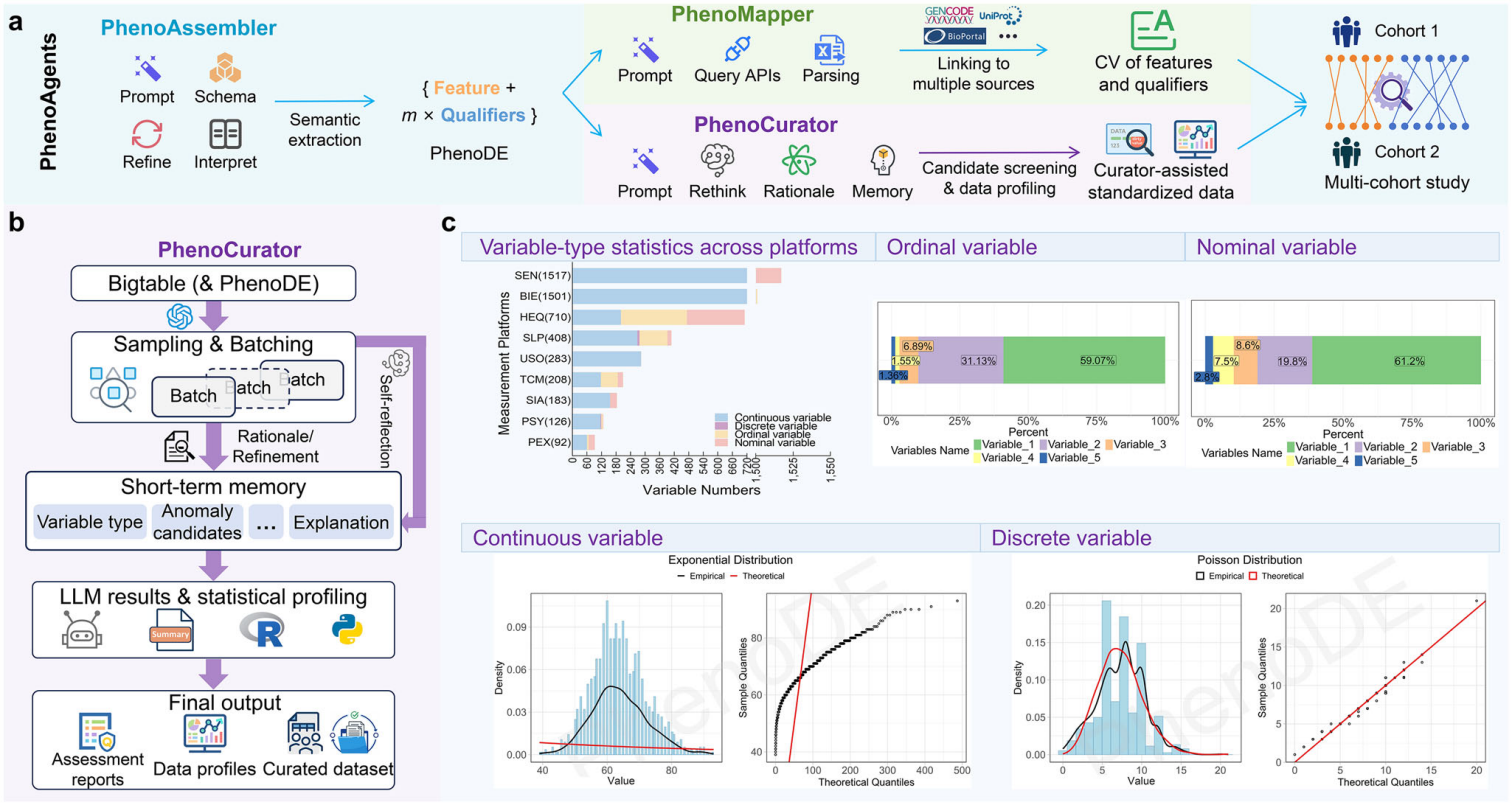
Large language model (LLM)‐based PhenoAgents for phenotype standardization and data quality assessment. (a) The PhenoAgents comprise three cooperative LLM‐based agents that collectively support systematic phenotype standardization and data quality assessment. The PhenoAssembler semantically decomposes phenotype descriptions into measurable features and qualifiers, generating candidate structured PhenoDE representations. The PhenoMapper retrieves and ranks candidate links from external terminologies and domain‐specific databases for curator review. The PhenoCurator generates anomaly candidates and statistical profiles to support downstream review and multi‐cohort analyses. CV, controlled vocabulary. (b) PhenoCurator applies a self‐reflective large language model to classify variable types and identify candidate missing‐value codes and anomalous values. It summarizes the findings in data quality assessment reports and data profiles that guide subsequent review and analysis. (c) Representative results from PhenoCurator showing variable‐type distributions and statistical characteristics of ordinal, nominal, continuous, and discrete phenotype variables across platforms. Detailed summaries and profiles are available on the PhenoDE Portal (https://www.biosino.org/PhenoDE/).

Recent advances in LLMs have enabled scalable semantic interpretation of complex biomedical variables [29, 30], creating opportunities to assist phenotype standardization across cohorts. Building on the semantic and structural foundation provided by PhenoAM and PhenoDE, we developed the PhenoAgents family, a set of three cooperative agents that can operate sequentially to support structured phenotype representation, terminology retrieval, and quality review (Figure 5a). Together, these agents support feature and qualifier extraction, terminology candidate retrieval, and transparent data‐quality review, enabling scalable and traceable cohort data management.

PhenoAssembler performs semantic parsing of phenotype descriptions according to the PhenoAM scheme. Each input phenotype, structured as a data element, is processed with context‐aware prompts that provide definitions, examples, and classification rules that distinguish measurable features from their qualifiers. The agent identifies the core feature and associated qualifiers, assigns them to predefined semantic categories, and outputs candidate structured PhenoDE representations. These representations provide fine‐grained, machine‐readable structure that supports comparison of phenotype descriptions across heterogeneous cohorts.

PhenoAssembler was evaluated using 300 manually curated and expert‐reviewed non‐molecular phenotype representations (Tables S6–S10). Feature‐class assignment was consistently accurate across the four model‐prompt conditions (0.9667–0.9911), whereas exact matching for feature terms was lower (0.3056–0.4044). This pattern indicates that term‐level decomposition was more sensitive to semantic boundaries and annotation granularity than class‐level assignment. Adding explicit PhenoAM rules improved weighted positive macro F1 for qualifier extraction from 0.3690 to 0.6082 with DeepSeek‐V3 and from 0.2976 to 0.5181 with GPT‐4o. To assess whether automatic exact matching underestimated acceptable decompositions, we performed a targeted manual review of 401 automatically non‐exact or internally inconsistent items. The two assessors showed high agreement (exact agreement, 0.9002; Cohen's kappa, 0.8441). In the representative run, assessor‐supported evaluation yielded a feature‐term exact‐match rate of 0.5200 and a weighted feature‐term score of 0.6803, compared with the corresponding automatic estimates of 0.3200 and 0.4247. These findings show high feature‐class accuracy, improved qualifier extraction with explicit rules, and greater sensitivity of feature‐term generation to boundary definition.

PhenoMapper further processes PhenoDE entries generated by PhenoAssembler and retrieves and ranks candidate mappings for each feature and qualifier from controlled terminologies and ontological resources. It dynamically determines whether a concept refers to a molecular or non‐molecular phenotype and queries multiple domain‐specific databases and APIs for semantic cross‐referencing. Through iterative retrieval and ranking, PhenoMapper supports curator review of terminology usage and component‐level linkage across measurement domains and cohorts.

PhenoMapper was evaluated using 200 manually curated and expert‐reviewed reference mappings (Tables S11–S15). GPT‐4o achieved higher recall against the reference mappings than DeepSeek‐V3 under both prompt settings. With the detailed prompt, recall was 72.5% for GPT‐4o and 65.8% for DeepSeek‐V3, with Top‐10 hit rates of 64.2% and 54.0%, respectively. Molecular terms were retrieved more reliably than non‐molecular terms in every condition, and repeated runs showed only small variation. These results indicate that PhenoMapper achieved relatively stable retrieval performance across repeated runs and can narrow the terminology search space for human review. The final mapping decision, particularly for non‐molecular terms, should therefore be made through human review of the ranked candidates.

PhenoCurator performs automated candidate screening and interpretable statistical profiling for large‐scale projects such as IHPP (Figure 5b). Using batched processing and self‐reflective reasoning, it classifies variable types, identifies candidate missing‐value codes and anomalous values, and generates structured quality reports that summarize variable classifications, candidate irregularities, and explanatory rationales. Applied to 7249 non‐molecular phenotype variables across nine measurement platforms, PhenoCurator flagged potential anomaly candidates in approximately 46.6% of variables, a proportion interpreted only as an exploratory candidate‐screening rate. Following PhenoCurator classification and manual review, 866 (11.9%) of the same 7249 variables were categorized as qualitative variables. Statistical analyses and distribution fitting were performed for each variable type (Figure 5c). Statistical characteristics are available on the PhenoDE Portal. Aggregate statistics from the distribution‐fitting procedure, including final fitting statuses and selected distribution families, are reported in Tables S29 and S30. This scalable procedure supports quality review within the evaluated workflows while maintaining analytic resolution at the level of individual variables.

PhenoCurator was evaluated using both a simulation‐based benchmark with predefined anomalies and a real‐data assessment based on NHANES. The simulation benchmark comprised Dataset 1 (Explicit Anomaly Dataset), Dataset 2 (Format and Coding Anomaly Dataset), and Dataset 3 (Semantic‐Context‐Dependent Anomaly Dataset). Across 108 runs covering two models, two prompts, three input‐information levels, and three datasets, DeepSeek‐V3 with Prompt 2 achieved the highest value‐level micro F1 (0.7345) and observation‐point‐level micro F1 (0.7090) after aggregation across input‐information and dataset conditions (Tables S16–S21). GPT‐4o generally produced fewer false‐positive candidate values but missed more predefined anomalies, whereas DeepSeek‐V3 achieved higher recall and F1. Performance varied across prompts, input‐information levels, and datasets, with Dataset 2 showing the lowest overall value‐level F1. Among the evaluated configurations, DeepSeek‐V3 with Prompt 2 provided the best overall balance between precision and recall for anomaly‐candidate detection.

The NHANES assessment included 114 variables from 1000 adults in the 1999–2000 cycle (Tables S22–S28). Under Prompt 2 with Enhanced metadata, DeepSeek‐V3 generated 1016 distinct candidates in the three‐run union, with a candidate‐support rate of 99.8% and review‐derived reference‐set coverage of 48.5%. Meta‐Llama3‐70B‐Instruct generated 1506 distinct candidates in the corresponding three‐run union, with corresponding values of 88.2% and 63.5%. Mean pairwise Jaccard similarity was higher for DeepSeek‐V3 than for Meta‐Llama3‐70B‐Instruct (0.449 versus 0.265). These results indicate that DeepSeek‐V3 produced a smaller and more reproducible candidate set with a higher candidate‐support rate, whereas Meta‐Llama3‐70B‐Instruct covered a larger proportion of the review‐derived reference set. Candidate‐support rate and review‐derived reference‐set coverage were used as descriptive review metrics because the non‐exhaustive NHANES reference set was assembled from reviewed model‐generated candidates and assessor‐identified missed values rather than from exhaustive annotation of all variable‐value pairs. Inter‐assessor agreement was complete for missing‐value‐code labels. In contrast, anomalous‐value candidates showed limited strict three‐category agreement (exact agreement, 0.358; Cohen's kappa, 0.184), with most disagreements occurring between the plausible and not‐endorsed categories, reflecting the context‐dependent nature of anomalous‐value assessment. In the DeepSeek‐V3 input‐information analysis, conditional variable‐type accuracy increased from 0.932 with Name only to 0.962 with Basic metadata and 0.965 with Enhanced metadata, indicating that most of the gain was obtained after adding variable metadata.

Across the three agents, PhenoAssembler provided relatively stable class‐level phenotype decomposition, PhenoMapper generated ranked terminology candidates with relatively stable retrieval performance, and PhenoCurator prioritized candidate data‐quality issues for review. Together, these evaluations support the use of PhenoAgents as curator‐assisted modules for phenotype representation, terminology candidate retrieval, and data‐quality review. These modules reduced the search and review space at different stages of curation and generated structured outputs for review before final phenotype representation, terminology mapping, or data‐quality decisions.

## 3 Discussion

Deep phenotyping requires not only standardized terminology but also fine‐grained and consistent representation of how phenotypes are defined and measured. In cohort studies, essential semantic information is often distributed across variable labels, definitions, collection protocols, sample metadata, instrument documentation, and data‐processing records, with uneven granularity across platforms and projects. PhenoAM represents the measurable characteristic as a feature and organizes information about the measurement object, process, result, and applicable subject as typed qualifiers. PhenoDE organizes these combinations as identifiable and reusable data elements, while PhenoAgents support their construction, external‐resource linkage, and quality review. The typed organization of measurement objects, processes, results, and applicable subjects provides a consistent variable‐level representation of measurement semantics that are otherwise distributed across heterogeneous metadata sources. Together, the framework provides a standardized compositional representation for explicit phenotype description and semantic comparison, with potential value for cross‐cohort phenotype harmonization.

This structure preserves shared semantic components while keeping domain‐specific measurement detail explicit. The same feature can be incorporated into related but distinct PhenoDEs when anatomical site, biospecimen, condition, method, instrument, or result processing differs. Component‐level mapping showed that different semantic components may link to different external resources, reflecting the complementary coverage of terminologies, databases, and other specifications. Such linkage helps trace which aspects of a variable are represented across resources, while remaining distinct from complete semantic equivalence or established cross‐site interoperability. The distribution of semantic components across heterogeneous external resources further reflects the complexity of cross‐cohort phenotype integration. The 99.8% pooled mapping coverage across all 22 IHPP platforms represents broad cross‐resource coverage, reflecting the ability of the framework to connect heterogeneous phenotype representations with complementary external resources. Previous multi‐cohort studies and harmonization initiatives have identified biospecimen handling, analytical platforms, assay procedures, and outcome definitions as contributors to heterogeneity [31, 32, 33]. Consistent with this, our analyses showed that differences in analyte identity, anatomical location, instrument, and calculation method were associated with variation in distributions or association estimates, underscoring the value of explicit measurement context.

Existing biomedical standards and information models support different but connected functions in representing research variables and their associated observations [34, 35]. For semantic concepts and terminology, HPO provides ontology terms and relations for abnormal human phenotypes [9]. LOINC provides standardized identifiers for laboratory and clinical observations, while SNOMED CT supports clinical concept representation and formal post‐coordination (Note S3). For individual components, PhenoAM feature and qualifier terms can be linked to such terminology or ontology concepts where appropriate, allowing established semantic identifiers to be reused. For data‐element definition, registration, and management, ISO/IEC 11179 provides a metadata‐registry framework for data‐element concepts, representations, and value domains, while NIH CDE and NCI caDSR provide reusable biomedical data‐element definitions and associated metadata [11, 35, 36]. Within this data‐element representation, each PhenoDE is an identifiable data‐element definition, and PhenoAM structures its measurement semantics through a feature and typed qualifiers describing the measurement object, process, result, and applicable subject. These components can in turn be linked to external terminology and ontology resources, consistent with the use of terminology systems for semantic annotation in existing data‐element repositories [36]. For representation and exchange of individual‐level phenotype and observation records, Phenopackets provides a machine‐readable structure for individual‐ and biosample‐level phenotypic and measurement information [10]. FHIR Observation provides a structured resource for observed values together with associated context such as subject, time, specimen, method, device, and body site (Note S4) [37]. In the current implementation, participant‐level measurement data are linked to the corresponding PhenoDE identifiers in the cohort data layer. Potential integration of PhenoDE with record models such as FHIR Observation or Phenopackets would require formal mappings and implementation‐specific alignment [37]. A generic Entity‐Attribute‐Value model provides a flexible storage pattern for heterogeneous attributes, whereas the typed feature and qualifier structure assigns explicit phenotype‐specific semantic roles to these attributes. This organization provides a structured basis for linking research‐variable definitions with established terminology resources, data‐element systems, and individual‐level observation models.

PhenoAgents provide computational support for applying this representation to large heterogeneous datasets. PhenoAssembler, PhenoMapper, and PhenoCurator support, respectively, semantic decomposition, terminology candidate retrieval, and prioritization of potential data‐quality issues. Feature‐class assignment was relatively stable across the evaluated conditions, explicit PhenoAM rules improved qualifier extraction, and exact feature‐term agreement was more sensitive to annotation granularity and expression choices. Manual review of outputs classified as non‐exact by automated scoring indicated that strict exact‐match evaluation may underestimate semantically acceptable decompositions. PhenoMapper helped narrow the terminology search space, whereas PhenoCurator generated reviewable anomaly candidates, with candidate‐detection performance varying across models, prompts, metadata inputs, and anomaly types. These results support its current role as a curator‐assisted candidate‐screening tool rather than an autonomous quality‐control system, helping to prioritize potential data‐quality issues for subsequent human review. The current workflow combines model‐assisted processing with targeted human review, enabling repetitive retrieval, annotation, and screening tasks to be performed at scale while retaining human oversight for key semantic and data‐quality judgments. Model and prompt configurations may also need to be adapted to the metadata available in individual cohorts and to specific task requirements. Further improvements in accuracy and efficiency will depend on continued task‐specific evaluation and adaptation alongside advances in foundation models.

Further work should focus on the generalizability and operational adoption of the framework beyond its current implementation. The complete framework has been implemented primarily within the IHPP data‐management environment. Analyses using UK Biobank and NHANES data provide useful evidence that the structured representation can be applied to phenotypes from other large cohort resources, while independent implementation and cross‐site validation remain areas for further evaluation. Further application across diverse cohorts and data environments will help establish its broader applicability and generalizability. In particular, evaluation in substantially different clinical data environments, including EHR‐derived data structured using the OMOP Common Data Model, would further extend the assessment of the framework across different phenotype‐data settings.

Current PhenoAgents benchmarks cover selected models, prompts, datasets, and task settings, and larger prospective evaluations should further examine performance, stability, computational cost, and human‐review burden under operational conditions. As LLM‐based models and agents continue to improve, they may increasingly support complex and repetitive standardization tasks that would otherwise impose substantial manual burden, making fine‐grained frameworks such as PhenoAM and PhenoDE more practical to implement at scale. Beyond these implementation considerations, clinical settings may also involve phenotypes derived from multiple observations, thresholds, temporal patterns, or diagnostic rules. Supporting such derived phenotypes may require extension of the current qualifier categories and more explicit representation of computational and decision rules. The current JSON Schema supports structural validation and selected composition constraints but not full ontology‐level reasoning or automated semantic‐equivalence testing. Sustained and cross‐site use will require stable identifiers and versioned, traceable maintenance of external mappings, together with alignment to local data models, workflows, metadata availability, and institutional governance and privacy requirements.

Overall, PhenoAM organizes measurement semantics distributed across variable descriptions and associated metadata into a consistent and reusable structure. PhenoDE provides a large‐scale, multi‐domain implementation of this approach, while PhenoAgents support its construction, linkage, and review. By preserving domain‐specific measurement detail within a standardized representation of measurement objects, processes, and results, the framework provides a clearer semantic basis for phenotype harmonization and cross‐platform comparison and creates a practical foundation for future integration with established terminology and data‐exchange standards.

## 4 Methods

### 4.1 Phenotype Assembly Method (PhenoAM)

Despite the increasing complexity of phenotypic research across diverse domains, there remains a notable lack of standardized formats for describing and capturing these intricacies systematically. To bridge this critical gap, PhenoAM decomposes each phenotype data element into exactly one “feature” and one or more associated “qualifiers” (Feature + *m* × Qualifiers, where m ≥ 1). Drawing conceptual inspiration from the Feature Table format used by the International Nucleotide Sequence Database Collaboration (INSDC), which describes genomic elements and domains [38], PhenoAM applies a similar structured framework to support clarity and interoperability in phenotype description across diverse research disciplines.

In PhenoAM, “feature” represents the core measurable characteristic of a phenotype and is categorized into ex vivo, in vivo, and questionnaire measurements. A feature denotes the core measurable characteristic represented by a PhenoDE. The feature term should be concise while retaining a biologically meaningful and reusable measurand; a compound noun phrase may therefore be retained when further simplification would make the concept overly generic or alter its biological meaning. A qualifier is a typed contextual component that further specifies the feature with respect to the measurement object, process, result, or applicable subject. Operationally, a component is retained within the feature when its removal changes the identity or biological meaning of the measurand; otherwise, it is assigned to the appropriate qualifier category.

In IHPP, features are classified as ex vivo, in vivo, or questionnaire measurements, whereas qualifiers are organized into 11 categories related to the measurement object, process, and result (Table 1). Each PhenoDE contains exactly one feature and one or more qualifiers. The ST qualifier is mandatory for ex vivo records, and an ex vivo record without an ST qualifier is considered incomplete. Regarding combination constraints, qualifier categories are not mutually exclusive and may co‐occur within the same PhenoDE. Within a single qualifier category, multiple values are permitted when jointly applicable.

**TABLE 1:** Classes and Definitions of Qualifiers in the Phenotype Assembly Method (PhenoAM).

Class	Subclass	Abbreviation	Descriptions	Examples
Measurement Object	Sample Types	ST	Types of tissues, fluids, and cells collected for ex vivo measurements.	Plasma, Urine
	Human Anatomical Parts	HAP	Specific regions or parts of the human body used to describe anatomical locations.	Brain, Heart
	Orientation and Relative Positions	ORP	Direction and position of objects or structures relative to one another.	Left, Center
	Applicable Subjects	AS	Describes the applicable populations, their relationships to subjects, or subject attributes.	Female, Child
Measurement Process	Measurement Methods	MM	A set of standards and procedures that outline the requirements for measurement operations, including computational processes.	Bone conduction, Fridericia formula
	Measurement Tools	MT	Tools or equipment used to measure, observe, or record data.	Electrocardiograph, Fatigue Severity Scale
	Measurement Conditions	MC	Variables and factors influencing measurement results, including environmental requirements, timing, subject status, and instrument settings.	500 Hz, Panel 1
Measurement Result	Measurement Indicators	MID	Measurement values used to assess or quantify specific properties.	Forward scatter, Skin area
	Statistical Indicators	SI	Quantitative indicators and distributions derived from statistical calculations.	Maximum, Average
	Measurement Units	MU	Standard units used to measure or quantify specific measurement objects.	mg/L, mmHg
Others	Others		Classifications or additional information not included in the above categories.	Recommended

PhenoDEs undergo semantic segmentation and lemmatization to ensure standardization, with features extracted from phenotype terms and qualifiers derived from phenotype definition and associated measurement details. For questionnaire‐derived phenotypes, the feature is represented by a standardized summary that encapsulates the core phenotype term. Ex vivo measurements encompass nucleic acids, proteins, metabolites, or cells, distinct from their sample type and methodologies. Detailed PhenoAM annotation rules and worked examples are provided in Note S5. All features and qualifiers originate from rigorously curated controlled vocabularies and are validated through the Human Phenome Project Technological Advisory Groups.

In summary, PhenoDE construction followed a sequential workflow from source definition to normalized term, PhenoAM semantic decomposition, and standardized structured representation. Figure S4a illustrates this workflow using an IHPP example.

### 4.2 Alignment of Phenome Data Elements With Standardized Specifications

By applying PhenoAM to PhenoDEs, we systematically classified features and qualifiers, aligning them with internationally standardized phenotype‐related specifications. This alignment leveraged specialized databases, datasets, ontologies, and biomedical literature, employing methods such as web scraping, assisted manual matching, proofreading, and database reconciliation. Our database integration encompassed 15 distinct resources, including GENCODE, UniProt, LIPID MAPS, and BioCyc, while 55 ontologies such as LOINC, NCIT, SNOMED CT, FMA, OBI, CMO, and MeSH were incorporated. Additional details are available at https://bioportal.bioontology.org/. Key datasets included 15 sources, notably the UK Biobank, China Kadoorie Biobank, and China Health and Nutrition Survey. We also mapped 337 references and conducted comprehensive mappings with the UK Biobank.

To support machine‐verifiable exchange, we formalized PhenoDE as a JSON‐based information model and used JSON Schema Draft 2020‐12 as the normative structural schema. The model specifies core metadata, stable identifiers, schema and record versions, Variable IDs, an optional Value domain for enumerated variables, one feature, and one or more jointly constraining qualifiers. The schema requires at least one ST qualifier for ex vivo records and restricts Value domain to Enum‐type records. External mappings are maintained with source and identifier fields, version information when it can be verified, and curator‐reviewed updates for deprecated, broken, unresolved, or ambiguous identifiers. The complete schema, field specifications, representative instances, and external resource maintenance policy are available in the GitHub repository (https://github.com/BioMedBigDataCenter/PhenoAgents). External‐resource maintenance policies and source‐version information are also available on the PhenoDE Portal (https://www.biosino.org/PhenoDE/about).

Features and qualifiers aligned with standardized specifications were categorized into three types: exact match, partial match, and no match. An exact match reflects full semantic alignment despite variations in wording, with no loss of essential meaning. A partial match captures some, but not all, semantic elements because of limited detail in the specification or nuanced feature complexity. No match may represent novel attributes for which no existing specification corresponds.

In Table 2, “Only qualifier MT” denotes records for which MT was the sole qualifier. MT was excluded from external‐resource mapping statistics because stable terminology‐level mappings are not consistently available for specific instruments and software; therefore, no qualifier mapping result was assigned. This label does not indicate that the PhenoDE lacked qualifiers. MT nevertheless remains part of the PhenoAM representation and is retained in phenotype‐data‐element‐level comparisons across cohorts.

**TABLE 2:** Classification of PhenoAM‐Transformed PhenoDE Mapping Results.

Category	Feature Mapping Result	Combined Qualifier Mapping Result
Equivalent	Exact match	Only qualifier MT
	Partial match	Only qualifier MT
	Exact match	Exact match
Expanded	Exact match	Partial match
	Exact match	No match
	Partial match	Exact match
	Partial match	Partial match
	Partial match	No match
No corresponding mapping	No match	Only qualifier MT
	No match	Exact match
	No match	Partial match
	No match	No match

To assess the impact of PhenoAM on refining PhenoDE descriptions, we quantified mapping outcomes across 12 distinct result sets. When multiple qualifiers were present, the combined qualifier mapping result was classified as exact match if all eligible qualifiers were exact match, no match if all were no match, and partial match for all other combinations. The categorized results are summarized in Table 2. Mapping outcomes were summarized using both pooled mapping coverage and the unweighted macro‐average mapping rate. Detailed definitions, calculation procedures, and results are provided in the Supplementary Methods and Results Section 1.

For cross‐cohort comparison, phenotype variables were compared at the phenotype data element level using the feature and all applicable qualifiers represented in the source metadata, including MT. This comparison preserved differences in measurement context rather than relying on feature agreement alone. IHPP and UK Biobank variables were first represented using the same PhenoAM scaffold and then compared component by component. Feature correspondence was evaluated separately from the overall correspondence across all applicable qualifiers. Shared components supported semantic correspondence, whereas differing or unavailable components were retained explicitly rather than being treated as equivalent. Representative examples of semantic decomposition and component‐level comparison between IHPP and UK Biobank are shown in Figure S4b.

### 4.3 Investigating Feature and Qualifier Differences in Population Studies

We used data from the NHANES, UK Biobank and IHPP to evaluate the impact of feature or qualifier differences under different scenarios. Detailed descriptions of the study design and data collection for the UK Biobank [39] and NHANES (available from: https://wwwn.cdc.gov/nchs/nhanes/analyticguidelines.aspx#analytic‐guidelines) have been reported previously.

Data from three NHANES cycles (2007–2010, 2013–2014, and 2017–2018) were included. The association between vitamin D metabolites and BMD was examined among non‐pregnant adults. Participants with complete measurements of 25OHD_2_, 25OHD_3_, total vitamin D, and femoral BMD at five sites were included. Those with missing covariates were excluded, and a total of 2742 participants remained for analysis. Linear regression was conducted to assess the associations. Subsequently, two separate analyses were conducted using data from NHANES 2017–2018, one focusing on blood pressure and the other on LDL‐C. In the first analysis, blood pressure obtained by manual measurement was compared with that obtained by automated measurement. We included participants who underwent blood pressure assessment using both a mercury sphygmomanometer and an Omron HEM‐907XL device. Participants were excluded if the interval between the two measurement methods exceeded 2 h or if they were taking antihypertensive medications. Finally, a total of 2733 participants were included. Mean systolic and diastolic blood pressure were calculated according to NHANES analytic protocols (https://wwwn.cdc.gov/Nchs/Data/Nhanes/Public/1999/DataFiles/BPX.htm#Analytic_Notes). The Wilcoxon signed‐rank test was used to compare differences in blood pressure between the two measurement methods. In the second analysis, we examined whether the use of different LDL‐C calculation methods led to differences in association results. A total of 1841 fasting participants with complete covariates were included, all of whom had waist and hip circumference measurements as well as LDL‐C values derived from three calculation formulas (LBDLDL, LBDLDLM, and LBDLDLN). Linear regression was used to examine the association between waist‐to‐hip ratio (WHR) and LDL‐C.

Heel ultrasound BMD data from 4329 participants in the UK Biobank were used to examine differences in the annual rate of BMD change between the left and right sides. Annual percentage changes for each side were calculated using baseline and first follow‐up measurements. Comparisons between sides were performed using the Wilcoxon signed‐rank test.

We utilized data from the IHPP to assess differences in the associations of WHR, as measured by different instruments, with LDL‐C. A total of 775 participants in IHPP with both WHR and serum LDL‐C measurements were included. WHR was obtained using Anthroscan Bodyscan and Body Composition Analyzer, while serum LDL‐C was measured with a Sysmex Fully Automated Analyzer on the General Physical Examination with Blood Laboratory Tests (PEX) platform. Linear regression was applied to assess the association.

All analyses were performed using R software (version 4.1.3), and a two‐sided *p* value <0.05 was considered statistically significant.

### 4.4 PhenoAgents for Phenotype Standardization and Data Quality Assessment

To enhance the scalability and usability of the phenotype standardization framework, we developed a suite of LLM‐based agents collectively termed PhenoAgents. PhenoAgents comprise three LLM‐based components that support phenotype semantic decomposition, terminology candidate retrieval, and quality‐control candidate generation. PhenoAssembler converts phenotype descriptions into structured feature and qualifier representations, PhenoMapper retrieves and ranks candidate mappings to relevant external resources, and PhenoCurator profiles observed values and identifies candidates requiring quality‐control review. The components can be used sequentially, but each produces an independently inspectable structured output and was evaluated using a task‐specific reference standard.

All agents used structured prompts and machine‐readable output schemas, followed by parsing and validation of generated outputs before downstream use. In the evaluation experiments, each reported condition was run independently three times at a temperature of 0.2 unless otherwise specified. The Supplementary Methods and Results Section 2 describe benchmark construction, data preparation, normalization, scoring, and human‐review procedures and provide condition‐level results. The exact unabridged prompt texts and corresponding scripts are available in the public GitHub repository cited in the Code Availability Statement.

#### 4.4.1 PhenoAssembler for Large Language Model‐Assisted Semantic Decomposition and Evaluation

PhenoAssembler receives a phenotype description together with its available data‐element definition and contextual metadata. The prompt defines the PhenoAM task, the distinction between a core measurable feature and contextual qualifiers, the permitted qualifier categories, and the required output fields. The agent identifies one feature and the associated typed qualifiers and returns a structured JSON record. The record is checked for required fields and converted into a tabular PhenoDE representation for subsequent mapping and data integration. PhenoAssembler supports first‐pass semantic decomposition rather than replacing curation; outputs remain reviewable against the original phenotype description and controlled vocabularies before being retained as PhenoDE records.

PhenoAssembler was evaluated using an independent set of 300 non‐molecular phenotype records sampled after records used for prompt optimization had been excluded. The set comprised 143 in vivo, 119 questionnaire, and 38 ex vivo measurements. Reference representations were manually curated and expert‐reviewed by the Human Phenome Project Technological Advisory Groups. Evaluation considered feature class, feature term, and qualifier‐term extraction across eight target qualifier categories. Sample type, measurement tool, and measurement unit were supplied as contextual metadata but were not scored as target qualifier outputs.

DeepSeek‐V3 and GPT‐4o were tested with rule‐enhanced and concise prompts. The rule‐enhanced prompt specified PhenoAM definitions, feature‐qualifier boundary rules, category constraints, and structured‐output requirements, whereas the concise prompt retained the task and output structure with fewer explicit semantic rules. Each model‐prompt condition was run three times on the same test set. Performance was assessed using feature‐class accuracy, exact and weighted feature‐term scores, and category‐level weighted precision, recall, and F1 for qualifier terms. A targeted scoring‐sensitivity analysis was also conducted for 401 automatically non‐exact or inconsistent items from a representative run whose automatic metrics were close to the corresponding three‐run means. Two assessors independently classified these items as correct, partially correct, or incorrect; concordant labels were used for score recalculation, whereas discordant items retained their prespecified automatic scores. Detailed normalization, weighting, matching, and review rules are provided in the Supplementary Methods and Results Section 2.

#### 4.4.2 PhenoMapper for Terminology Candidate Retrieval and Evaluation

PhenoMapper receives a feature or qualifier term together with its semantic category and available platform and item metadata. It first assigns the term to a molecular or non‐molecular retrieval route. Molecular routes query domain‐specific resources for genes, proteins, metabolites, microbes, and related entities. Non‐molecular routes query BioPortal using category‐specific ontology priorities and may use PubMed as a fallback when no candidate is returned, or no adequate label match is found. Candidate records are standardized, deduplicated, and ranked using category‐specific rules, and up to 10 candidates are returned with identifiers, labels, sources, and supporting metadata. Model‐generated semantic judgments and confidence scores are retained as auxiliary information and are not treated as final mapping decisions.

The fixed evaluation set comprised 200 reference mappings, including 80 feature terms and 120 qualifier terms. These mappings were manually curated and expert‐reviewed by the Human Phenome Project Technological Advisory Groups before PhenoMapper was run. The reference mappings were withheld from candidate generation and used only for evaluation. DeepSeek‐V3 and GPT‐4o were tested with detailed and concise prompts. The detailed prompt included category definitions, examples, resource‐selection rules, and ranking principles; the concise prompt retained the same category set, output schema, and core ranking rules with fewer definitions and examples. Each model‐prompt condition was run independently three times using the same fixed resource configuration. Reference‐standard recall measured whether a curated reference appeared among the returned candidates, and Top‐k hit rates described its rank. Matching was based on identifiers, links or internationalized resource identifiers (IRIs), normalized labels or synonyms, and publication evidence where applicable. Full resource routes, ranking, deduplication, and matching rules are described in the Supplementary Methods and Results Section 2.

#### 4.4.3 PhenoCurator for Quality‐Control Candidate Generation, Statistical Profiling, and Evaluation

PhenoCurator processes each phenotype variable using its observed values and available metadata, including the variable name, description, data type, and PhenoAM fields when available. Values are read as strings, and unique observed values are supplied to the model. Variables with many distinct values are processed in batches, with the validated structured result from the preceding batch provided as context for the next batch. Generated JSON is parsed, cleaned of control characters, checked against the required schema and observed values, and regenerated when validation fails. PhenoCurator classifies variables as quantitative (continuous or discrete) or qualitative (ordinal or nominal) and returns candidate missing‐value codes, candidate anomalous values, and explanatory rationales. These outputs are used to prioritize review and are not interpreted as confirmed data errors without supporting evidence. The original IHPP screening workflow applied the unmodified Meta‐Llama‐3‐70B‐Instruct model to 7249 non‐molecular phenotype variables across nine measurement platforms.

Statistical profiling was conducted according to variable type. Quantitative variables were visualized using box plots and histograms. For continuous variables, eligible normal, log‐normal, exponential, and Cauchy distributions were fitted by maximum likelihood. Log‐normal models were evaluated only for strictly positive observations, and exponential models only for nonnegative observations. The Kolmogorov–Smirnov statistic and its nominal *p* value were used as exploratory goodness‐of‐fit screening diagnostics because distribution parameters were estimated from the same observations. For discrete count variables, Poisson and negative binomial distributions were fitted by maximum likelihood and evaluated using a chi‐square goodness‐of‐fit test after pooling categories with low expected counts where feasible. Candidate distributions with a goodness‐of‐fit *p* value greater than 0.05 were retained for comparative model selection, and the retained distribution with the lowest Akaike Information Criterion was selected. The Bayesian Information Criterion was calculated as a supplementary comparative measure and was not used for selection. Variables for which no candidate satisfied the screening criterion were recorded as having no adequate parametric fit. When a valid discrete goodness‐of‐fit assessment could not be calculated, the result was recorded as goodness‐of‐fit test not applicable. Qualitative variables were summarized using stacked bar charts to represent observed category proportions. Further preprocessing, model‐applicability, fitting, diagnostic, and output rules are described in the Supplementary Methods. All statistical analyses and visualizations were performed in R version 4.3.1.

The simulation‐based evaluation used metadata from 200 representative non‐molecular PhenoDE variables. For each variable, 1000 synthetic observations were generated according to variable type and prespecified plausible ranges or valid categories, producing a 200 × 1000 observation matrix. Variable‐specific anomaly templates were applied at known matrix positions to create three nested datasets representing different anomaly scenarios. Dataset 1 (Explicit Anomaly Dataset) contained boundary‐defined explicit anomalies (10 000 anomalous observations; 5%). Dataset 2 (Format and Coding Anomaly Dataset) retained all Dataset 1 anomalies at the same matrix positions and added format, coding, and textual‐expression anomalies (20 000 anomalous observations; 10%). Dataset 3 (Semantic‐Context‐Dependent Anomaly Dataset) retained the anomalies from the preceding datasets and added anomalies requiring interpretation of phenotype semantics, measurement units, valid ranges, or domain knowledge (30 000 anomalous observations; 15%). The nested design (Dataset 1 ⊆ Dataset 2 ⊆ Dataset 3) preserved traceability of anomaly types and matrix positions across datasets. Counts of unique anomalous values and detailed data‐generation rules are provided in the Supplementary Methods and Results Section 2.

DeepSeek‐V3 and GPT‐4o were evaluated using a factorial design comprising two prompts, three input‐information levels, and the three datasets. Prompt 1 specified baseline quality‐control criteria, whereas Prompt 2 retained these criteria and added more explicit rules for distinguishing missing‐value codes from potential anomalies and for interpreting non‐standard representations. Both prompts instructed the model not to classify a value as anomalous solely because it was statistically extreme. Observed values were supplied under all input‐information conditions. Name only included the variable name or identifier. Basic metadata additionally included the variable class, description, data type, unit or valid range, and available enumerations when applicable. Enhanced metadata further incorporated structured phenotype semantics generated by PhenoAM, including available feature and qualifier information such as anatomy, orientation, measurement tool, and unit. The 36 model‐prompt‐input‐dataset conditions were each run independently three times, yielding 108 runs. Value‐level and observation‐point‐level micro‐averaged precision, recall, and F1 were calculated from the predefined reference labels.

The real‐data evaluation used 114 variables from the NHANES 1999–2000 cycle and a random sample of 1000 adults aged at least 20 years. For the cross‐model comparison, DeepSeek‐V3 and a locally deployed Meta‐Llama‐3‐70B‐Instruct model were applied to the same variables under Prompt 2 and the Enhanced metadata condition, with each model run independently three times. For the input‐information comparison, DeepSeek‐V3 was applied to the same variables under Prompt 2 using the Name only, Basic metadata, and Enhanced metadata conditions, with each condition run independently three times, and all other inference settings held constant.

Model‐generated candidate missing‐value codes and candidate anomalous values were normalized and deduplicated at the variable, candidate‐type, and canonical‐value levels, then pooled and source‐masked before review. Two assessors with experience in phenotype metadata curation and data‐quality assessment independently reviewed the candidates using prespecified criteria and the official NHANES codebook as the primary evidence source. Candidates were classified as confirmed, plausible, or not endorsed, with candidate missing‐value codes and candidate anomalous values assessed separately. Candidate‐support rate was defined as the proportion of generated candidates classified as confirmed or plausible. Review‐derived reference‐set coverage was calculated against a non‐exhaustive set assembled from reviewed candidates and assessor‐identified missed values. Because all possible variable‐value pairs were not exhaustively annotated, these measures were not interpreted as conventional precision and recall. Inter‐assessor agreement was calculated from the initial independent annotations, and run‐to‐run stability was summarized using pairwise Jaccard similarity across the three runs.

### 4.5 The Phenome Data Elements Website Architecture and Technology Stack

The PhenoDE Portal employs a browser‐server architecture, utilizing Java, HTML, CSS, and JavaScript, with data storage facilitated by MySQL and full‐text search powered by Elasticsearch. The back‐end framework integrates Spring Boot, Spring MVC, MyBatis Plus, and Spring Data Elasticsearch, while the front‐end leverages Thymeleaf, jQuery, Bootstrap, ECharts, and jQuery UI to deliver an interactive user experience. This platform provides streamlined access to PhenoDEs and phenotype variables, unified under a standardized coding system, with comprehensive feature metadata, qualifiers, and variable statistics, enhancing data exploration and interoperability.

## Author Contributions

G.Q.Z., L.J., and J.C.L. conceived and designed the study. W.T.H. and Y.C.L. proposed PhenoAM. W.T.H., X.Y.H., W.C., and R.Z. applied PhenoAM to PhenoDE construction and standardization of PhenoDE. X.H.Z. and W.T.H. designed PhenoAgents; W.T.H. implemented and evaluated them. J.L.Z. assisted with the statistical analysis of phenotype variables. Y.F.J. and X.D.C. advised on statistical considerations for the cohort analyses of NHANES, UK Biobank, and IHPP. W.T.H., W.C., and Y.C.L. designed the PhenoDE Portal. W.T.H., W.C., X.Y.H., and R.Z. wrote the manuscript with feedback from G.Q.Z. and Y.C.L., while L.J., J.C.L. and X.D.C. provided advice. All authors reviewed the manuscript.

## Conflicts of Interest

The authors declare no conflicts of interest.

## Code Availability Statement

The custom code and scripts developed for this study are publicly available at https://github.com/BioMedBigDataCenter/PhenoAgents. The repository also provides the PhenoDE JSON Schema and representative instances.

## Supporting information




**Supporting File 1**: advs77730‐sup‐0001‐SuppMat.pdf.

**Supporting File 2**: advs77730‐sup‐0002‐SuppMat.pdf.

## Data Availability

Controlled vocabularies of features and qualifiers are provided in the GitHub repository (https://github.com/BioMedBigDataCenter/PhenoAgents), while PhenoDEs from IHPP Phase I are accessible through the PhenoDE Portal (https://www.biosino.org/PhenoDE). Further information and requests for resources should be directed to the lead contact, Dr. Guoqing Zhang (gqzhang@sinh.ac.cn). Resource requests will be fulfilled by the Human Phenome Project Technological Advisory Groups. Large‐scale population cohort data were used in this study. NHANES data are available at https://wwwn.cdc.gov/nchs/nhanes/default.aspx, and UK Biobank data are available on application at http://www.ukbiobank.ac.uk/. The NHANES study received approval from the NHANES institutional review board and National Center for Health Statistics Research ethics review board. The UK Biobank study was approved by the National Health Service North West Multicentre Research Ethics Committee (REC Reference: 21/NW/0157). The UK Biobank application number for this study is 77803.
