# Supplementary material for: Towards a Compositional Framework for Describing Human Phenotypes
Source: Adv Sci (Weinh). 2026 Sep 22:e77730. Online ahead of print. doi: 10.1002/advs.77730 (PMC13598380; doi:10.1002/advs.77730)
Supplement: Supplementary file 1 [file ADVS-9999-e77730-s001.pdf]

Supplementary figures

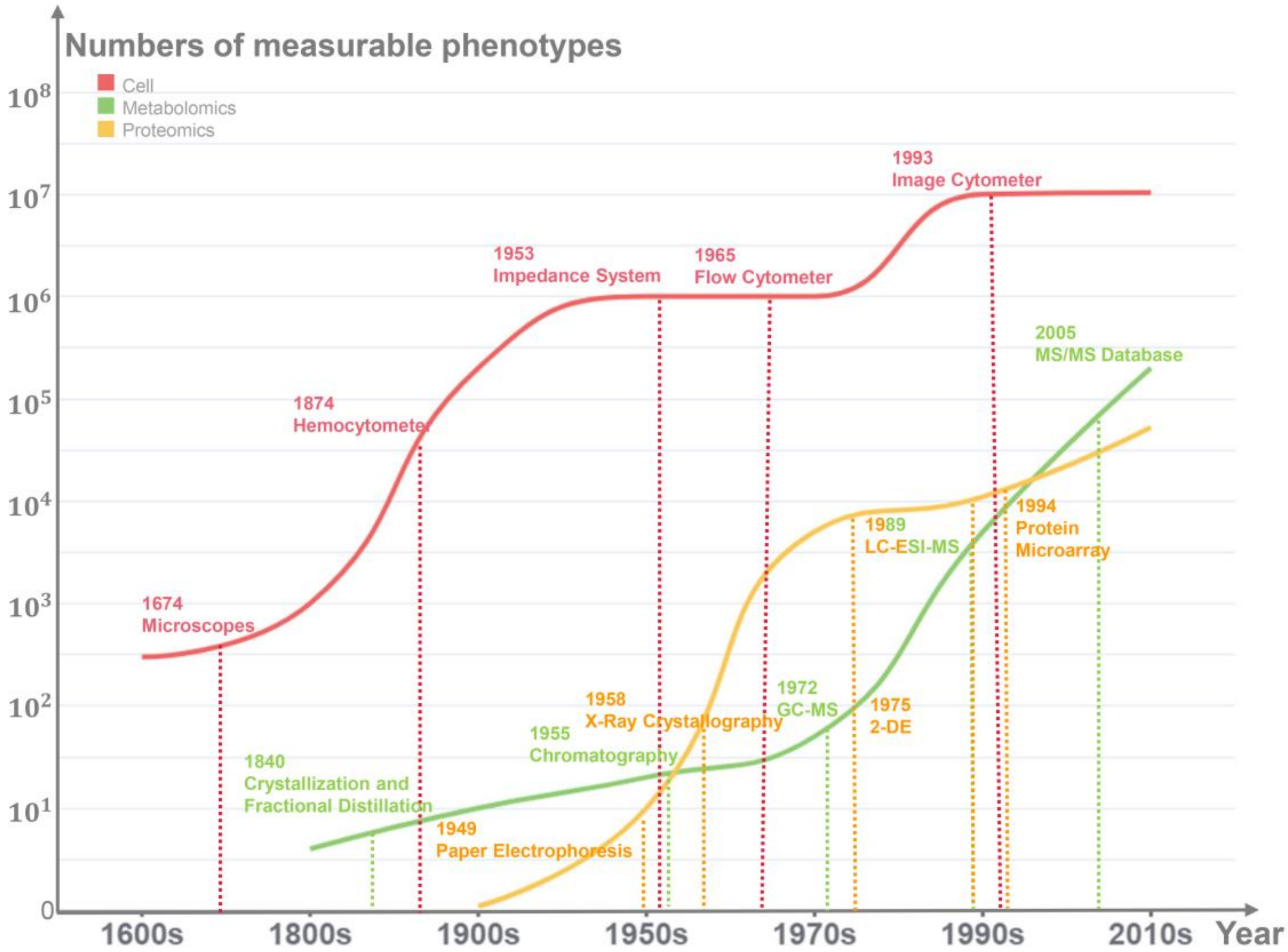

Figure S1. Snapshot of significant progress in human deep phenotype measurement

This chart illustrates the impact of evolving phenotype measurement techniques on deep phenotype measurement, using cell, proteomics, and metabolomics as examples. 2-DE, Two-Dimensional Electrophoresis; LC-ESI-MS, Liquid Chromatography-Electrospray Ionization-Mass Spectrometry; GC-MS, Gas Chromatography-Mass Spectrometry; MS/MS Database, Tandem Mass Spectrometry Database.

References

1. Wollman, A.J.M., Nudd, R., Hedlund, E.G., and Leake, M.C. (2015). From Animaculum to single molecules: 300 years of the light microscope. Open Biol. *5*, 150019. https://doi.org/10.1098/rsob.150019.
2. Vembadi, A., Menachery, A., and Qasaimeh, M.A. (2019). Cell Cytometry: Review and Perspective on Biotechnological Advances. Front. Bioeng. Biotechnol. *7*, 147. https://doi.org/10.3389/fbioe.2019.00147.
3. Simson, E. (2013). Wallace Coulter's life and his impact on the world. Int. J. Lab. Hematol. *35*, 230–236. https://doi.org/10.1111/ijlh.12069.
4. Larsen, U.D., Blankenstein, G., and Branebjerg, J. (1997). Microchip Coulter particle counter. In Proceedings of International Solid State Sensors and Actuators Conference (Transducers '97), pp. 1319–1322 vol.2. https://doi.org/10.1109/SENSOR.1997.635479.
5. Duranton, F., Jankowski, J., Więcek, A., and Argilés, À. (2016). On the discovery of UREA. Identification, synthesis and observations that let to establishing the first uraemic retention solute. G. Ital.

Nefrol. Organo Uff. Della Soc. Ital. Nefrol. *33 Suppl 66*, 33.S66.16.
6. Dalgliesh, C.E., Horning, E.C., Horning, M.G., Knox, K.L., and Yarger, K. (1966). A gas-liquid-chromatographic procedure for separating a wide range of metabolites occuring in urine or tissue extracts. Biochem. J. *101*, 792–810. https://doi.org/10.1042/bj1010792.
7. Fenn, J.B., Mann, M., Meng, C.K., Wong, S.F., and Whitehouse, C.M. (1989). Electrospray ionization for mass spectrometry of large biomolecules. Science *246*, 64–71. https://doi.org/10.1126/science.2675315.
8. Sanger, F. (1949). The terminal peptides of insulin. Biochem. J. *45*, 563. https://doi.org/10.1042/bj0450563.
9. Muirhead, H., and Perutz, M.F. (1963). Structure Of Hæemoglobin: A Three-Dimensional Fourier Synthesis of Reduced Human Haemoglobin at 5.5 Å Resolution. Nature *199*, 633–638. https://doi.org/10.1038/199633a0.
10. O'Farrell, P.H. (1975). High Resolution Two-Dimensional Electrophoresis of Proteins. J. Biol. Chem. *250*, 4007.
11. Karas, Michael., and Hillenkamp, Franz. (1988). Laser desorption ionization of proteins with molecular masses exceeding 10,000 daltons. Anal. Chem. *60*, 2299–2301. https://doi.org/10.1021/ac00171a028.

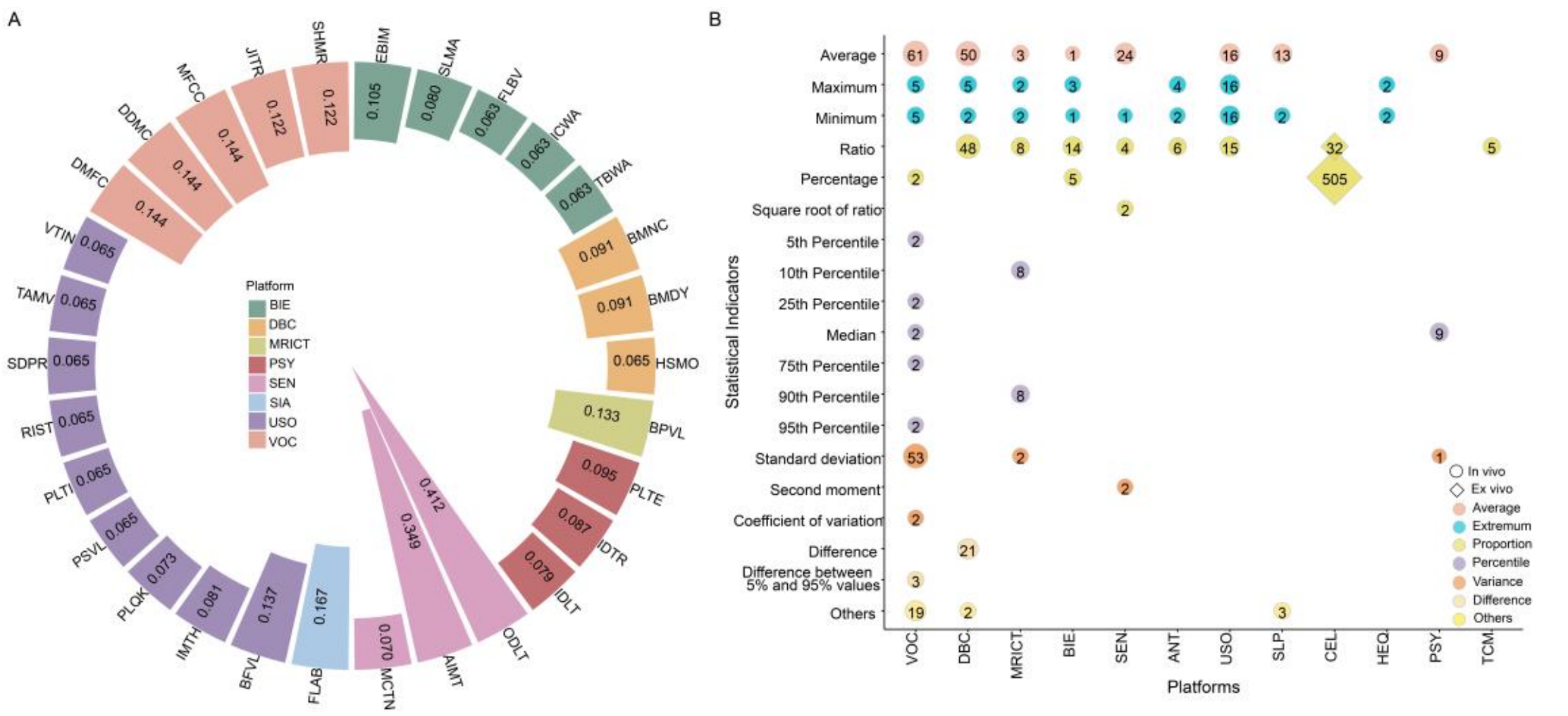

Figure S2. Dominant features and statistical indicator qualifiers across IHPP platforms

(A) Dominant features for fine-grained measurement. Features appearing more than eight times and representing over 6% of all PhenoDEs within a platform are classified as dominant. EBIM, Electrical bioimpedance; SLMA, Soft lean mass; FLBV, Extracellular water; ICWA, Intracellular water; TBWA, Total body water; BMNC, Bone mineral content; BMDY, Bone mineral density; HSMO, Hip shape model; BPVL, Brain parenchymal volume; PLTE, PAL Total Errors; IDTR, IED Total Trials; IDLT, IED Total Latency; ODLT, Optic disc layer thickness; AIMT, Acoustic immittance; MCTN, Macular thickness; FLAB, Facial L*a*b* values; BFVL, Blood flow velocity; IMTH, Intima-media thickness; PLQK, Plaque; PSVL, Peak systolic velocity; PLTI, Pulsatility index; RIST, Resistance index; SDPR,

Systolic to diastolic peak velocity ratio; TAMV, Time averaged maximum flow velocity; VTIN, Velocity time integral; DMFC, Delta Mel frequency cepstral coefficients; DDMC, Delta-delta Mel frequency cepstral coefficients; MFCC, Mel frequency cepstral coefficients; JITR, Jitter; SHMR, Shimmer.

(B) Qualifier statistical indicators (SI) across platforms. Derived phenotypes can be obtained through software-based extraction or computational derivation, and some platforms employ statistical indicators to describe measurement characteristics. Additional statistical indicators, including "Others," are listed in the controlled vocabulary available at https://github.com/BioMedBigDataCenter/PhenoAgents. VOC, Vocal; DBC, DXA body composition; MRICT, MRI & CT; BIE, Bioelectricity; SEN, Sensory; ANT, Anthropometry; USO, Ultrasonography; SLP, Sleep; CEL, Cell; HEQ, Health questionnaire; PSY, Psychology; TCM, Traditional Chinese Medicine.

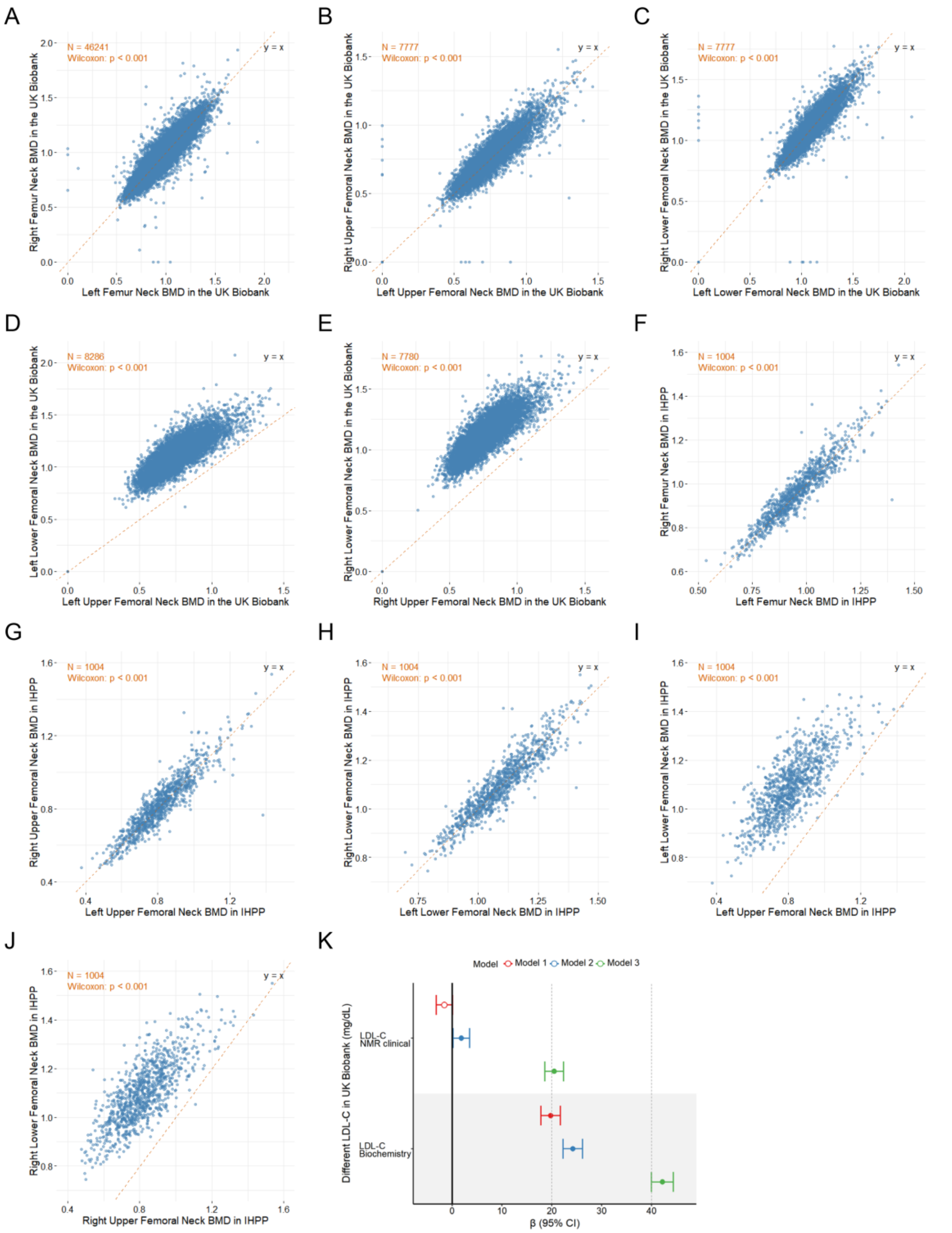

Figure S3. The impact of measurement discrepancies on statistical analysis results

(A-E) Directional differences in bone mineral density (BMD) measurement in UK Biobank. The unit of BMD is g/cm².

(F-J) Directional differences in BMD measurement in International Human Phenome Project (IHPP). The unit of BMD is g/cm².

(K) Association between waist-to-hip ratio (WHR) and low-density lipoprotein cholesterol (LDL-C)

measured by different instruments in UK Biobank. In UK Biobank, LDL-C was measured using a high-throughput NMR-based metabolic biomarker profiling platform and a Beckman Coulter AU5800 (as a blood biochemistry biomarker). Data are presented as β and 95% confidence intervals (CI). Model 1: age and sex; Model 2: Model 1 plus race, Townsend deprivation index (TDI) and education; Model 3: Model 2 plus body mass index, hypertension and diabetes. Filled points represent statistically significant associations ($p < 0.05$), while hollow points represent non-significant associations.

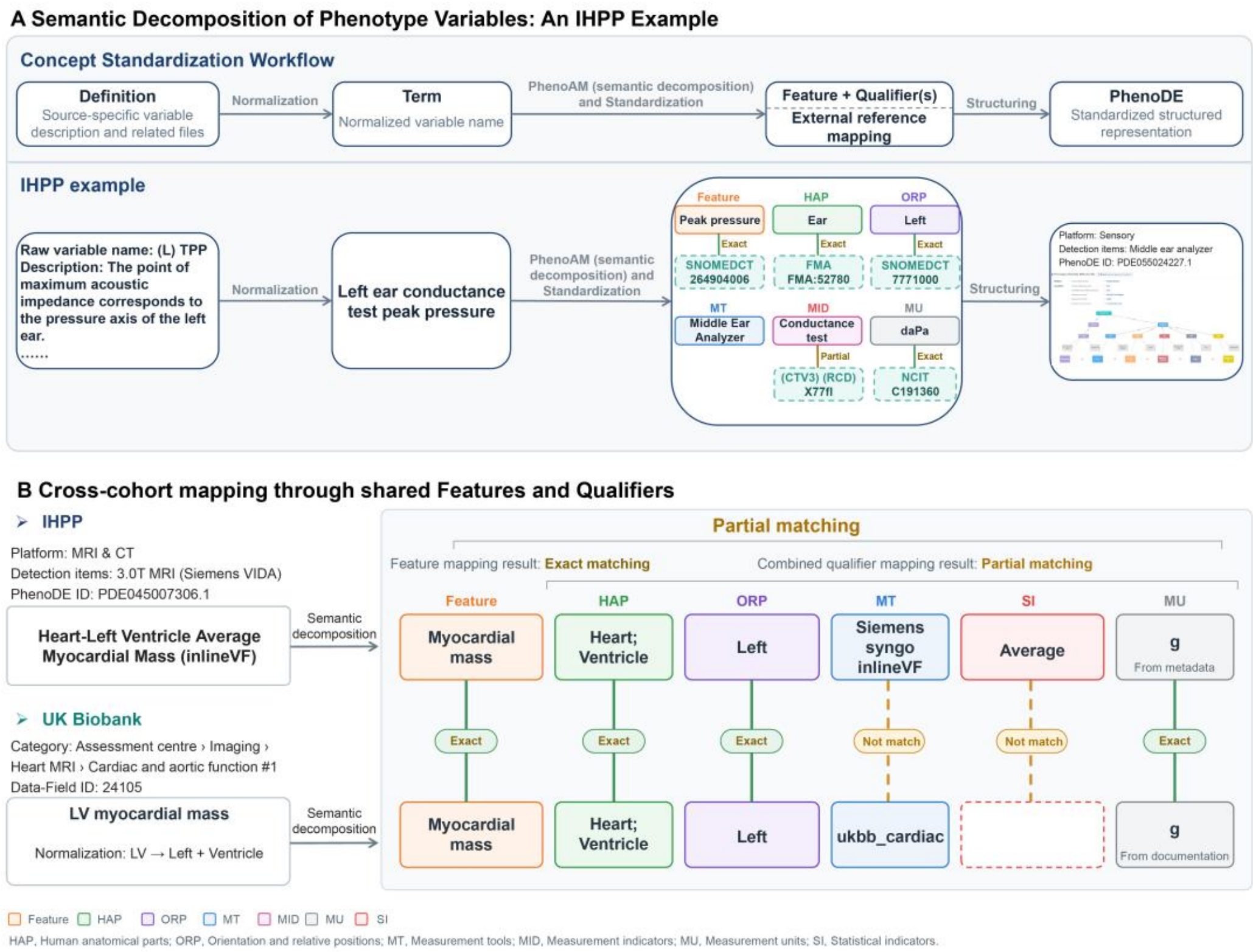

Figure S4. Worked examples of semantic decomposition, PhenoDE construction, and cross-cohort mapping.

(A) Semantic decomposition of an IHPP phenotype variable into a PhenoDE. The conceptual workflow comprises normalization of a source-specific variable definition into a phenotype term, PhenoAM semantic decomposition into one Feature and all applicable Qualifiers, mapping of these components to external reference resources, and subsequent structuring into a PhenoDE. The IHPP variable "(L) TPP" is shown as a worked example. Its raw variable name, source definition, and associated information were normalized to the term "Left ear conductance test peak pressure." PhenoAM decomposition identified "Peak pressure" as the Feature, with "Ear" as the human anatomical parts (HAP), "Left" as the orientation

and relative positions (ORP), “Middle Ear Analyzer” as the measurement tools (MT), “Conductance test” as the measurement indicators (MID), and “daPa” as the measurement units (MU). The Feature “Peak pressure” was mapped to the SNOMED CT concept http://purl.bioontology.org/ontology/SNOMEDCT/264904006. Among the Qualifiers, “Ear” was mapped to FMA (http://purl.org/sig/ont/fma/fma52780), “Left” to SNOMED CT (http://purl.bioontology.org/ontology/SNOMEDCT/7771000), “Conductance test” to (CTV3) (RCD) (http://purl.bioontology.org/ontology/RCD/X77fl), and “daPa” to NCIT (http://ncicb.nci.nih.gov/xml/owl/EVS/Thesaurus.owl#C191360). The MT Qualifier “Middle Ear Analyzer” was not mapped to an external reference resource. These standardized components were structured into PhenoDE PDE055024227.1.

(B) Cross-cohort mapping through shared Features and Qualifiers. The IHPP variable “Heart-Left Ventricle Average Myocardial Mass (inlineVF)” and UK Biobank Field 24105, “LV myocardial mass,” were represented using the same PhenoAM representation and compared component by component. The UK Biobank abbreviation “LV” was anatomically normalized to “Left” and “Ventricle.” The two representations shared the Feature “Myocardial mass,” the HAP Qualifiers “Heart” and “Ventricle,” the ORP Qualifier “Left,” and the MU Qualifier “g.” Their MT Qualifiers differed, with “Siemens syngo inlineVF” in IHPP and “ukbb_cardiac” in UK Biobank. The SI Qualifier “Average” was represented only in the IHPP PhenoDE. This variable pair therefore showed an exact Feature match and a partial combined Qualifier match. Solid green lines indicate exact component matches, orange dashed lines indicate non-matching components, and an unfilled dashed box indicates a component not represented in the available source information. HAP, human anatomical parts; ORP, orientation and relative positions; MT, measurement tools; MID, measurement indicators; SI, statistical indicators; MU, measurement units.
