# Supplementary material for: Towards a Compositional Framework for Describing Human Phenotypes
Source: Adv Sci (Weinh). 2026 Sep 22:e77730. Online ahead of print. doi: 10.1002/advs.77730 (PMC13598380; doi:10.1002/advs.77730)
Supplement: Supplementary file 2 [file ADVS-9999-e77730-s002.pdf]

Supporting Information

Towards a Compositional Framework for Describing Human Phenotypes

Contents

Supplementary Methods and Results 1

Section 1. Mapping Statistics and Inter-rater Reliability 1

Section 2. PhenoAgents Evaluation 5

Section 3. Distribution Fitting in PhenoCurator 31

Supplementary Tables 33

Supplementary Notes 35

Note S1. Comparison of HPO, Phenopackets, NIH CDE, and PhenoAM/PhenoDE 35

Note S2. Anatomical classification of HAP qualifiers and associations 49

Note S3. Positioning PhenoAM and PhenoDE relative to SNOMED CT postcoordination 52

Note S4. Positioning PhenoAM and PhenoDE relative to established data-element, terminology, and clinical observation frameworks 55

Note S5. PhenoAM Annotation Rules 57

Supplementary Methods and Results
Section 1. Mapping Statistics and Inter-rater Reliability

Methods

Calculation of mapping rates

Mapping outcomes were classified as Equivalent, Expanded, or No corresponding mapping according to the predefined combinations of the feature mapping result and combined qualifier mapping result. Mapped PhenoDEs were defined as those classified as Equivalent or Expanded. Measurement Tool (MT) was excluded from the combined qualifier mapping result and from the mapping statistics.

Both unweighted macro-average mapping rates and pooled mapping coverage were calculated because the two statistics use different denominators. For each platform, the platform-specific mapping rate was calculated as the number of PhenoDEs classified as Equivalent or Expanded divided by the total number of PhenoDEs in that platform. The unweighted macro-average mapping rate was calculated as the arithmetic mean of the platform-specific mapping rates across the platforms included in the analysis, thereby assigning equal weight to each platform. Pooled mapping coverage was calculated as the total number of PhenoDEs classified as Equivalent or Expanded across the included platforms divided by the total number of PhenoDEs across the same platforms. Accordingly, the pooled statistic reflects differences in the number of PhenoDEs across platforms. Expanded, Equivalent, and No corresponding mapping percentages in pooled rows were calculated using the pooled denominator, whereas macro-average category percentages were calculated as the arithmetic means of the corresponding platform-specific percentages.

For phenotypes from the four omics platforms, the DE generally directly identified the biological entity being measured and was therefore represented as the feature without further semantic decomposition. In constructing the corresponding PhenoDE, measurement-context information, including sample type, measurement tool, measurement method, and other applicable conditions, was represented through the corresponding qualifiers. These features were mapped predominantly to dedicated domain databases.

Mapping statistics were calculated for both an 18-platform analysis set and the all-22-platform dataset. The 18-platform analysis set comprised all platforms other than the four omics platforms and therefore included Cell. The four omics platforms, Metabolome, Microbiome, Proteome, and Total RNA and small RNA, were not included in the original 18-platform analysis because their PhenoDEs were predominantly defined by biological features that could be mapped directly to dedicated domain databases, whereas measurement-context information was represented separately through qualifiers. The Cell platform was retained because cell features were mapped to the Cell Ontology and supporting literature rather than predominantly to dedicated molecular databases, and some cell phenotypes additionally use Statistical Indicator (SI) qualifiers to represent derived measures such as cell proportions. The all-22-platform analysis additionally included the four omics platforms to provide mapping statistics across the complete set of IHPP platforms.

Inter-rater reliability of mapping classification

To evaluate the reliability of the manual mapping classification, we performed an independent inter-rater reliability assessment using a category-enriched stratified validation sample. A total of 300 PhenoDEs were sampled from all 22 platforms using a fixed random seed and platform-coverage constraints. The validation sample comprised 266 unique feature mapping records and 180 unique non-MT qualifier mapping records. This sampling strategy ensured representation across all platforms and mapping categories and was designed to assess classification consistency rather than estimate population-level mapping proportions. For each sampled PhenoDE, the corresponding feature and non-MT qualifier mapping records were independently reviewed by two reviewers using separately prepared review tables. Each reviewer was blinded to the other reviewer's classifications and to the original CV mapping results. Each mapping record was classified as Exact match, Partial match, or No match according to the predefined mapping criteria. Reviewer-specific feature and qualifier classifications were then mapped back to the sampled PhenoDEs, and the same predefined combination rules were applied to derive the corresponding

PhenoDE-level Equivalent, Expanded, or No corresponding mapping classification. Inter-rater reliability was assessed using Cohen's kappa at both the component level (feature and qualifier mappings) and the PhenoDE level. Reviewer-derived PhenoDE-level classifications were also compared with the original CV-derived classifications to assess the reproducibility of the manual mapping classification.

Results

Table S1. Component-level inter-rater reliability

Level	Comparison	n	Concordant records	Concordance rate	Cohen's kappa (95% CI)
**Qualifier**	Reviewer 1 vs Reviewer 2	180	180	1.000	1.000 (1.000-1.000)
**Qualifier**	Reviewer 1 vs CV	180	179	0.994	0.980 (0.930-1.000)
**Qualifier**	Reviewer 2 vs CV	180	179	0.994	0.980 (0.930-1.000)
**Feature**	Reviewer 1 vs Reviewer 2	266	266	1.000	1.000 (1.000-1.000)
**Feature**	Reviewer 1 vs CV	266	266	1.000	1.000 (1.000-1.000)
**Feature**	Reviewer 2 vs CV	266	266	1.000	1.000 (1.000-1.000)

Note: The only qualifier-level discrepancy between the reviewer classifications and the original CV-derived classification involved ST12, “Scalp skin swab” (QMR0011). The original CV classified this mapping as Exact match by linking the qualifier to the FMA concept “Skin of scalp,” whereas both reviewers classified it as Partial match because the FMA concept represents an anatomical site rather than the sample or specimen context represented by the qualifier. Both reviewers assigned the same classification; therefore, this discrepancy did not affect inter-rater agreement.

Table S2. PhenoDE-level validation

Comparison	n	Concordant records	Concordance rate	Cohen's kappa (95% CI)
**Reviewer 1 vs Reviewer 2**	300	300	1.000	1.000 (1.000-1.000)
**Reviewer 1 vs original CV-derived PhenoDE category**	300	295	0.983	0.973 (0.948-0.995)
**Reviewer 2 vs original CV-derived PhenoDE category**	300	295	0.983	0.973 (0.948-0.995)

Note: The five discrepancies relative to the original CV-derived classifications were attributable to the single qualifier-level discrepancy described above, as the same qualifier was shared by five PhenoDEs. Under the predefined combination rules, the affected PhenoDEs were classified as Expanded rather than Equivalent. Because both categories were considered mapped, the reported mapping statistics were unchanged.

Table S3. Confusion matrix for PhenoDE-level classifications: Reviewer 1 (rows) versus Reviewer 2 (columns)

		**Reviewer 2**		
		Equivalent	Expanded	No corresponding mapping
**Reviewer 1**	Equivalent	145	0	0
	Expanded	0	105	0
	No corresponding mapping	0	0	50

Note: Reviewer 1 and Reviewer 2 assigned identical PhenoDE-level classifications for all 300 sampled PhenoDEs.

Table S4. Summary by scope and aggregation method

**Analysis**	**Aggregation**	**Platforms included**	**Total PhenoDEs in scope (reference only)**	**Expanded**	**Equivalent**	**No corresponding mapping**	**Mapped**
**Original refinement statistic**	18 platforms; unweighted macro average	All except Metabolome, Microbiome, Proteome, and Total RNA and small RNA; includes Cell	6,786	23.9%	72.4%	3.6%	96.4%
**18 platform pooled coverage**	18 platforms; pooled denominator	All except Metabolome, Microbiome, Proteome, and Total RNA and small RNA; includes Cell	6,786	2,461 (36.3%)	4,194 (61.8%)	131 (1.9%)	6,655/6,786 (98.1%)
**All platform macro average**	22 platforms; unweighted macro average	All 22 platforms	58,371	19.6%	77.4%	3.0%	97.0%
**All platform pooled coverage**	22 platforms; pooled denominator	All 22 platforms	58,371	2,461 (4.2%)	55,770 (95.5%)	140 (0.2%)	58,231/58,371 (99.8%)

Note: Mapped PhenoDEs were defined as those classified as Equivalent or Expanded. The unweighted macro-average mapping rate gives equal weight to each platform, whereas pooled mapping coverage is calculated across all included PhenoDEs and therefore reflects differences in platform size. Category percentages were calculated using the corresponding denominator for each aggregation approach. For macro-average rows, the total number of PhenoDEs is provided only to describe the analysis scope and is not used as the denominator for calculating the macro average.

Table S5. Platform-specific mapping counts and rates across all 22 platforms

Platform	In 18 platform set	Total	Expanded, n (%)	Equivalent, n (%)	No corresponding mapping, n (%)	Mapped, n	Mapped, %
**Anthropometry**	Yes	324	174 (53.7%)	150 (46.3%)	0 (0.0%)	324	100.00%
**Bioelectricity**	Yes	286	189 (66.1%)	97 (33.9%)	0 (0.0%)	286	100.00%
**Cell**	Yes	1,352	109 (8.1%)	1,243 (91.9%)	0 (0.0%)	1,352	100.00%
**DXA body composition**	Yes	615	199 (32.4%)	416 (67.6%)	0 (0.0%)	615	100.00%
**Eligibility Check**	Yes	13	1 (7.7%)	12 (92.3%)	0 (0.0%)	13	100.00%
**Energy metabolism**	Yes	5	0 (0.0%)	5 (100.0%)	0 (0.0%)	5	100.00%
**General physical examination with blood laboratory tests**	Yes	89	11 (12.4%)	78 (87.6%)	0 (0.0%)	89	100.00%
**Health questionnaire**	Yes	546	66 (12.1%)	430 (78.8%)	50 (9.2%)	496	90.84%
**MRI & CT**	Yes	1,049	166 (15.8%)	883 (84.2%)	0 (0.0%)	1,049	100.00%
**Metabolome**	No	2,030	0 (0.0%)	2,021 (99.6%)	9 (0.4%)	2,021	99.56%
**Microbiome**	No	1,255	0 (0.0%)	1,255 (100.0%)	0 (0.0%)	1,255	100.00%
**Plasma metabolic factors and cytokines**	Yes	18	0 (0.0%)	18 (100.0%)	0 (0.0%)	18	100.00%
**Proteome**	No	16,217	0 (0.0%)	16,217 (100.0%)	0 (0.0%)	16,217	100.00%
**Psychology**	Yes	126	16 (12.7%)	102 (81.0%)	8 (6.3%)	118	93.65%
**Pulmonary function**	Yes	16	0 (0.0%)	16 (100.0%)	0 (0.0%)	16	100.00%
**Sensory**	Yes	1,431	1,306 (91.3%)	125 (8.7%)	0 (0.0%)	1,431	100.00%
**Skin and its appendages**	Yes	60	16 (26.7%)	44 (73.3%)	0 (0.0%)	60	100.00%
**Sleep**	Yes	294	93 (31.6%)	189 (64.3%)	12 (4.1%)	282	95.92%
**Total RNA and small RNA**	No	32,083	0 (0.0%)	32,083 (100.0%)	0 (0.0%)	32,083	100.00%
**Traditional Chinese Medicine**	Yes	133	39 (29.3%)	33 (24.8%)	61 (45.9%)	72	54.14%
**Ultrasonography**	Yes	248	74 (29.8%)	174 (70.2%)	0 (0.0%)	248	100.00%
**Vocal**	Yes	181	2 (1.1%)	179 (98.9%)	0 (0.0%)	181	100.00%

Section 2. PhenoAgents Evaluation

Code and prompt availability: The model-specific prompts and code are available at https://github.com/BioMedBigDataCenter/PhenoAgents.

PhenoAssembler evaluation methods

An independent test set of 300 non-molecular phenotype records was sampled from PhenoDE after records used for prompt optimization had been excluded. The set comprised 143 in vivo measurements, 119 questionnaire measurements and 38 ex vivo measurements. Selection covered records with different feature classes, amounts of qualifier information and levels of feature-boundary or terminology complexity. The reference representations were manually curated and reviewed by the Human Phenome Project Technological Advisory Groups. Evaluation was conducted at the feature-class, feature-term and qualifier-term levels. Qualifiers comprised human anatomical parts (HAP), orientation and relative positions (ORP), measurement methods (MM), measurement conditions (MC), measurement indicators (MID), statistical indicators (SI), applicable subjects (AS) and other qualifiers (OT). Sample type, measurement tool, and measurement unit were supplied as contextual metadata and were not evaluated as target qualifier outputs.

The evaluation followed a two-model by two-prompt design. DeepSeek-V3 and GPT-4o were tested with a rule-enhanced prompt and a concise prompt, and each model-prompt condition was repeated independently three times on the same test set, giving 12 runs. The rule-enhanced prompt specified the PhenoAM definitions, feature-qualifier boundary rules, category constraints, and structured-output requirements. The concise prompt retained the task and output structure but included fewer explicit semantic rules. Temperature was set to 0.2. Before scoring, model outputs and reference terms were normalized for case, spacing, null expressions and selected synonymous expressions. Feature-class accuracy was the proportion of records with an exact class match. Feature-term exact-match rate was the proportion with identical normalized predicted and reference terms. The weighted feature-term score was the mean match weight across records, with weights of 1.00 for exact matches, 0.60 for substring matches, 0.40 for fuzzy matches and 0 for non-matches. Fuzzy matching required a Python difflib SequenceMatcher ratio of at least 0.80 and was applied only when exact and substring matching failed. Qualifier terms were evaluated as category-specific sets. Weighted precision, recall and F1 were calculated from weighted true-positive, false-positive and false-negative contributions within each category and then averaged across the eight categories. Records in which both the prediction and reference were empty for a category were excluded from that category's positive-only calculation.

Additional record-level statistical testing was performed to evaluate prompt, model, and repeated-run effects for PhenoAssembler. For prompt- and model-effect analyses, binary metrics (feature-class correctness and feature-term exact match) were reduced across the three repeated runs to a per-record majority outcome; McNemar tests and the corresponding proportions and contrasts were calculated from these same majority outcomes. For continuous or weighted metrics (feature-term weighted score and record-level qualifier weighted F1), the three repeated runs within each model-prompt condition were first averaged for each phenotype record and then compared across the same 300 records using paired sign-flip permutation tests. Repeated-run effects were assessed using Cochran's Q tests for binary metrics and Friedman tests with correction for tied ranks for continuous or weighted metrics. P values were adjusted within each testing family using the Benjamini-Hochberg procedure. The record-level qualifier weighted F1 used for these paired tests supported within-record comparisons; the positive-only category-level macro metrics are reported in Tables S6 and S7.

A targeted manual-review scoring-sensitivity analysis was conducted for run 2 of DeepSeek-V3 with the rule-enhanced prompt because its automatic metrics were close to the corresponding three-run means. Outputs classified automatically as exact matches were also manually inspected as a separate quality-control check. These checks were not entered individually in the review worksheet and were excluded from the formal sensitivity and inter-assessor analyses. The formal analyses covered the 401 items classified automatically as non-exact or inconsistent: 3 feature-class, 205 feature-term and 193 qualifier-term items. Two assessors independently classified each item as correct, partially correct or incorrect. Correct indicated semantic equivalence to, or acceptability against, the

reference; partially correct indicated a valid but incomplete or broader relationship. When the two labels agreed, correct, partially correct, and incorrect were assigned values of 1.0, 0.5 and 0, respectively, for metric recalculation. The 40 discordant items were not adjudicated and retained their prespecified automatic matching scores.

PhenoAssembler evaluation results

Comparison of the four model-prompt conditions showed feature-class accuracies of 0.9667-0.9911 and feature-term exact-match rates of 0.3056-0.4044. Rule enhancement produced the largest improvement in qualifier extraction. Weighted positive macro F1 increased from 0.3690 to 0.6082 for DeepSeek-V3 and from 0.2976 to 0.5181 for GPT-4o. With the rule-enhanced prompt, DeepSeek-V3 achieved the higher qualifier F1, whereas GPT-4o was slightly higher for feature-class accuracy and feature-term performance. Model differences were therefore task-specific rather than uniform. The small SDs indicate consistent performance across the three repeats; the lower feature-term exact-match rates were not primarily attributable to run-to-run variation. In the assessed representative run, feature-term exact-match rate increased from 0.3200 to 0.5200 and the weighted feature-term score increased from 0.4247 to 0.6803 after concordant assessor labels were incorporated. This difference indicates that exact and rule-based string matching did not capture some semantically acceptable term variants. Overall, explicit semantic rules improved structured qualifier extraction, whereas feature-term boundary and granularity decisions remained the principal source of disagreement with the curated reference.

Record-level paired tests showed that the rule-enhanced prompt improved record-level qualifier weighted F1 for both DeepSeek-V3 (mean difference, +0.0698; BH-adjusted $q<0.001$) and GPT-4o (+0.0976; $q<0.001$). Prompt effects on feature-term metrics were model dependent: GPT-4o showed a higher feature-term weighted score with the rule-enhanced prompt, whereas DeepSeek-V3 showed higher feature-term exact and weighted scores with the concise prompt. Model comparisons were likewise prompt- and metric-dependent. Across the three repeated runs, the observed run-level mean ranges were small, and no statistically detectable run effect remained after multiple-testing correction for any evaluated metric (Table S8). This lack of statistical significance does not establish equivalence among runs.

Table S6. PhenoAssembler performance across model and prompt conditions.

Model	Prompt condition	Feature-class accuracy	Feature-term exact-match rate	Feature-term weighted score	Qualifier weighted positive macro precision	Qualifier weighted positive macro recall	Qualifier weighted positive macro F1
DeepSeek-V3	Rule-enhanced prompt	0.9889 ± 0.0019	0.3256 ± 0.0051	0.4278 ± 0.0030	0.5964 ± 0.0111	0.6620 ± 0.0012	0.6082 ± 0.0074
GPT-4o	Rule-enhanced prompt	0.9911 ± 0.0038	0.3345 ± 0.0107	0.4498 ± 0.0129	0.4560 ± 0.0023	0.6482 ± 0.0027	0.5181 ± 0.0016
DeepSeek-V3	Concise prompt	0.9667 ± 0.0000	0.4044 ± 0.0051	0.4838 ± 0.0057	0.3824 ± 0.0131	0.3751 ± 0.0078	0.3690 ± 0.0096
GPT-4o	Concise prompt	0.9900 ± 0.0000	0.3056 ± 0.0107	0.4064 ± 0.0054	0.2759 ± 0.0095	0.3756 ± 0.0004	0.2976 ± 0.0061

Note: Values are mean ± SD across three runs. Feature-term exact-match rate reports exact agreement between normalized predicted and reference terms. The weighted feature-term score assigns partial credit according to the prespecified term-matching weights described above. Qualifier metrics are positive-only macro averages across the eight categories.

Table S7. Qualifier-category F1 across PhenoAssembler model and prompt conditions.

Model	Prompt condition	HAP weighted positive F1	ORP weighted positive F1	MM weighted positive F1	MC weighted positive F1	MID weighted positive F1	SI weighted positive F1	AS weighted positive F1	OT weighted positive F1
DeepSeek-V3	Rule-enhanced prompt	0.6499 ± 0.0148	0.9103 ± 0.0041	0.7922 ± 0.0186	0.7312 ± 0.0216	0.4154 ± 0.0276	0.5751 ± 0.0226	0.7917 ± 0.0301	0.0000 ± 0.0000
GPT-4o	Rule-enhanced prompt	0.6540 ± 0.0110	0.8920 ± 0.0146	0.6755 ± 0.0150	0.4425 ± 0.0083	0.3032 ± 0.0092	0.4523 ± 0.0216	0.7249 ± 0.0108	0.0000 ± 0.0000
DeepSeek-V3	Concise prompt	0.6506 ± 0.0174	0.8227 ± 0.0074	0.0939 ± 0.0044	0.5647 ± 0.0192	0.1529 ± 0.0261	0.3245 ± 0.0088	0.3429 ± 0.0194	0.0000 ± 0.0000
GPT-4o	Concise prompt	0.5297 ± 0.0145	0.8206 ± 0.0080	0.0470 ± 0.0064	0.3692 ± 0.0119	0.0818 ± 0.0097	0.1367 ± 0.0110	0.3960 ± 0.0542	0.0000 ± 0.0000

Note: HAP, human anatomical parts; ORP, orientation and relative positions; MM, measurement methods; MC, measurement conditions; MID, measurement indicators; SI, statistical indicators; AS, applicable subjects; OT, other qualifiers. OT was represented in the test set and reference annotations; the zero values indicate no weighted true-positive contribution under the prespecified matching rules.

Table S8. Record-level statistical tests of model, prompt, and repeated-run effects for PhenoAssembler.

Analysis dimension	Fixed condition/comparison	Metric	Summary contrast	P value	BH-adjusted q	Interpretation
Prompt effect	DeepSeek-V3: rule-enhanced vs concise	Feature-class correctness	+0.0233	0.065	0.087	Not significant after adjustment
Prompt effect	DeepSeek-V3: rule-enhanced vs concise	Feature-term exact match	-0.0800	<0.001	<0.001	Concise higher
Prompt effect	DeepSeek-V3: rule-enhanced vs concise	Feature-term weighted score	-0.0540	<0.001	0.001	Concise higher
Prompt effect	DeepSeek-V3: rule-enhanced vs concise	Record-level qualifier weighted F1	+0.0698	<0.001	<0.001	Rule-enhanced higher
Prompt effect	GPT-4o: rule-enhanced vs concise	Feature-class correctness	+0.0033	1.000	1.000	Not significant after adjustment
Prompt effect	GPT-4o: rule-enhanced vs concise	Feature-term exact match	+0.0267	0.215	0.246	Not significant after adjustment
Prompt effect	GPT-4o: rule-enhanced vs concise	Feature-term weighted score	+0.0440	0.005	0.008	Rule-enhanced higher

Analysis dimension	Fixed condition/comparison	Metric	Summary contrast	P value	BH-adjusted q	Interpretation
Prompt effect	GPT-4o: rule-enhanced vs concise	Record-level qualifier weighted F1	+0.0976	<0.001	<0.001	Rule-enhanced higher
Model effect	Rule-enhanced prompt: DeepSeek-V3 vs GPT-4o	Feature-class correctness	-0.0033	1.000	1.000	Not significant after adjustment
Model effect	Rule-enhanced prompt: DeepSeek-V3 vs GPT-4o	Feature-term exact match	-0.0033	1.000	1.000	Not significant after adjustment
Model effect	Rule-enhanced prompt: DeepSeek-V3 vs GPT-4o	Feature-term weighted score	-0.0232	0.172	0.229	Not significant after adjustment
Model effect	Rule-enhanced prompt: DeepSeek-V3 vs GPT-4o	Record-level qualifier weighted F1	+0.0204	0.005	0.009	DeepSeek-V3 higher
Model effect	Concise prompt: DeepSeek-V3 vs GPT-4o	Feature-class correctness	-0.0233	0.092	0.148	Not significant after adjustment
Model effect	Concise prompt: DeepSeek-V3 vs GPT-4o	Feature-term exact match	+0.1033	<0.001	<0.001	DeepSeek-V3 higher
Model effect	Concise prompt: DeepSeek-V3 vs GPT-4o	Feature-term weighted score	+0.0748	<0.001	<0.001	DeepSeek-V3 higher
Model effect	Concise prompt: DeepSeek-V3 vs GPT-4o	Record-level qualifier weighted F1	+0.0482	<0.001	<0.001	DeepSeek-V3 higher
Repeated-run effect	DeepSeek-V3, rule-enhanced prompt	Feature-class correctness	Max-min run mean=0.0033	0.368	0.736	No statistically detectable run effect
Repeated-run effect	DeepSeek-V3, rule-enhanced prompt	Feature-term exact match	Max-min run mean=0.0100	0.756	0.756	No statistically detectable run effect
Repeated-run effect	DeepSeek-V3, rule-enhanced prompt	Feature-term weighted score	Max-min run mean=0.0057	0.478	0.726	No statistically detectable run effect
Repeated-run effect	DeepSeek-V3, rule-enhanced prompt	Record-level qualifier weighted F1	Max-min run mean=0.0034	0.180	0.360	No statistically detectable run effect

Analysis dimension	Fixed condition/comparison	Metric	Summary contrast	P value	BH-adjusted q	Interpretation
Repeated-run effect	GPT-4o, rule-enhanced prompt	Feature-class correctness	Max-min run mean=0.0067	0.135	0.541	No statistically detectable run effect
Repeated-run effect	GPT-4o, rule-enhanced prompt	Feature-term exact match	Max-min run mean=0.0200	0.144	0.424	No statistically detectable run effect
Repeated-run effect	GPT-4o, rule-enhanced prompt	Feature-term weighted score	Max-min run mean=0.0262	0.066	0.265	No statistically detectable run effect
Repeated-run effect	GPT-4o, rule-enhanced prompt	Record-level qualifier weighted F1	Max-min run mean=0.0018	0.802	0.807	No statistically detectable run effect
Repeated-run effect	DeepSeek-V3, concise prompt	Feature-class correctness	Max-min run mean=0.0000	1.000	1.000	No statistically detectable run effect
Repeated-run effect	DeepSeek-V3, concise prompt	Feature-term exact match	Max-min run mean=0.0100	0.678	0.756	No statistically detectable run effect
Repeated-run effect	DeepSeek-V3, concise prompt	Feature-term weighted score	Max-min run mean=0.0113	0.726	0.726	No statistically detectable run effect
Repeated-run effect	DeepSeek-V3, concise prompt	Record-level qualifier weighted F1	Max-min run mean=0.0099	0.056	0.224	No statistically detectable run effect
Repeated-run effect	GPT-4o, concise prompt	Feature-class correctness	Max-min run mean=0.0000	1.000	1.000	No statistically detectable run effect
Repeated-run effect	GPT-4o, concise prompt	Feature-term exact match	Max-min run mean=0.0200	0.212	0.424	No statistically detectable run effect
Repeated-run effect	GPT-4o, concise prompt	Feature-term weighted score	Max-min run mean=0.0103	0.620	0.726	No statistically detectable run effect
Repeated-run effect	GPT-4o, concise prompt	Record-level qualifier weighted F1	Max-min run mean=0.0010	0.807	0.807	No statistically detectable run effect

Note: For binary prompt and model comparisons, summary contrasts are differences between proportions calculated from the same per-record majority outcomes used in McNemar tests. For continuous or weighted prompt and model comparisons, contrasts are level A minus level B after averaging the three runs within each record. For repeated-run analyses, the summary contrast is the range of the three run means. Binary metrics were assessed with McNemar or Cochran's Q tests, and continuous or weighted metrics were assessed with paired sign-flip permutation tests or tie-corrected Friedman tests, as applicable. BH, Benjamini-Hochberg. A non-significant repeated-run test indicates that no run effect was statistically detected; it does not demonstrate equivalence among runs.

Table S9. Inter-assessor agreement in the targeted PhenoAssembler review.

**Overall reviewer-label cross-tabulation (n = 401)**				
Assessor 1 label	Assessor 2: Correct	Assessor 2: Partially correct	Assessor 2: Incorrect	**Row total**
Correct	76	11	3	90
Partially correct	0	121	24	145
Incorrect	1	1	164	166

Column total	77	133	191	401
		**Agreement by review target**		
**Review target**	**n**	**Concordant, n**	**Exact agreement**	**Cohen's kappa**
Overall	401	361	0.9002	0.8441
Feature class	3	3	1.0000	Not estimable
Feature term	205	178	0.8683	0.7925
Qualifier term	193	180	0.9326	0.8563

Note: Rows show Assessor 1 labels and columns show Assessor 2 labels from the initial independent assessments. Cohen's kappa was not estimable for the feature-class subset because the labels had no variation. The 40 discordant items retained their prespecified automatic matching scores and were not consensus-adjudicated.

Table S10. PhenoAssembler metrics before and after targeted manual review.

Metric	Automatic matching score before review	Score after incorporating concordant dual-assessor labels	Absolute change
Feature-class accuracy	0.9900	0.9900	0.0000
Feature-term exact-match rate	0.3200	0.5200	+0.2000
Feature-term weighted score	0.4247	0.6803	+0.2557
Qualifier weighted positive macro precision	0.5957	0.6190	+0.0233
Qualifier weighted positive macro recall	0.6627	0.6823	+0.0196
Qualifier weighted positive macro F1	0.6087	0.6293	+0.0207

Note: The pre-review score was obtained using the prespecified normalized string- and set-matching procedure for run 2 of DeepSeek-V3 with the rule-enhanced prompt. For separately recorded non-exact or inconsistent items, the assessor-derived value replaced the automatic matching score only when both assessors assigned the same label; discordant items retained the automatic score. Outputs classified automatically as exact matches were inspected separately but were not individually entered in the review worksheet or included in the formal sensitivity and inter-assessor analyses.

PhenoMapper evaluation methods

The fixed evaluation set comprised 200 reference mappings that were manually curated and expert-reviewed by the Human Phenome Project Technological Advisory Groups before PhenoMapper was run. It contained 80 feature terms and 120 qualifier terms. Each case included the input term, item type, classification metadata, platform information, target resource, standard identifier or link, and reference label. The curated reference mappings were withheld from candidate generation and used only for evaluation. Selection covered different phenotype categories, resource types, and mapping scenarios rather than reproducing their natural frequencies.

DeepSeek-V3 and GPT-4o were evaluated with detailed and concise prompts. The detailed prompt included category definitions, examples, candidate-resource selection rules, and ranking principles. The concise prompt retained the same category set, output schema and core ranking rules but reduced the definitions and examples. Each model-prompt condition was repeated independently three times with separate output and cache directories, a temperature of 0.2 and six

parallel workers. Candidate records returned by the configured resources were standardized, deduplicated and ranked using category-specific rules. For non-molecular BioPortal candidates, ranking combined model-assisted reranking, ontology priorities and a lexical-match fallback. Up to 10 candidates were retained for each case. Reference-standard recall was the proportion of cases with at least one candidate that matched the curated reference. Matching used identifiers, links or Internationalized Resource Identifiers (IRIs), normalized labels or synonyms, and PubMed or DOI evidence where applicable. Top-k hit rates described the position of the matching candidate in the ranked list. Model-generated semantic judgements and confidence scores were treated as auxiliary outputs.

For formal statistical analysis, reference-standard recall was treated as the primary endpoint. For each case, binary reference-match status was averaged across the three independent repeats within a condition, so repeated outputs from the same case were not treated as independent observations. Four planned model and prompt contrasts were assessed using two-sided paired random-sign permutation tests (500,000 permutations), with Holm adjustment across the four P values. Effect estimates are percentage-point differences with percentile 95% confidence intervals from 200,000 paired bootstrap resamples. Within each model-prompt condition, repeat-run stability of the binary reference-match outcome was assessed using Cochran's Q test across the three paired runs (df = 2). Top-k hit rates, matching-evidence counts and confidence analyses remained descriptive.

For the benchmark, gene terms were queried against HGNC, NCBI Gene, Ensembl and miRBase where applicable. Protein terms were queried against UniProt and the OLS Protein Ontology. Metabolite terms used PubChem, LIPID MAPS, RefMet, MetaboAnalyst and CTS-Lite; microbial terms used NCBI Taxonomy and OLS NCBITaxon. Cell terms were queried through BioPortal. Non-molecular terms used BioPortal with route-specific ontology priorities, with PubMed as a fallback when no candidate or adequate label match was returned. These resources and priorities were fixed for the benchmark. The released code permits modification or extension of the API configuration.

PhenoMapper evaluation results

GPT-4o achieved higher reference-standard recall than DeepSeek-V3 under both prompt conditions. With the detailed prompt, mean recall was 72.5% for GPT-4o and 65.8% for DeepSeek-V3; with the concise prompt, the corresponding values were 71.0% and 62.3%. Reducing the detailed prompt had a larger effect on the DeepSeek-V3 Top-10 hit rate (54.0% to 43.5%) than on GPT-4o (64.2% to 63.0%). These differences recurred across three runs with small SDs. The GPT-4o advantage in this fixed-reference retrieval task was therefore more consistent with a model-related difference than with random run variation. The larger prompt effect for DeepSeek-V3 was consistent with a model-dependent prompt effect: explicit definitions and ranking guidance benefited this model more strongly. Molecular-term recall exceeded non-molecular-term recall in every condition. This difference is consistent with the greater standardization of molecular identifiers and the broader semantic variation and ontology-coverage limitations of non-molecular terms. The results support PhenoMapper as a ranked-candidate retrieval aid rather than an autonomous mapper. Future models, terminology resources and task-specific prompting may improve recall, but this remains to be tested.

Formal paired case-level analyses confirmed that GPT-4o achieved higher reference-standard recall than DeepSeek-V3 under both prompts: detailed prompt, difference 6.7 percentage points (95% CI, 3.5 to 10.2; Holm-adjusted P = 0.000186); concise prompt, difference 8.7 percentage points (95% CI, 4.7 to 12.8; Holm-adjusted P = 0.000136). For prompt effects, the detailed prompt improved DeepSeek-V3 recall by 3.5 percentage points (95% CI, 0.8 to 6.5; Holm-adjusted P = 0.043640), whereas the 1.5-percentage-point difference for GPT-4o was not statistically detectable (95% CI, -0.2 to 3.5; Holm-adjusted P = 0.157106). No statistically detectable difference in reference-standard recall was observed across the three repeats within any condition (DeepSeek-V3 detailed, Cochran's Q = 2.364, P = 0.306721; DeepSeek-V3 concise, Q = 1.600, P = 0.449329; GPT-4o detailed, Q = 0, P = 1.000000; GPT-4o concise, Q = 0, P = 1.000000). These tests support repeatability in this fixed benchmark while showing that absolute performance was model- and prompt-dependent; a non-significant repeat-run test does not establish statistical equivalence.

Table S11. PhenoMapper performance across model and prompt conditions.

Model	Prompt condition	Reference-matched cases, mean n	Reference-standard recall	Top-1 hit rate	Top-10 hit rate	Model-reported confidence, mean score	No-candidate cases, mean n
DeepSeek-V3	Detailed prompt	131.7 ± 2.1	65.8% ± 1.0%	33.5% ± 1.7%	54.0% ± 1.7%	79.2 ± 0.4	0.0 ± 0.0
GPT-4o	Detailed prompt	145.0 ± 0.0	72.5% ± 0.0%	34.3% ± 0.6%	64.2% ± 1.2%	78.5 ± 0.4	0.0 ± 0.0
DeepSeek-V3	Concise prompt	124.7 ± 1.2	62.3% ± 0.6%	23.3% ± 1.4%	43.5% ± 1.0%	78.1 ± 0.6	0.7 ± 0.6
GPT-4o	Concise prompt	142.0 ± 0.0	71.0% ± 0.0%	36.2% ± 0.3%	63.0% ± 0.5%	79.1 ± 0.9	1.0 ± 0.0

Note: All values are mean ± SD across three runs. A reference-standard match required at least one prespecified evidence field to agree with the manually curated reference; the count is out of 200 cases. Model-reported confidence is an auxiliary self-assessment and not a calibrated probability.

Table S12. Formal paired comparisons of PhenoMapper reference-standard recall.

Comparison	Reference-standard recall, %	Difference, percentage points (95% CI)	Holm-adjusted P value	Interpretation
GPT-4o versus DeepSeek-V3, detailed prompt	72.5 versus 65.8	+6.7 (3.5 to 10.2)	0.000186	Statistically significant
GPT-4o versus DeepSeek-V3, concise prompt	71.0 versus 62.3	+8.7 (4.7 to 12.8)	0.000136	Statistically significant
Detailed versus concise prompt, DeepSeek-V3	65.8 versus 62.3	+3.5 (0.8 to 6.5)	0.043640	Statistically significant
Detailed versus concise prompt, GPT-4o	72.5 versus 71.0	+1.5 (-0.2 to 3.5)	0.157106	Not statistically significant

Note: Reference-standard recall was the primary endpoint. Each comparison used the case-level mean binary reference-match status across the three repeats. Two-sided paired random-sign permutation tests used 500,000 permutations. The four P values were adjusted using Holm's method. Percentile 95% confidence intervals were obtained from 200,000 paired bootstrap resamples.

Table S13. Statistical assessment of repeat-run stability for PhenoMapper reference-standard recall.

Model	Prompt	Reference-matched cases, runs 1/2/3	Cochran's Q (df = 2)	P value	Interpretation
DeepSeek-V3	Detailed	134/130/131	2.364	0.306721	No statistically detectable difference
DeepSeek-V3	Concise	124/124/126	1.600	0.449329	No statistically detectable difference
GPT-4o	Detailed	145/145/145	0.000	1.000000	No statistically detectable difference

GPT-4o	Concise	142/142/142	0.000	1.000000	No statistically detectable difference

Note: Cochran's Q tested the three paired binary reference-match outcomes across the same 200 cases within each condition. A non-significant result indicates that no statistically detectable run-to-run difference was observed for the primary endpoint; it does not establish statistical equivalence.

Table S14. PhenoMapper Top-k hit rates and matching evidence.

Model	Prompt condition	Top-1 hit rate	Top-3 hit rate	Top-5 hit rate	Top-10 hit rate	Identifier matches, mean n	Label matches, mean n	Synonym matches, mean n	Link/IRI matches, mean n
DeepSeek-V3	Detailed prompt	33.5% ± 1.7%	42.7% ± 1.9%	46.7% ± 1.8%	54.0% ± 1.7%	108.0 ± 3.5	88.7 ± 0.6	57.3 ± 0.6	71.0 ± 3.5
GPT-4o	Detailed prompt	34.3% ± 0.6%	50.7% ± 1.8%	56.5% ± 0.5%	64.2% ± 1.2%	128.3 ± 2.3	97.0 ± 1.0	74.0 ± 1.0	91.3 ± 2.3
DeepSeek-V3	Concise prompt	23.3% ± 1.4%	33.3% ± 0.6%	36.5% ± 1.3%	43.5% ± 1.0%	87.0 ± 2.0	82.7 ± 1.5	48.0 ± 2.0	50.0 ± 2.0
GPT-4o	Concise prompt	36.2% ± 0.3%	50.8% ± 0.3%	55.8% ± 0.6%	63.0% ± 0.5%	126.0 ± 1.0	95.3 ± 0.6	72.7 ± 1.5	89.3 ± 0.6

Note: All values are mean ± SD across three runs. Top-k values are case-level hit rates. Evidence counts are not mutually exclusive because a single case can match through more than one field. IRI, Internationalized Resource Identifier.

Table S15. Stratified reference-standard recall in PhenoMapper.

Model	Prompt condition	Molecular-term recall	Non-molecular-term recall	Confidence 0-19: recall (mean n)	Confidence 60-79: recall (mean n)	Confidence 80-89: recall (mean n)	Confidence 90-100: recall (mean n)
DeepSeek-V3	Detailed prompt	91.2%	59.2%	48.2% (9.7)	33.8% (86.7)	50.0% (0.7; sparse)	95.1% (101.3)
GPT-4o	Detailed prompt	94.9%	67.1%	44.6% (16.3)	44.4% (63.0)	Not estimable (0)	92.7% (114.3)
DeepSeek-V3	Concise prompt	83.2%	56.2%	38.1% (9.7)	35.7% (95.3)	Not estimable (0)	92.5% (93.3)
GPT-4o	Concise prompt	86.4%	66.7%	38.8% (16.3)	38.7% (60.3)	Not estimable (0)	93.0% (118.7)

Note: Molecular and non-molecular strata reflect PhenoMapper's category assignment for each run and were examined descriptively; they were not independently adjudicated performance strata. Confidence-stratum entries show recall followed by the mean number of cases per run. No cases received confidence 20-59. The confidence 80-89 interval was empty in three groups and contained fewer than one case per run on average in the remaining group; it should not be interpreted. Confidence analyses are descriptive and do not establish calibration.

PhenoCurator evaluation framework and shared workflow

PhenoCurator was evaluated using a simulation-based benchmark and a separate NHANES real-data analysis. The simulation-based benchmark measured quality-control candidate detection against predefined reference labels across model, prompt, input-information, and dataset conditions. The NHANES analysis evaluated candidate support, inter-assessor agreement and repeat-run stability in publicly available data, for which exhaustive annotation of every variable-value pair was not feasible. In both analyses, PhenoCurator processed each variable independently. Values were read as strings, unique observed values were extracted, and the relevant metadata, quality-control instructions, and structured-output schema were supplied to the model. High-cardinality variables were processed in batches, with the structured result from the preceding batch provided as context for the next batch. Outputs were parsed from JSON, cleaned of control characters, checked against the required schema and observed values, and regenerated when validation failed. Temperature was set to 0.2.

Distribution fitting and model selection

PhenoCurator profiles quantitative variables by fitting predefined parametric distributions selected according to variable type. This section specifies data preparation, candidate-model applicability, fitting, goodness-of-fit diagnostics, model selection, and reported output fields.

Each quantitative variable was represented as one row of the integrated Bigtable, with sample-level measurements stored across columns. Values that could not be converted to finite numeric values were excluded before fitting. Continuous variables with fewer than two non-missing observations or constant values were not assigned a parametric distribution. Discrete count variables required at least three non-missing observations and finite, nonnegative integer values. Variables that did not meet these conditions were retained in the output with the corresponding final status.

Normal and Cauchy distributions were evaluated for finite continuous observations. Lognormal distributions were evaluated only when all observations were strictly positive, and exponential distributions only when all observations were nonnegative. Poisson and negative binomial distributions were evaluated for discrete count variables. Model parameters were estimated by maximum likelihood in R version 4.3.1.

For continuous variables, the Kolmogorov-Smirnov statistic and its nominal p value were used as exploratory goodness-of-fit diagnostics because the distribution parameters were estimated from the same observations. For discrete count variables, a chi-square goodness-of-fit test was calculated after categories with low expected counts had been pooled where feasible. Candidate distributions with a goodness-of-fit p value greater than 0.05 were retained, and the retained model with the lowest AIC was selected. BIC was reported as a supplementary comparative measure and was not used for selection. Variables for which no candidate satisfied the screening criterion were recorded as having no adequate parametric fit. When a valid chi-square assessment could not be calculated, the discrete-variable result was recorded as goodness-of-fit test not applicable.

Qualitative variables were summarized using observed category frequencies and proportions and visualized using stacked bar charts. The distribution-fitting output retained the variable identifier, inferred variable type, candidate model, fit status, parameter estimates, Akaike Information Criterion (AIC), Bayesian Information Criterion (BIC), goodness-of-fit statistic and p value, selected distribution and final status, as applicable.

PhenoCurator simulation-based benchmark methods

The simulation-based benchmark used metadata for 200 representative non-molecular phenotype variables selected from PhenoDE. For each variable, 1,000 synthetic observations were generated according to variable type, prespecified plausible ranges or valid categories, producing a 200 × 1,000 observation matrix. No participant-level observations were used. Normal continuous values were sampled from truncated normal distributions bounded by prespecified plausible ranges. Integer scores were sampled within valid ranges, categorical values from valid enumerations, and time values between 06:00 and 23:00 in HH:MM format. Variable-specific anomaly templates used units, scale, phenotype meaning, and observed quality-control patterns to determine applicable anomaly mechanisms. The benchmark therefore combined phenotype metadata and empirically informed anomaly mechanisms with generated normal observations and predefined reference labels.

Three datasets were constructed using a progressive inclusion design. Dataset 1 (Explicit Anomaly Dataset) contained boundary-defined explicit anomalies, including impossible continuous values, missing-value markers, nonsensical strings, and categorical typographical errors (10,000 anomalous observations; 5%; 3,021 unique anomalous values). Dataset 2 (Format and Coding Anomaly Dataset) retained all Dataset 1 anomalies and their matrix positions and added format, coding and textual-expression anomalies, including continuous-format errors, numeric-entry errors, encoding corruption, categorical synonyms or variants, noisy valid categories and compressed multi-select values (20,000 anomalous observations; 10%; 3,968 unique anomalous values). Dataset 3 (Semantic-Context-Dependent Anomaly Dataset) retained the anomalies from the preceding datasets and added anomalies requiring interpretation of phenotype semantics, measurement units, valid ranges or domain knowledge, including potentially valid extreme values, unit mismatches, detection-limit or range artefacts, cross-field categories, off-ontology responses and time-related inconsistencies (30,000 anomalous observations; 15%; 7,098 unique anomalous values). Each dataset contained 200,000 observations. The nested design (Dataset 1 ⊆ Dataset 2 ⊆ Dataset 3) preserved the traceability of anomaly settings and matrix positions across datasets.

DeepSeek-V3 and GPT-4o were evaluated using a factorial design comprising two prompts, three input-information levels, and the three datasets. Prompt 1 specified baseline criteria for impossible values, missing or invalid markers, nonsensical strings, format errors, unit mismatch, detection-limit or range artefacts, off-enumeration categories and domain mismatch. Prompt 2 retained these criteria and added explicit rules for distinguishing candidate missing-value codes from candidate anomalous values and for interpreting encoding corruption, free-text numbers, synonymous or misspelled categories, noisy valid values and compressed multi-select responses. Both prompts instructed the model not to classify a value as anomalous solely because it was statistically extreme. Observed values were supplied under all input-information conditions. Name only included the variable name or identifier. Basic metadata included the name, variable class, description, data type, unit or valid range, and available enumerations when applicable. Enhanced metadata included the Basic metadata fields plus structured phenotype semantics generated by PhenoAM, including available feature and qualifier information such as anatomy, orientation, measurement tool, and unit. These conditions correspond to name, metadata1 and metadata3, respectively, in the evaluation scripts. Each of the 36 model-prompt-input-dataset conditions was run independently three times, yielding 108 runs.

At the value level, the union of predicted candidate missing-value codes and candidate anomalous values was compared with the set of unique predefined anomalous values. Because the universe of possible values was not enumerated, value-level performance was evaluated from true-positive, false-positive and false-negative counts using precision, recall and F1. At the observation-point level, predicted candidate values were mapped to the 200 × 1,000 observation matrix and compared with the predefined point-level labels. For each run, micro-averaged metrics were calculated from counts pooled across variables, and macro F1 was the mean of variable-level F1 values.

Inferential analyses were additionally conducted at the variable level to quantify model, prompt, and repeat-run effects. For each endpoint (value level and observation-point level) and metric (Micro precision, Micro recall and Micro F1), model and prompt contrasts were evaluated using paired cluster permutation tests with 10,000 permutations, treating the 200 variables as clusters and retaining the matched experimental strata. Model effects were tested overall and separately within each prompt; prompt effects were tested overall and separately within each model. Variable-level cluster bootstrap resampling with 5,000 replicates was used to obtain 95% confidence intervals. P values were adjusted by the Holm method within each endpoint-metric family. Repeat-run effects on variable-level F1 were assessed using paired Friedman tests across the three runs, both overall and within each model-prompt condition, followed by all pairwise paired sign-flip permutation tests (10,000 permutations). Holm adjustment was applied across the five omnibus subsets within each endpoint and across the three pairwise run comparisons within each endpoint-subset family.

PhenoCurator simulation-based benchmark results

Across input-information and dataset conditions, DeepSeek-V3 with Prompt 2 achieved the highest value-level Micro F1 (0.7345 ± 0.0757) and point-level Micro F1 (0.7090 ± 0.0256). At the value level, GPT-4o had higher precision but lower recall under both prompts. At the point level, GPT-4o had slightly higher precision with Prompt 1, whereas DeepSeek-V3 had slightly higher precision with Prompt 2; DeepSeek-V3 had higher recall and F1 at both levels under

both prompts. Prompt 2 modestly improved DeepSeek-V3 but reduced GPT-4o F1, indicating a model-dependent prompt effect rather than a uniform benefit. Dataset 2 yielded the lowest overall value-level F1 for both models, indicating lower performance for format, coding, and non-standard representation anomalies. Performance also differed across Dataset 1, Dataset 2 and Dataset 3; the datasets were defined by their anomaly content and nested inclusion structure rather than by a performance ranking. The reported SDs include variation across input-information levels and datasets as well as repeated runs.

The inferential analysis confirmed a model effect that was consistent across prompts but identified a model-dependent prompt effect. For value-level Micro F1, DeepSeek-V3 exceeded GPT-4o overall by 0.0320 (95% CI, 0.0195 to 0.0449; Holm-adjusted P = 0.0006), with differences of 0.0225 under Prompt 1 (P = 0.0030) and 0.0415 under Prompt 2 (P = 0.0006). The overall Prompt 2 minus Prompt 1 difference was not significant (-0.0008; P = 0.7273), because Prompt 2 modestly increased F1 for DeepSeek-V3 (0.0087; P = 0.0268) but decreased F1 for GPT-4o (-0.0102; P = 0.0006). Observation-point results showed the same model advantage, while the Prompt 2 effect on DeepSeek-V3 observation-point F1 was not significant after correction. No omnibus or pairwise repeat-run comparison remained significant after Holm correction. This absence of a detectable systematic run effect does not establish statistical equivalence among runs. Complete results for Micro precision, recall and F1, including all non-significant contrasts, are reported in Tables S19-S21.

Table S16. Value-level performance in the PhenoCurator simulation-based benchmark.

Prompt	Model	TP, mean ± SD	FP, mean ± SD	FN, mean ± SD	Micro precision	Micro recall	Micro F1	Macro F1
Prompt 1	DeepSeek-V3	3008.6 ± 1147.9	582.0 ± 519.8	1634.0 ± 582.0	0.8328 ± 0.1382	0.6474 ± 0.0382	0.7258 ± 0.0754	0.7145 ± 0.0784
Prompt 1	GPT-4o	2726.8 ± 864.9	439.0 ± 465.6	1915.9 ± 873.4	0.8585 ± 0.1403	0.6011 ± 0.0667	0.7032 ± 0.0801	0.6944 ± 0.0869
Prompt 2	DeepSeek-V3	3058.7 ± 1193.0	555.1 ± 524.0	1584.0 ± 538.3	0.8409 ± 0.1380	0.6561 ± 0.0361	0.7345 ± 0.0757	0.7213 ± 0.0776
Prompt 2	GPT-4o	2647.5 ± 816.5	421.3 ± 478.4	1995.2 ± 921.9	0.8618 ± 0.1468	0.5855 ± 0.0701	0.6930 ± 0.0834	0.6815 ± 0.0901

Note: Values are mean ± SD across 27 runs spanning three input-information levels, three datasets and three repeats within each prompt-model condition. The SD therefore captures variation across these experimental factors as well as repeated runs.

Table S17. Point-level performance in the PhenoCurator simulation-based benchmark.

Prompt	Model	TP, mean ± SD	FP, mean ± SD	FN, mean ± SD	Micro precision	Micro recall	Micro F1	Macro F1
Prompt 1	DeepSeek-V3	12336.1 ± 4689.7	2682.1 ± 435.5	7663.9 ± 3658.0	0.8009 ± 0.0804	0.6294 ± 0.0372	0.7008 ± 0.0175	0.7007 ± 0.0196
Prompt 1	GPT-4o	11158.3 ± 3532.4	2418.6 ± 609.7	8841.7 ± 4811.7	0.8050 ± 0.0890	0.5875 ± 0.0773	0.6700 ± 0.0217	0.6792 ± 0.0504
Prompt 2	DeepSeek-V3	12620.6 ± 4970.3	2597.0 ± 468.6	7379.4 ± 3389.2	0.8053 ± 0.0907	0.6399 ± 0.0292	0.7090 ± 0.0256	0.7088 ± 0.0134
Prompt 2	GPT-4o	10804.1 ± 3285.2	2497.2 ± 620.9	9195.9 ± 5057.4	0.7955 ± 0.0915	0.5719 ± 0.0817	0.6551 ± 0.0222	0.6634 ± 0.0551

Note: Values are mean ± SD across 27 runs spanning three input-information levels, three datasets and three repeats within each prompt-model condition.

Performance also varied with input-information level. DeepSeek-V3 achieved its highest value-level Micro F1 with Basic metadata (0.7395 ± 0.0737), whereas GPT-4o achieved its highest value with Enhanced metadata (0.7079 ± 0.0816). Additional metadata therefore did not improve every model-prompt condition uniformly. Together with the divergent Prompt 2 effects, this pattern indicates that performance depended on how model behaviour interacted with the prompt and input representation. The lower performance for format-and-coding errors in Dataset 2, together with the model-dependent effects of prompt and input representation, identifies representation handling and semantic interpretation as priorities for future model and prompt refinement. In its current form, PhenoCurator produces a structured and auditable candidate set that can focus manual quality-control review while retaining human adjudication for context-sensitive values.

Table S18. PhenoCurator performance across all simulation-based benchmark conditions.

Model	Input-information level	Prompt	Dataset	Value-level Micro precision	Value-level Micro recall	Value-level Micro F1	Point-level Micro precision	Point-level Micro recall	Point-level Micro F1
DeepSeek-V3	Name only	Prompt 1	Dataset 1	0.9043 ± 0.0278	0.6705 ± 0.0189	0.7700 ± 0.0200	0.6978 ± 0.0147	0.6658 ± 0.0196	0.6814 ± 0.0172
DeepSeek-V3	Name only	Prompt 1	Dataset 2	0.6454 ± 0.0012	0.5817 ± 0.0071	0.6119 ± 0.0036	0.8450 ± 0.0072	0.5932 ± 0.0049	0.6970 ± 0.0019
DeepSeek-V3	Name only	Prompt 1	Dataset 3	0.9772 ± 0.0010	0.6498 ± 0.0047	0.7806 ± 0.0037	0.8793 ± 0.0088	0.5864 ± 0.0062	0.7036 ± 0.0073
DeepSeek-V3	Name only	Prompt 2	Dataset 1	0.9097 ± 0.0151	0.6711 ± 0.0076	0.7724 ± 0.0098	0.6844 ± 0.0331	0.6654 ± 0.0089	0.6746 ± 0.0195
DeepSeek-V3	Name only	Prompt 2	Dataset 2	0.6498 ± 0.0169	0.6062 ± 0.0080	0.6271 ± 0.0051	0.8459 ± 0.0161	0.6193 ± 0.0089	0.7149 ± 0.0024
DeepSeek-V3	Name only	Prompt 2	Dataset 3	0.9732 ± 0.0094	0.6548 ± 0.0118	0.7829 ± 0.0082	0.8921 ± 0.0092	0.5955 ± 0.0150	0.7142 ± 0.0098
DeepSeek-V3	Basic metadata	Prompt 1	Dataset 1	0.9095 ± 0.0046	0.6849 ± 0.0022	0.7813 ± 0.0028	0.6885 ± 0.0051	0.6823 ± 0.0027	0.6854 ± 0.0017
DeepSeek-V3	Basic metadata	Prompt 1	Dataset 2	0.6656 ± 0.0051	0.6163 ± 0.0009	0.6400 ± 0.0019	0.8620 ± 0.0066	0.6293 ± 0.0015	0.7275 ± 0.0022
DeepSeek-V3	Basic metadata	Prompt 1	Dataset 3	0.9489 ± 0.0140	0.6708 ± 0.0047	0.7860 ± 0.0066	0.8678 ± 0.0225	0.6128 ± 0.0044	0.7183 ± 0.0105
DeepSeek-V3	Basic metadata	Prompt 2	Dataset 1	0.9025 ± 0.0437	0.6919 ± 0.0151	0.7830 ± 0.0217	0.6881 ± 0.0286	0.6843 ± 0.0140	0.6860 ± 0.0178
DeepSeek-V3	Basic metadata	Prompt 2	Dataset 2	0.6617 ± 0.0045	0.6181 ± 0.0047	0.6391 ± 0.0011	0.8447 ± 0.0202	0.6308 ± 0.0054	0.7221 ± 0.0054
DeepSeek-V3	Basic metadata	Prompt 2	Dataset 3	0.9732 ± 0.0028	0.6900 ± 0.0067	0.8075 ± 0.0047	0.8903 ± 0.0078	0.6383 ± 0.0030	0.7435 ± 0.0035
DeepSeek-V3	Enhanced metadata	Prompt 1	Dataset 1	0.8605 ± 0.0536	0.6884 ± 0.0134	0.7644 ± 0.0229	0.6945 ± 0.0089	0.6794 ± 0.0132	0.6868 ± 0.0035
DeepSeek-V3	Enhanced metadata	Prompt 1	Dataset 2	0.6342 ± 0.0209	0.6010 ± 0.0178	0.6171 ± 0.0184	0.8104 ± 0.0318	0.6146 ± 0.0209	0.6989 ± 0.0219

DeepSeek-V3	Enhanced metadata	Prompt 1	Dataset 3	0.9500 ± 0.0235	0.6631 ± 0.0143	0.7808 ± 0.0097	0.8624 ± 0.0054	0.6010 ± 0.0155	0.7083 ± 0.0123
DeepSeek-V3	Enhanced metadata	Prompt 2	Dataset 1	0.8903 ± 0.0178	0.6822 ± 0.0065	0.7725 ± 0.0109	0.6822 ± 0.0311	0.6752 ± 0.0093	0.6785 ± 0.0178
DeepSeek-V3	Enhanced metadata	Prompt 2	Dataset 2	0.6530 ± 0.0109	0.6043 ± 0.0073	0.6277 ± 0.0083	0.8347 ± 0.0113	0.6179 ± 0.0064	0.7101 ± 0.0042
DeepSeek-V3	Enhanced metadata	Prompt 2	Dataset 3	0.9547 ± 0.0134	0.6863 ± 0.0055	0.7985 ± 0.0031	0.8851 ± 0.0026	0.6321 ± 0.0044	0.7375 ± 0.0034
GPT-4o	Name only	Prompt 1	Dataset 1	0.9305 ± 0.0084	0.6850 ± 0.0055	0.7891 ± 0.0046	0.7073 ± 0.0326	0.6858 ± 0.0039	0.6962 ± 0.0174
GPT-4o	Name only	Prompt 1	Dataset 2	0.6710 ± 0.0019	0.5457 ± 0.0046	0.6019 ± 0.0020	0.8404 ± 0.0131	0.5620 ± 0.0043	0.6735 ± 0.0059
GPT-4o	Name only	Prompt 1	Dataset 3	0.9821 ± 0.0071	0.5518 ± 0.0097	0.7066 ± 0.0068	0.8757 ± 0.0091	0.4993 ± 0.0118	0.6359 ± 0.0072
GPT-4o	Name only	Prompt 2	Dataset 1	0.9417 ± 0.0114	0.6739 ± 0.0055	0.7856 ± 0.0017	0.6866 ± 0.0120	0.6726 ± 0.0066	0.6795 ± 0.0078
GPT-4o	Name only	Prompt 2	Dataset 2	0.6657 ± 0.0070	0.5324 ± 0.0070	0.5916 ± 0.0071	0.8309 ± 0.0088	0.5481 ± 0.0092	0.6604 ± 0.0067
GPT-4o	Name only	Prompt 2	Dataset 3	0.9897 ± 0.0031	0.5302 ± 0.0041	0.6905 ± 0.0036	0.8791 ± 0.0126	0.4761 ± 0.0037	0.6176 ± 0.0051
GPT-4o	Basic metadata	Prompt 1	Dataset 1	0.9326 ± 0.0123	0.6817 ± 0.0068	0.7876 ± 0.0082	0.6704 ± 0.0180	0.6776 ± 0.0061	0.6740 ± 0.0116
GPT-4o	Basic metadata	Prompt 1	Dataset 2	0.6605 ± 0.0064	0.5300 ± 0.0095	0.5881 ± 0.0083	0.8466 ± 0.0153	0.5439 ± 0.0075	0.6623 ± 0.0096
GPT-4o	Basic metadata	Prompt 1	Dataset 3	0.9851 ± 0.0052	0.5643 ± 0.0075	0.7175 ± 0.0053	0.8836 ± 0.0106	0.5081 ± 0.0057	0.6451 ± 0.0019
GPT-4o	Basic metadata	Prompt 2	Dataset 1	0.9281 ± 0.0016	0.6722 ± 0.0083	0.7797 ± 0.0059	0.6658 ± 0.0044	0.6696 ± 0.0069	0.6677 ± 0.0054
GPT-4o	Basic metadata	Prompt 2	Dataset 2	0.6528 ± 0.0036	0.5218 ± 0.0098	0.5800 ± 0.0048	0.8400 ± 0.0151	0.5404 ± 0.0094	0.6576 ± 0.0031
GPT-4o	Basic metadata	Prompt 2	Dataset 3	0.9924 ± 0.0021	0.5418 ± 0.0078	0.7009 ± 0.0062	0.8751 ± 0.0111	0.4839 ± 0.0083	0.6232 ± 0.0088
GPT-4o	Enhanced metadata	Prompt 1	Dataset 1	0.9096 ± 0.0166	0.7065 ± 0.0067	0.7952 ± 0.0034	0.6800 ± 0.0251	0.7033 ± 0.0038	0.6913 ± 0.0134
GPT-4o	Enhanced metadata	Prompt 1	Dataset 2	0.6710 ± 0.0017	0.5651 ± 0.0052	0.6135 ± 0.0034	0.8544 ± 0.0121	0.5799 ± 0.0048	0.6909 ± 0.0020
GPT-4o	Enhanced metadata	Prompt 1	Dataset 3	0.9846 ± 0.0131	0.5798 ± 0.0033	0.7298 ± 0.0062	0.8862 ± 0.0197	0.5274 ± 0.0022	0.6612 ± 0.0072
GPT-4o	Enhanced metadata	Prompt 2	Dataset 1	0.9316 ± 0.0118	0.6965 ± 0.0124	0.7971 ± 0.0124	0.6657 ± 0.0103	0.6930 ± 0.0120	0.6790 ± 0.0072

GPT-4o	Enhanced metadata	Prompt 2	Dataset 2	0.6646 ± 0.0012	0.5401 ± 0.0092	0.5959 ± 0.0058	0.8267 ± 0.0077	0.5585 ± 0.0065	0.6666 ± 0.0045
GPT-4o	Enhanced metadata	Prompt 2	Dataset 3	0.9894 ± 0.0047	0.5608 ± 0.0018	0.7159 ± 0.0018	0.8892 ± 0.0051	0.5049 ± 0.0027	0.6441 ± 0.0036

Note: Each row reports mean ± SD across three independent repeats for one model-input-prompt-dataset condition. Value-level and point-level Micro precision, recall and F1 are shown in separate columns. Dataset 1, Explicit Anomaly Dataset; Dataset 2, Format and Coding Anomaly Dataset; Dataset 3, Semantic-Context-Dependent Anomaly Dataset. Name only, Basic metadata and Enhanced metadata correspond to name, metadata1 and metadata3, respectively, in the evaluation scripts.

Table S19. Complete model and prompt contrasts in the PhenoCurator simulation-based benchmark.

Endpoint	Metric	Comparison (A vs B; subset)	Estimate A	Estimate B	A-B (95% CI)	Permutation P	Holm-adjusted P	Clusters	Strata
Value	Micro F1	DeepSeek-V3 vs GPT-4o; overall	0.7301	0.6981	0.0320 (0.0195 to 0.0449)	<0.0001	0.0006	200	54
Value	Micro Precision	DeepSeek-V3 vs GPT-4o; overall	0.8369	0.8602	-0.0233 (-0.0411 to -0.0061)	0.0116	0.0696	200	54
Value	Micro Recall	DeepSeek-V3 vs GPT-4o; overall	0.6517	0.5933	0.0584 (0.0419 to 0.0750)	<0.0001	0.0006	200	54
Value	Micro F1	DeepSeek-V3 vs GPT-4o; Prompt 1	0.7258	0.7032	0.0225 (0.0093 to 0.0356)	0.0010	0.0030	200	27
Value	Micro Precision	DeepSeek-V3 vs GPT-4o; Prompt 1	0.8329	0.8586	-0.0257 (-0.0466 to -0.0066)	0.0149	0.0745	200	27
Value	Micro Recall	DeepSeek-V3 vs GPT-4o; Prompt 1	0.6474	0.6011	0.0463 (0.0297 to 0.0630)	<0.0001	0.0006	200	27
Value	Micro F1	DeepSeek-V3 vs GPT-4o; Prompt 2	0.7345	0.6930	0.0415 (0.0279 to 0.0553)	<0.0001	0.0006	200	27
Value	Micro Precision	DeepSeek-V3 vs GPT-4o; Prompt 2	0.8409	0.8618	-0.0209 (-0.0365 to -0.0050)	0.0194	0.0776	200	27

Value	Micro Recall	DeepSeek-V3 vs GPT-4o; Prompt 2	0.6561	0.5855	0.0706 (0.0523 to 0.0883)	<0.0001	0.0006	200	27
Value	Micro F1	Prompt 2 vs Prompt 1; overall	0.7138	0.7145	-0.0008 (-0.0051 to 0.0032)	0.7273	0.7273	200	54
Value	Micro Precision	Prompt 2 vs Prompt 1; overall	0.8513	0.8457	0.0056 (0.0001 to 0.0109)	0.0559	0.1677	200	54
Value	Micro Recall	Prompt 2 vs Prompt 1; overall	0.6208	0.6242	-0.0034 (-0.0085 to 0.0018)	0.2002	0.2002	200	54
Value	Micro F1	Prompt 2 vs Prompt 1; DeepSeek-V3	0.7345	0.7258	0.0087 (0.0019 to 0.0154)	0.0134	0.0268	200	27
Value	Micro Precision	Prompt 2 vs Prompt 1; DeepSeek-V3	0.8409	0.8329	0.0081 (-0.0010 to 0.0175)	0.1191	0.2382	200	27
Value	Micro Recall	Prompt 2 vs Prompt 1; DeepSeek-V3	0.6561	0.6474	0.0087 (0.0001 to 0.0172)	0.0408	0.0816	200	27
Value	Micro F1	Prompt 2 vs Prompt 1; GPT-4o	0.6930	0.7032	-0.0102 (-0.0147 to -0.0058)	<0.0001	0.0006	200	27
Value	Micro Precision	Prompt 2 vs Prompt 1; GPT-4o	0.8618	0.8586	0.0032 (-0.0017 to 0.0085)	0.2861	0.2861	200	27
Value	Micro Recall	Prompt 2 vs Prompt 1; GPT-4o	0.5855	0.6011	-0.0156 (-0.0214 to -0.0098)	<0.0001	0.0006	200	27
Observation point	Micro F1	DeepSeek-V3 vs GPT-4o; overall	0.7049	0.6626	0.0424 (0.0268 to 0.0585)	<0.0001	0.0006	200	54
Observation point	Micro Precision	DeepSeek-V3 vs GPT-4o; overall	0.8031	0.8002	0.0028 (-0.0207 to 0.0251)	0.8138	1.0000	200	54

Observation point	Micro Recall	DeepSeek-V3 vs GPT-4o; overall	0.6347	0.5797	0.0550 (0.0370 to 0.0733)	<0.0001	0.0006	200	54
Observation point	Micro F1	DeepSeek-V3 vs GPT-4o; Prompt 1	0.7008	0.6700	0.0308 (0.0141 to 0.0475)	0.0006	0.0018	200	27
Observation point	Micro Precision	DeepSeek-V3 vs GPT-4o; Prompt 1	0.8008	0.8050	-0.0041 (-0.0303 to 0.0220)	0.7702	1.0000	200	27
Observation point	Micro Recall	DeepSeek-V3 vs GPT-4o; Prompt 1	0.6294	0.5875	0.0420 (0.0239 to 0.0599)	<0.0001	0.0006	200	27
Observation point	Micro F1	DeepSeek-V3 vs GPT-4o; Prompt 2	0.7090	0.6551	0.0540 (0.0364 to 0.0710)	<0.0001	0.0006	200	27
Observation point	Micro Precision	DeepSeek-V3 vs GPT-4o; Prompt 2	0.8053	0.7955	0.0098 (-0.0150 to 0.0320)	0.4282	1.0000	200	27
Observation point	Micro Recall	DeepSeek-V3 vs GPT-4o; Prompt 2	0.6399	0.5719	0.0680 (0.0474 to 0.0883)	<0.0001	0.0006	200	27
Observation point	Micro F1	Prompt 2 vs Prompt 1; overall	0.6821	0.6854	-0.0033 (-0.0086 to 0.0019)	0.2144	0.2144	200	54
Observation point	Micro Precision	Prompt 2 vs Prompt 1; overall	0.8004	0.8029	-0.0025 (-0.0105 to 0.0055)	0.5651	1.0000	200	54
Observation point	Micro Recall	Prompt 2 vs Prompt 1; overall	0.6059	0.6085	-0.0026 (-0.0084 to 0.0030)	0.3707	0.3707	200	54
Observation point	Micro F1	Prompt 2 vs Prompt 1; DeepSeek-V3	0.7090	0.7008	0.0082 (-0.0004 to 0.0170)	0.0662	0.1324	200	27
Observation point	Micro Precision	Prompt 2 vs Prompt 1; DeepSeek-V3	0.8053	0.8008	0.0044 (-0.0090 to 0.0179)	0.5486	1.0000	200	27

Observation point	Micro Recall	Prompt 2 vs Prompt 1; DeepSeek-V3	0.6399	0.6294	0.0104 (0.0010 to 0.0197)	0.0311	0.0622	200	27
Observation point	Micro F1	Prompt 2 vs Prompt 1; GPT-4o	0.6551	0.6700	-0.0149 (-0.0217 to -0.0083)	<0.0001	0.0006	200	27
Observation point	Micro Precision	Prompt 2 vs Prompt 1; GPT-4o	0.7955	0.8050	-0.0095 (-0.0207 to 0.0019)	0.1151	0.6905	200	27
Observation point	Micro Recall	Prompt 2 vs Prompt 1; GPT-4o	0.5719	0.5875	-0.0156 (-0.0221 to -0.0093)	<0.0001	0.0006	200	27

Note: Differences are estimate A minus estimate B. Tests were paired cluster permutation tests at the variable level; confidence intervals were obtained by variable-level cluster bootstrap. Holm adjustment was applied within each endpoint-metric family. All prespecified contrasts are shown, irrespective of statistical significance.

Table S20. Complete omnibus tests of repeat-run effects in the PhenoCurator simulation-based benchmark.

Endpoint	Subset	Paired variables	Run 1 mean F1	Run 2 mean F1	Run 3 mean F1	Friedman statistic	Raw P	Holm-adjusted P
Value	overall	200	0.7035	0.7023	0.7030	1.9200	0.3829	1.0000
Value	DeepSeek-V3; prompt1	200	0.7151	0.7145	0.7140	0.4623	0.7936	1.0000
Value	DeepSeek-V3; prompt2	200	0.7228	0.7203	0.7208	3.0511	0.2175	1.0000
Value	GPT-4o; prompt1	200	0.6946	0.6943	0.6942	0.4576	0.7955	1.0000
Value	GPT-4o; prompt2	200	0.6816	0.6800	0.6831	0.6717	0.7147	1.0000
Observation point	overall	200	0.6885	0.6874	0.6882	0.9100	0.6344	1.0000
Observation point	DeepSeek-V3; prompt1	200	0.7012	0.7013	0.6995	0.4623	0.7936	1.0000
Observation point	DeepSeek-V3; prompt2	200	0.7092	0.7093	0.7080	2.3085	0.3153	1.0000
Observation point	GPT-4o; prompt1	200	0.6792	0.6772	0.6811	2.0704	0.3551	1.0000
Observation point	GPT-4o; prompt2	200	0.6644	0.6617	0.6641	1.4737	0.4786	1.0000

Note: Holm adjustment was applied across the five prespecified subsets within each endpoint. All omnibus tests are shown.

Table S21. Complete pairwise repeat-run comparisons in the PhenoCurator simulation-based benchmark.

Endpoint	Subset	Comparison	Paired variables	Mean difference	Median difference	Permutation P	Holm-adjusted P
Value	overall	run1-run2	200	0.0012	0.0015	0.5451	1.0000
Value	overall	run1-run3	200	0.0005	0.0010	0.7721	1.0000
Value	overall	run2-run3	200	-0.0007	0.0001	0.6917	1.0000
Value	DeepSeek-V3; prompt1	run1-run2	200	0.0006	-0.0004	0.8805	1.0000
Value	DeepSeek-V3; prompt1	run1-run3	200	0.0012	-0.0003	0.7498	1.0000
Value	DeepSeek-V3; prompt1	run2-run3	200	0.0005	0.0000	0.8798	1.0000
Value	DeepSeek-V3; prompt2	run1-run2	200	0.0025	0.0016	0.5291	1.0000
Value	DeepSeek-V3; prompt2	run1-run3	200	0.0020	0.0008	0.6163	1.0000
Value	DeepSeek-V3; prompt2	run2-run3	200	-0.0005	0.0000	0.9134	1.0000
Value	GPT-4o; prompt1	run1-run2	200	0.0002	0.0011	0.9575	1.0000
Value	GPT-4o; prompt1	run1-run3	200	0.0004	-0.0018	0.9401	1.0000
Value	GPT-4o; prompt1	run2-run3	200	0.0001	0.0000	0.9713	1.0000
Value	GPT-4o; prompt2	run1-run2	200	0.0016	0.0003	0.6509	1.0000
Value	GPT-4o; prompt2	run1-run3	200	-0.0015	0.0006	0.6721	1.0000
Value	GPT-4o; prompt2	run2-run3	200	-0.0031	-0.0028	0.4027	1.0000
Observation point	overall	run1-run2	200	0.0011	0.0014	0.6424	1.0000
Observation point	overall	run1-run3	200	0.0003	0.0001	0.8861	1.0000
Observation point	overall	run2-run3	200	-0.0008	-0.0004	0.6922	1.0000
Observation point	DeepSeek-V3; prompt1	run1-run2	200	-0.0001	-0.0012	0.9777	1.0000
Observation point	DeepSeek-V3; prompt1	run1-run3	200	0.0017	0.0000	0.7041	1.0000
Observation point	DeepSeek-V3; prompt1	run2-run3	200	0.0018	-0.0006	0.6707	1.0000

Observation point	DeepSeek-V3; prompt2	run1-run2	200	-0.0001	0.0009	0.9798	1.0000
Observation point	DeepSeek-V3; prompt2	run1-run3	200	0.0012	0.0000	0.8138	1.0000
Observation point	DeepSeek-V3; prompt2	run2-run3	200	0.0013	-0.0007	0.7991	1.0000
Observation point	GPT-4o; prompt1	run1-run2	200	0.0020	0.0019	0.6940	1.0000
Observation point	GPT-4o; prompt1	run1-run3	200	-0.0019	0.0002	0.7147	1.0000
Observation point	GPT-4o; prompt1	run2-run3	200	-0.0039	-0.0059	0.3604	1.0000
Observation point	GPT-4o; prompt2	run1-run2	200	0.0027	0.0033	0.5207	1.0000
Observation point	GPT-4o; prompt2	run1-run3	200	0.0002	0.0012	0.9583	1.0000
Observation point	GPT-4o; prompt2	run2-run3	200	-0.0025	-0.0019	0.5837	1.0000

Note: Differences follow the order shown in the Comparison column. Paired sign-flip permutation tests used 10,000 permutations. Holm adjustment was applied across the three pairwise comparisons within each endpoint-subset family. All pairwise comparisons are shown.

PhenoCurator NHANES evaluation and human-review methods

The NHANES evaluation comprised a cross-model comparison and a within-model input-information analysis. For the cross-model comparison, DeepSeek-V3 and locally deployed Meta-Llama-3-70B-Instruct (hereafter, Llama3-70B) were applied to the same variables using Prompt 2 and the Enhanced metadata condition, with each model run independently three times. For the input-information analysis, DeepSeek-V3 was applied to the same variables using Prompt 2 under the Name only, Basic metadata and Enhanced metadata conditions, with each condition run independently three times and all other inference settings held constant. Both analyses used the same 114 NHANES variables: 58 laboratory, 23 examination, 30 questionnaire, and 3 demographic variables. They also used a random sample of 1,000 adults aged at least 20 years from the NHANES 1999-2000 cycle. Because Llama3-70B was evaluated only under Enhanced metadata, cross-model comparisons were restricted to that condition.

Model-generated candidate missing-value codes and candidate anomalous values were normalized and deduplicated at the variable, candidate-type and canonical-value levels. Before review, all candidates were pooled and source-masked, so assessors did not know which model generated a candidate or how often it appeared across repeats. The final pool comprised 2,270 unique candidates. Two assessors supplied usable initial independent labels for 2,148 candidates. The other 122 candidates were excluded from the dual-initial-label agreement analysis: 97 observed generic missing markers were confirmed using a prespecified deterministic rule that required the canonical marker to be present in the source data; one missing-value-code candidate and 24 anomalous-value candidates did not have two usable initial labels. Candidates with discordant or incomplete initial labels underwent a predefined second review; no third-party adjudication was used. Model-level summaries used the final reviewed labels, whereas inter-assessor agreement was calculated from the initial independent annotations.

Two assessors with experience in phenotype metadata curation and data-quality assessment conducted the review using prespecified principles. The official NHANES codebook was the primary evidence source. For categorical and ordinal variables, assessors prioritised the Code or Value and Value Description fields, including official missing and skip codes. For continuous variables, assessors considered the stated range, units, detection-limit information, variable description and observed distribution. Missing-value and anomalous-value candidates were judged separately. Values were not endorsed as anomalies solely

because they were extreme or statistically unusual; assessors also considered plausible disease states, demographic and questionnaire context, and data-collection mechanisms. Confirmed denoted a candidate directly supported as a missing or anomalous value by the available evidence. Plausible denoted a candidate that remained a reasonable quality-control concern but lacked sufficient evidence for definitive confirmation. Not endorsed denoted a candidate not supported as missing or anomalous by the available evidence. Assessor-identified missed values were stored separately and were not counted in the denominator of model-generated candidate-support rates.

Candidate-support rate was the proportion of generated candidates labelled confirmed or plausible. Review-derived reference-set coverage was calculated against a non-exhaustive reference set assembled from reviewed model candidates and assessor-identified missed values. Because the NHANES experiment did not exhaustively annotate every variable-value pair, candidate-support rate is not equivalent to precision and review-derived reference-set coverage is not equivalent to recall. Repeat-run stability was summarized using pairwise Jaccard similarity across the three runs.

For inferential cross-model comparisons, the 114 variables were treated as paired clusters. Candidate-support rate and review-derived reference-set coverage were compared between DeepSeek-V3 and Llama3-70B using variable-cluster permutation tests with 10,000 permutations and variable-cluster bootstrap 95% confidence intervals based on 5,000 replicates. The same procedure was used to compare global pairwise Jaccard similarity for all candidates, anomalous-value candidates, and missing-value-code candidates. Holm correction was applied separately to the two rate outcomes and the three Jaccard outcomes. Within each model and candidate field, repeat-run differences in candidate count per variable were tested using paired Friedman tests, followed by all pairwise paired sign-flip permutation tests with 10,000 permutations. Omnibus P values were Holm-adjusted across the six model-field combinations, and pairwise P values were Holm-adjusted within each model-field family.

PhenoCurator NHANES evaluation results

In the Prompt 2 and Enhanced metadata cross-model comparison, the three-run union contained 1,016 DeepSeek-V3 candidates and 1,506 Llama3-70B candidates. Confirmed-or-plausible candidate-support rates were 99.8% and 88.2%, respectively, whereas review-derived reference-set coverage was 48.5% and 63.5%. DeepSeek-V3 therefore produced a smaller candidate set with higher support and greater repeat stability. Llama3-70B covered more of the review-derived reference set but produced more not-endorsed candidates and showed lower repeat stability. These quantities describe a support-coverage trade-off and should not be interpreted as conventional precision or recall. Initial inter-assessor agreement was complete for missing-value-code candidate labels ($n = 57$) but lower for anomalous-value candidate labels ($n = 2{,}091$; exact agreement, 0.358; Cohen's kappa, 0.184); the corresponding confirmed-or-plausible anomalous-value candidate-set mean Jaccard was 0.707. The asymmetric cross-tabulation indicates systematic differences in assessor thresholds, particularly at the boundary between plausible and not endorsed, together with uncertainty from context-dependent values; the disagreement therefore cannot be attributed solely to model error. Missing-value-code candidates were more stable because many corresponded to explicit codebook-defined missing codes, whereas anomalous-value candidates depended more on semantic context and model-prompt interpretation. These findings support the use of PhenoCurator to generate prioritised candidates for manual review and identify anomalous-value interpretation as a target for future model and prompt improvement.

In the Prompt 2 and Enhanced metadata comparison, DeepSeek-V3 had a significantly higher candidate-support rate than Llama3-70B (difference, 0.1162; 95% CI, 0.0680 to 0.1693; Holm-adjusted $P = 0.0002$). Llama3-70B had numerically higher review-derived reference-set coverage, but the difference was not statistically significant after accounting for variable clustering (DeepSeek-V3 minus Llama3-70B, -0.1501; 95% CI, -0.3295 to 0.0206; $P = 0.0982$). DeepSeek-V3 also had higher pairwise Jaccard similarity for all candidates (difference, 0.1839; $P = 0.0003$), anomalous-value candidates (0.1506; $P = 0.0007$), and missing-value-code candidates (0.1591; $P = 0.0003$). No candidate-count omnibus or pairwise repeat-run comparison remained significant after Holm correction. These tests quantify repeat-run count effects but do not replace the candidate-set Jaccard analysis. Complete significant and non-significant results are reported in Tables S26-S28.

Table S22. NHANES candidate support and review-derived reference-set coverage by model.

Model	Candidate category	Distinct candidates, n	Confirmed, n	Plausible, n	Not endorsed, n	Candidate-support rate, %	Review-derived reference-set coverage, %
DeepSeek-V3	All	1016	451	563	2	99.8%	48.5%
Llama3-70B	All	1506	567	761	178	88.2%	63.5%
DeepSeek-V3	Anomalous	872	313	558	1	99.9%	44.8%
Llama3-70B	Anomalous	1353	428	756	169	87.5%	60.8%
DeepSeek-V3	Missing-value code	144	138	5	1	99.3%	97.9%
Llama3-70B	Missing-value code	153	139	5	9	94.1%	98.6%

Note: Candidate-support rate, confirmed and plausible model-generated candidates divided by all model-generated candidates in the distinct three-run union. Review-derived reference-set coverage, model-generated confirmed-or-plausible candidates divided by all confirmed-or-plausible values in the corresponding non-exhaustive review-derived reference set. The reference set contained 2,092 values overall, including 1,946 anomalous values and 146 missing-value codes; coverage should not be interpreted as recall.

Table S23. Inter-assessor agreement for NHANES variable types and candidate labels.

Initial candidate-label cross-tabulation (n = 2,148)

Assessor 1 label	Assessor 2: Confirmed	Assessor 2: Plausible	Assessor 2: Not endorsed	**Row total**
Confirmed	321	437	9	767
Plausible	7	320	12	339
Not endorsed	2	875	165	1,042
Column total	330	1,632	186	2,148

Initial variable-type cross-tabulation (n = 114)

Assessor 1 type	Assessor 2: Continuous	Assessor 2: Discrete	Assessor 2: Nominal	Assessor 2: Ordinal	**Row total**
Continuous	78	1	0	0	79
Discrete	0	8	0	0	8
Nominal	0	0	13	0	13
Ordinal	0	0	0	14	14
Column total	78	9	13	14	114

Agreement summary

Review target	Metric	n	Exact agreement	Mean Jaccard	Cohen's kappa
Variable type	Four-category label	114	0.991	—	0.982
All candidate values	Three-category label	2,148	0.375	—	0.202

Missing-value-code candidate values	Three-category label	57	1.000	—	1.000
Anomalous-value candidate values	Three-category label	2,091	0.358	—	0.184
Missing-value-code candidate set	Confirmed	112 variables	1.000	1.000	—
Missing-value-code candidate set	Confirmed or plausible	112 variables	1.000	1.000	—
Anomalous-value candidate set	Confirmed	103 variables	0.670	0.770	—
Anomalous-value candidate set	Confirmed or plausible	103 variables	0.505	0.707	—

Note: Rows show Assessor 1 annotations and columns show Assessor 2 annotations for the 2,148 candidates with two usable initial labels. The other 122 comprised 97 observed generic missing markers handled by the stated deterministic rule, one missing-value-code candidate and 24 anomalous-value candidates without two usable initial labels. Candidate-set exact agreement is the proportion of eligible variables for which the assessors selected identical sets. Jaccard similarity is the intersection divided by the union and was averaged across eligible variables. Agreement statistics use the initial independent annotations, whereas model summaries use the final reviewed labels.

Table S24. NHANES candidate-set stability across repeated runs.

Model	All-candidate mean pairwise Jaccard	Anomalous-value-candidate mean pairwise Jaccard	Missing-value-code-candidate mean pairwise Jaccard
DeepSeek-V3	0.449 ± 0.010	0.333 ± 0.005	1.000 ± 0.000
Llama3-70B	0.265 ± 0.029	0.182 ± 0.028	0.841 ± 0.002

Note: Values are mean ± SD across the three pairwise comparisons among three repeated runs for each model. Jaccard similarity compares the distinct candidate sets generated in each run and does not assess candidate correctness.

Table S25. DeepSeek-V3 results across NHANES input-information levels.

Outcome, mean ± SD	Name only	Basic metadata	Enhanced metadata
Variable type matched at least one assessor	0.933 ± 0.005	0.962 ± 0.005	0.965 ± 0.000
Variable type matched both assessors	0.924 ± 0.005	0.953 ± 0.005	0.956 ± 0.000
Conditional variable-type accuracy (n = 113)	0.932 ± 0.005	0.962 ± 0.005	0.965 ± 0.000
Missing-value-code candidates per run	143.0 ± 0.0	143.3 ± 0.6	144.0 ± 0.0
Confirmed missing-value-code candidates per run*	137.0 ± 0.0	137.3 ± 0.6	138.0 ± 0.0
Plausible missing-value-code candidates per run	5.0 ± 0.0	5.0 ± 0.0	5.0 ± 0.0

Outcome, mean ± SD	Name only	Basic metadata	Enhanced metadata
Not-endorsed missing-value-code candidates per run	1.0 ± 0.0	1.0 ± 0.0	1.0 ± 0.0
Anomalous-value candidates per run	318.7 ± 46.5	302.7 ± 5.0	456.0 ± 57.7
Confirmed anomalous-value candidates per run	165.3 ± 10.1	151.3 ± 20.7	175.0 ± 11.3
Plausible anomalous-value candidates per run	153.3 ± 41.1	151.0 ± 19.1	280.7 ± 66.7
Not-endorsed anomalous-value candidates per run	0.0 ± 0.0	0.3 ± 0.6	0.3 ± 0.6

Note: Name only, variable name or identifier; Basic metadata (metadata1), name, variable class, description, data type, unit or valid range, and available enumerations when applicable; Enhanced metadata (metadata3), the Basic metadata fields plus structured phenotype semantics generated by PhenoAM, including available feature and qualifier information such as anatomy, orientation, measurement tool and unit. "Match with at least one assessor" denotes agreement with either initial assessor; "match with both assessors" denotes agreement with both. Conditional variable-type accuracy is restricted to the 113 variables for which the two assessors assigned identical type labels. Assessors evaluated a source-masked pool deduplicated across inputs and runs. *Generic missing markers were counted as confirmed only when observed in the source data. This within-model analysis characterises input sensitivity and must not be used for cross-model comparison (Tables S22-S25).

Table S26. Complete cross-model inferential comparisons in the NHANES evaluation.

Outcome family	Metric	DeepSeek-V3	Llama3-70B	Difference (95% CI)	Permutation P	Holm-adjusted P	Variable clusters
Reviewed-candidate outcome	Candidate-support rate	0.9980	0.8818	0.1162 (0.0680 to 0.1693)	<0.0001	0.0002	114
Reviewed-candidate outcome	Review-derived reference-set coverage	0.4847	0.6348	-0.1501 (-0.3295 to 0.0206)	0.0982	0.0982	114
Pairwise Jaccard	All candidates	0.4491	0.2652	0.1839 (0.0875 to 0.3022)	<0.0001	0.0003	114
Pairwise Jaccard	Anomalous-value candidates	0.3329	0.1823	0.1506 (0.0583 to 0.2699)	0.0007	0.0007	114
Pairwise Jaccard	Missing-value-code candidates	1.0000	0.8409	0.1591 (0.1138 to 0.2049)	<0.0001	0.0003	114

Note: Differences are DeepSeek-V3 minus Llama3-70B. Model comparisons were restricted to Prompt 2 with Enhanced metadata. Candidate-support rate and review-derived reference-set coverage are not equivalent to precision and recall because the NHANES review was candidate-based rather than exhaustive. Holm adjustment was applied separately within the two reviewed-candidate outcomes and the three Jaccard outcomes. All prespecified comparisons are shown.

Table S27. Complete omnibus tests of repeat-run candidate-count effects in the NHANES evaluation.

Model	Candidate field	Paired variables	Run 1 mean count	Run 2 mean count	Run 3 mean count	Friedman statistic	Raw P	Holm-adjusted P
DeepSeek-V3	All candidates	114	5.6579	4.6930	5.4386	3.1465	0.2074	1.0000
DeepSeek-V3	Anomalous-value candidates	114	4.3947	3.4298	4.1754	3.1465	0.2074	1.0000
DeepSeek-V3	Missing-value-code candidates	114	1.2632	1.2632	1.2632	0.0000	1.0000	1.0000
Llama3-70B	All candidates	114	5.4912	6.8333	7.0965	5.5032	0.0638	0.3829
Llama3-70B	Anomalous-value candidates	114	4.3421	5.6579	5.8684	2.7015	0.2590	1.0000
Llama3-70B	Missing-value-code candidates	114	1.1491	1.1754	1.2281	3.1957	0.2023	1.0000

Note: Candidate counts were calculated per variable. Holm adjustment was applied across all six model-field omnibus tests. All omnibus tests are shown.

Table S28. Complete pairwise repeat-run candidate-count comparisons in the NHANES evaluation.

Model	Candidate field	Comparison	Paired variables	Mean count difference	Permutation P	Holm-adjusted P
DeepSeek-V3	All candidates	run1-run2	114	0.9649	0.4377	1.0000
DeepSeek-V3	All candidates	run1-run3	114	0.2193	0.8683	1.0000
DeepSeek-V3	All candidates	run2-run3	114	-0.7456	0.4763	1.0000
DeepSeek-V3	Anomalous-value candidates	run1-run2	114	0.9649	0.4391	1.0000
DeepSeek-V3	Anomalous-value candidates	run1-run3	114	0.2193	0.8664	1.0000
DeepSeek-V3	Anomalous-value candidates	run2-run3	114	-0.7456	0.4693	1.0000
DeepSeek-V3	Missing-value-code candidates	run1-run2	114	0.0000	1.0000	1.0000
DeepSeek-V3	Missing-value-code candidates	run1-run3	114	0.0000	1.0000	1.0000
DeepSeek-V3	Missing-value-code candidates	run2-run3	114	0.0000	1.0000	1.0000
Llama3-70B	All candidates	run1-run2	114	-1.3421	0.1595	0.3981
Llama3-70B	All candidates	run1-run3	114	-1.6053	0.1327	0.3981

Llama3-70B	All candidates	run2-run3	114	-0.2632	0.8501	0.8501
Llama3-70B	Anomalous-value candidates	run1-run2	114	-1.3158	0.1734	0.4770
Llama3-70B	Anomalous-value candidates	run1-run3	114	-1.5263	0.1590	0.4770
Llama3-70B	Anomalous-value candidates	run2-run3	114	-0.2105	0.8762	0.8762
Llama3-70B	Missing-value-code candidates	run1-run2	114	-0.0263	0.6649	0.6649
Llama3-70B	Missing-value-code candidates	run1-run3	114	-0.0789	0.0973	0.2919
Llama3-70B	Missing-value-code candidates	run2-run3	114	-0.0526	0.3195	0.6389

Note: Differences follow the order shown in the Comparison column. Paired sign-flip permutation tests used 10,000 permutations. Holm adjustment was applied across the three pairwise comparisons within each model-field family. All pairwise comparisons are shown.

Section 3. Distribution Fitting in PhenoCurator

PhenoCurator profiles the distributional characteristics of quantitative variables by fitting predefined parametric candidate distributions according to variable type. The following methods specify the candidate families, fitting procedures, goodness-of-fit diagnostics, and selection rule used for continuous and discrete count variables. Aggregate fitting outcomes are reported subsequently.

Data Preparation and Candidate Models

Each quantitative variable is represented by one row of the integrated Bigtable, with measurements stored across sample columns. Values are converted to numeric form, and missing values are excluded before fitting. Continuous variables with fewer than two non-missing observations or constant values are not assigned a parametric distribution. Discrete count variables require at least three non-missing observations, nonnegative integer values, and nonconstant values. Variables that do not meet these conditions are retained with an appropriate final status.

The candidate distributions are Normal, log-normal, exponential, and Cauchy for continuous variables, and Poisson and negative binomial for discrete count variables. Log-normal models are evaluated only when all observations are strictly positive; exponential models are evaluated only when all observations are nonnegative.

Fitting and Goodness-of-Fit Diagnostics

Candidate distributions are fitted by maximum likelihood using the fitdistrplus package in R. For each successful fit, the Akaike Information Criterion and Bayesian Information Criterion are calculated. For continuous variables, the Kolmogorov-Smirnov statistic and its nominal p value are obtained by comparing the empirical distribution with the fitted cumulative distribution function. Because model parameters are estimated from the same observations, these p values are used as exploratory screening diagnostics rather than as stand-alone confirmatory tests.

For discrete variables, observed frequencies from zero to the observed maximum, together with an upper-tail category, are compared with fitted expected frequencies. Adjacent categories are pooled sequentially until the expected count reaches five where possible; any remaining tail is merged with the preceding category. The chi-square statistic is calculated from the pooled categories. Degrees of freedom equal the number of pooled categories minus one minus the number of estimated parameters. The chi-square assessment is not applicable when fewer than two pooled categories remain or when the degrees of freedom are below one.

Model Selection and Final Status

A successfully fitted candidate with an applicable goodness-of-fit assessment is retained for selection when $p > 0.05$. Among retained candidates, the candidate with the lowest Akaike Information Criterion is selected. The Bayesian Information Criterion is retained as a comparative metric and does not determine the selected model. If no candidate meets the threshold, the final status is "no adequate parametric fit". For discrete variables, "goodness-of-fit (GOF) test not applicable" is assigned when no valid chi-square assessment can be calculated. A variable receives a selected distribution when at least one candidate has an applicable goodness-of-fit assessment with $p > 0.05$; the selected model is then the candidate with the lowest AIC among those candidates. This selection does not establish the true data-generating distribution.

Aggregate Fitting Outcomes

Distribution fitting was performed for 6,288 continuous variables and 95 discrete count variables. Table S29 summarizes final fitting outcomes. Table S30 summarizes selected distributions among variables with at least one candidate retained after goodness-of-fit screening.

Table S29. Aggregate final fitting outcomes by variable type.

Variable type	Final status	Number of variables (%)
Continuous	At least one candidate retained after GOF screening	2,703 (43.0%)
Continuous	No candidate retained after GOF screening	3,122 (49.6%)
Continuous	Insufficient data	383 (6.1%)
Continuous	Constant values	64 (1.0%)
Continuous	All distributions failed to fit	16 (0.3%)
Discrete count	At least one candidate retained after GOF screening	16 (16.8%)
Discrete count	No candidate retained after GOF screening	41 (43.2%)
Discrete count	GOF assessment not applicable	7 (7.4%)
Discrete count	Insufficient data	6 (6.3%)
Discrete count	Non-integer values detected	5 (5.3%)
Discrete count	Constant values	20 (21.1%)

Table S30. Selected distributions among variables with at least one candidate retained after goodness-of-fit screening.

Variable type	Selected distribution	Number of retained variables (%)
Continuous	Log-normal	1,765 (65.3%)
Continuous	Normal	911 (33.7%)
Continuous	Cauchy	17 (0.6%)
Continuous	Exponential	10 (0.4%)
Discrete count	Negative Binomial	13 (81.3%)
Discrete count	Poisson	3 (18.8%)

Note: Percentages in Table S29 are calculated within variable type. Percentages in Table S30 are calculated within variables with at least one candidate retained after goodness-of-fit screening for the corresponding type.

Supplementary Tables

Table S31. Statistical Results of Direct Ontology Matching and PhenoAM Matching for Non-Molecular Phenotypes

Ontology mapping result	PhenoAM mapping result			Count	Percentage
	Feature mapping result	Combined qualifier mapping result	PhenoAM mapping category		
Exact match	Exact match	Exact match	Equivalent	153	2.82%
Exact match	Exact match	Only qualifier MT	Equivalent	54	0.99%
Exact match	Partial match	Only qualifier MT	Equivalent	4	0.07%
Exact match	Partial match	Exact match	Expanded	6	0.11%
Exact match	Exact match	Partial match	Expanded	4	0.07%
Exact match	No match	Exact match	No corresponding mapping	1	0.02%
Exact match	No match	Only qualifier MT	No corresponding mapping	6	0.11%
Partial match	Exact match	Exact match	Equivalent	1725	31.74%
Partial match	Exact match	Only qualifier MT	Equivalent	239	4.40%
Partial match	Partial match	Only qualifier MT	Equivalent	104	1.91%
Partial match	Partial match	Exact match	Expanded	538	9.90%
Partial match	Exact match	Partial match	Expanded	667	12.27%
Partial match	Partial match	Partial match	Expanded	712	13.10%
Partial match	No match	Exact match	No corresponding mapping	14	0.26%
Partial match	No match	Partial match	No corresponding mapping	2	0.04%
Partial match	No match	No match	No corresponding mapping	1	0.02%
Partial match	No match	Only qualifier MT	No corresponding mapping	88	1.62%
No match	Exact match	Exact match	Equivalent	576	10.60%
No match	Exact match	Only qualifier MT	Equivalent	70	1.29%
No match	Partial match	Only qualifier MT	Equivalent	26	0.48%
No match	Partial match	Exact match	Expanded	220	4.05%
No match	Exact match	Partial match	Expanded	64	1.18%
No match	Partial match	Partial match	Expanded	141	2.59%
No match	No match	Exact match	No corresponding mapping	2	0.04%
No match	No match	Only qualifier MT	No corresponding mapping	17	0.31%

Note: A data element (DE) here refers to the original phenotype variable term as defined in the source metadata before feature-qualifier decomposition by PhenoAM. To contextualize differences in mapping classification, we performed a descriptive baseline analysis using direct ontology matching of DEs for all non-molecular phenotypes (n = 5,434). In this baseline, 20.5% of phenotypes were classified as No Match. Under the PhenoAM-based multi-resource mapping procedure, 5,303 of 5,434 phenotypes (97.6%)

were classified as Equivalent or Expanded, whereas 131 (2.4%) were classified as No corresponding mapping. These descriptive results show that component-level representation can change mapping classifications by retaining feature and qualifier information that may not be captured when a DE is assessed as a single term.

After PhenoAM-based decomposition, the remaining No corresponding mappings arose predominantly from No match combinations at the feature or qualifier level. This descriptive analysis indicates that component-level mapping can make the sources of non-correspondence more explicit; it does not independently establish semantic accuracy or quantify true novelty. Detailed results are available in the GitHub repository (https://github.com/BioMedBigDataCenter/PhenoAgents).

Supplementary Notes

Note S1. Comparison of HPO, Phenopackets, NIH CDE, and PhenoAM/PhenoDE

This supplementary comparison contains a structured comparison (Table S32) and three concise variable-level examples. Semantic representation is assessed separately. Implementation is compared using FAIR-informed dimensions (Findable, Accessible, Interoperable, and Reusable), with typical research use and support for data collection and participant-data linkage included as additional considerations. The FAIR-informed rows provide a comparative lens rather than a formal FAIR-compliance assessment.

Table S32. Semantic representation and FAIR-informed implementation

Comparison domain	Assessment criterion	HPO	Phenopackets	NIH CDE	PhenoAM/PhenoDE
Semantic representation	Basic representational unit and measurement context	**HPO terms:** contextual details can be captured via additional annotations or relations, but are not intrinsic to the term itself	**Structured schema fields:** populated records can carry measurement context; specific content is supplied by the record creator	**CDE definitions and Forms:** context can be specified through definitions, question text, units, permissible values, instructions, and Form organization	**Feature-Qualifier components:** typed components explicitly represent measurement object, process, and result
	Core information represented	**Concept-level:** abnormal human phenotypes and ontology relations	**Record-level:** machine-readable individual- or biosample-centered phenotypic and related clinical information	**Data-element-level:** standardized CDEs, with Forms organizing CDEs for data collection	**Compositional variable-level:** compositional feature-qualifier definitions of cohort phenotype variables
FAIR-informed implementation (Wilkinson, M. D. et al. The FAIR Guiding Principles for scientific data management and stewardship. Scientific Data 3, 160018 (2016). https://doi.org/10.1038/sdata.2016.18.)	Findable (F): identifiers, metadata, and searchability	**Supported:** stable HPO identifiers and searchable ontology metadata	Indirectly supported: As a schema, a Phenopacket ID is required, but identifier persistence, indexing, and discovery depend on the implementing repository	**Supported:** repository identifiers and searchable CDE/Form metadata	**Supported:** stable feature, qualifier, PhenoDE, and phenotype-variable IDs; metadata searchable in the PhenoDE portal
	Accessible (A): retrieval route and access conditions	**Supported:** terms and ontology metadata are accessible through the HPO web interface and downloadable releases	Indirectly supported: The schema is public, but access to populated records relies on the hosting provider	**Supported:** CDE and Form metadata are publicly accessible through the web repository	**Supported:** PhenoDE metadata are accessible through the PhenoDE portal; participant-level values are governed separately
	Interoperable (I): machine-readable format and semantic linkage	**Ontology model:** HPO is released in OWL, OBO, and JSON formats, with shared identifiers and ontology relations	**Exchange schema:** Phenopackets are defined in Protobuf, support JSON representation, and use ontology/CURIE references	**Data-element model:** NIH CDEs use standardized identifiers and metadata, with machine-readable exports; cross-system use depends on shared CDE adoption or external mappings	**Compositional data-element model**: PhenoDE uses stable identifiers, external semantic mappings, and a JSON Schema for machine-verifiable structure

Comparison domain	Assessment criterion	HPO	Phenopackets	NIH CDE	PhenoAM/PhenoDE
Other implementation considerations	Reusable (R): reusable content and metadata	**Supported (concept reuse):** ontology terms and relations can be reused across annotations and analyses	**Supported (schema/record reuse):** the schema and populated records are reusable with required metadata; record content is supplied by the record creator	**Supported (data-element reuse):** stewarded CDEs and Forms are reusable across studies through standardized definitions and metadata	**Supported (compositional reuse)**: features, qualifiers, PhenoDEs, and mappings can be reused across variables and studies
	Typical research use	Abnormality coding and ontology-based analysis	Exchange of populated phenotypic and clinical records	Study design, multi-center data standardization, and data dictionary sharing	Variable definition and cross-cohort semantic comparison
	Support for data collection and data linkage	**Not supported**: HPO terms annotate findings derived elsewhere; they neither define collection fields nor store raw values	**Indirect support**: The schema does not specify collection workflows; populated records structure and carry observation values after acquisition	**Supported**: CDEs provide standardized definitions for data collection, while data is captured and stored by the study's software systems	Supported: PhenoDE standardizes variable metadata used for data collection; in IHPP, LIMS captures and Bigtable stores participant data using variable IDs mapped to PhenoDE IDs

Note: Abbreviations: HPO, Human Phenotype Ontology; CDE, Common Data Elements; PhenoAM, Phenotype Assembly Method; PhenoDE, Phenome Data Elements. The table uses selected implementation characteristics under these four principles. Bold lead terms are descriptive summaries, not ordinal FAIR scores.
Here, concept-level refers to standardized phenotype concepts and relations; record-level to individual- or biosample-level records containing supplied data values; data-element-level to standardized data-element definitions and Forms used to guide data collection; and compositional variable-level representation refers to explicitly decomposed phenotype definitions, such as feature and qualifier components, used for cross-cohort semantic comparison. In IHPP, the Laboratory Information Management System (LIMS) is used for actual data capture, while Bigtable refers to the integrated participant-level phenotype data matrix.

Together, HPO supports concept-level coding, Phenopackets record-level exchange, NIH CDE data-element standardization, and PhenoAM/PhenoDE compositional variable-level representation for cross-cohort comparison and reuse.

Example 1. Left macular inner-inferior retinal nerve fibre layer thickness.

HPO provides related abnormality concepts (HP:0020119 and HP:0030329) rather than a data-element definition for this quantitative measurement. The Phenopackets schema can encode a populated OCT Measurement for exchange, but the record creator must supply the assay, observation value, ontology terms, and any additional anatomy, laterality, segmentation, or device detail needed for the record. The NIH CDE Form (ID: QJEq7yHBte) provides quadrant-specific retinal nerve fibre layer thickness fields and indicates OD/OS applicability, but the macular, inner, and left-sided context of the IHPP variable is not represented together in a single CDE definition. PhenoDE PDE057024763.1 retains the source-variable definition through a feature plus explicit anatomical, positional, and OCT qualifiers.

Sources for this example:

HPO: https://hpo.jax.org/browse/term/HP:0020119; https://hpo.jax.org/browse/term/HP:0030329

Snapshot of HPO: Abnormal retinal nerve fiber layer morphology (HP:0020119)

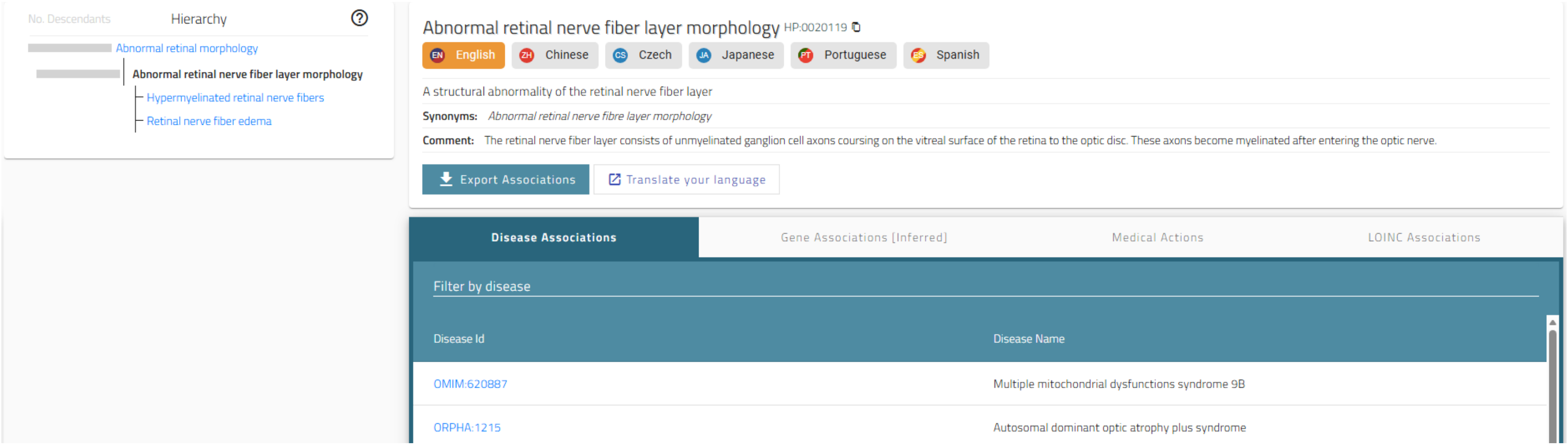

Snapshot of HPO: Retinal thinning on OCT (HP:0030329)

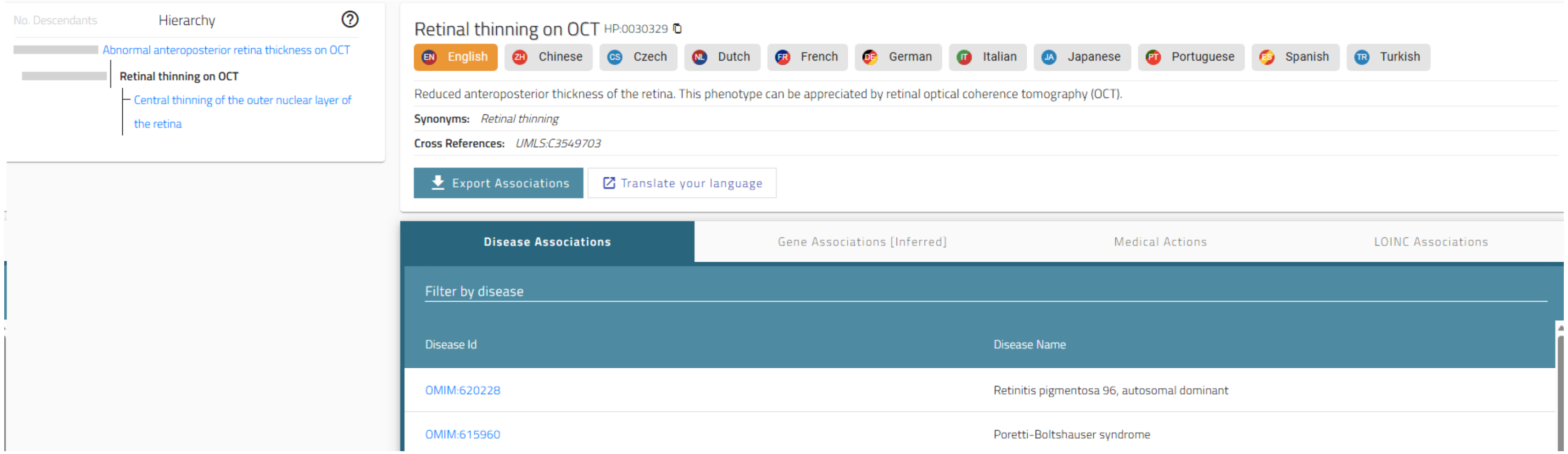

Phenopackets: https://phenopacket-schema.readthedocs.io/en/latest/measurement.html

Snapshot of Phenopackets: Measurement schema and quantitative example

Measurement

The measurement element is used to record individual measurements. It can capture quantitative, ordinal (e.g., absent/present), or categorical measurements.

Data model

Definition of the `Measurement` *element*

Field	Type	Multiplicity	Description
description	string	0..1	free text.
assay	OntologyClass	1..1	Class that describes the assay used to produce the measurement. REQUIRED.
measurement_value	one of {Value | ComplexValue}	1..1	The result of the measurement. REQUIRED.
time_observed	TimeElement	0..1	Time at which measurement was performed. RECOMMENDED.
procedure	Procedure	0..1	Clinical procdure performed to acquire the sample used for the measurement

Examples

The following example shows measurement of platelet count. The result is abnormally low, but in general this element can be used to represent normal or abnormal measurements.


```
measurement:
    assay:
        id: "LOINC:26515-7"
        label: "Platelets [#/volume] in Blood"
    value:
        quantity:
            unit:
                id: "UO:0000316"
                label: "cells per microliter"
            value: 24000.0
            referenceRange:
                unit:
                    id: "UO:0000316"
                    label: "cells per microliter"
                low: 150000.0
                high: 450000.0
    timeObserved:
        timestamp: "2020-10-01T10:54:20.021Z"
```

NIH CDE: https://cde.nlm.nih.gov/formView?tinyId=QJEq7yHBte

Snapshot of NIH CDE: Imaging OCT Analysis - RNFL Thickness Analysis Form

ON THIS PAGE

- Preview
- General Details
  - Status
  - Other Names & Definitions
- Classification
- Display Profiles
  - Properties
  - Identifiers
  - History

Form: Imaging OCT Analysis - RNFL Thickness Analysis

Export | Add to Board | CDEs

Preview

Options

1. Imaging average retinal nerve fiber layer thickness abnormal scale

To be answered for OD and/or OS, as applicable. Choose only one.

○ RNFL thickness is normal (green on the report) ○ RNFL is abnormally thin, in the 5th percentile (yellow on the report) ○ RNFL thickness is in the 1st percentile (red on the report) ○ RNFL is unusually thick, in the 95th percentile (white on the report)

2. Imaging retinal nerve fiber layer thickness in superior quadrant measurement

To be answered for OD and/or OS, as applicable. Record result in microns.

3. Imaging retinal nerve fiber layer thickness in superior quadrant abnormal status

To be answered for OD and/or OS, as applicable. Choose only one.

○ Macula is of normal volume (green on the report) ○ Macular volume is abnormally low, in the 5th percentile (yellow on the report) ○ macular volume is abnormally low, in the 1st percentile (red on the report) ○ Macular volume is abnormally great, in the 95th percentile (pale yellow on the report)

General Details

Name: Imaging OCT Analysis - RNFL Thickness Analysis

Description: Contains data elements related to commonly collected OCT RNFL thickness analysis information used in MS clinical research studies. [Examples of CDEs included: Signal Strength; Average RNFL Thickness]

Used By: NINDS

Is a Bundle: No

Copyrighted: No, this material is freely available, without restriction

Origin:

Version: 1.0

Steward: NINDS

Last import: 04/13/2021 @ 8:09AM

Created: 09/24/2015 @ 4:09AM

Updated: 04/13/2021 @ 9:34AM

Status

Registration Status: Qualified

Administrative Status: Published

Publish Date: 09/24/2015

PhenoAM/PhenoDE: https://www.biosino.org/PhenoDE/browser/detail/PDE057024763.1

Snapshot of PhenoAM/PhenoDE: PDE057024763.1 record and data profile

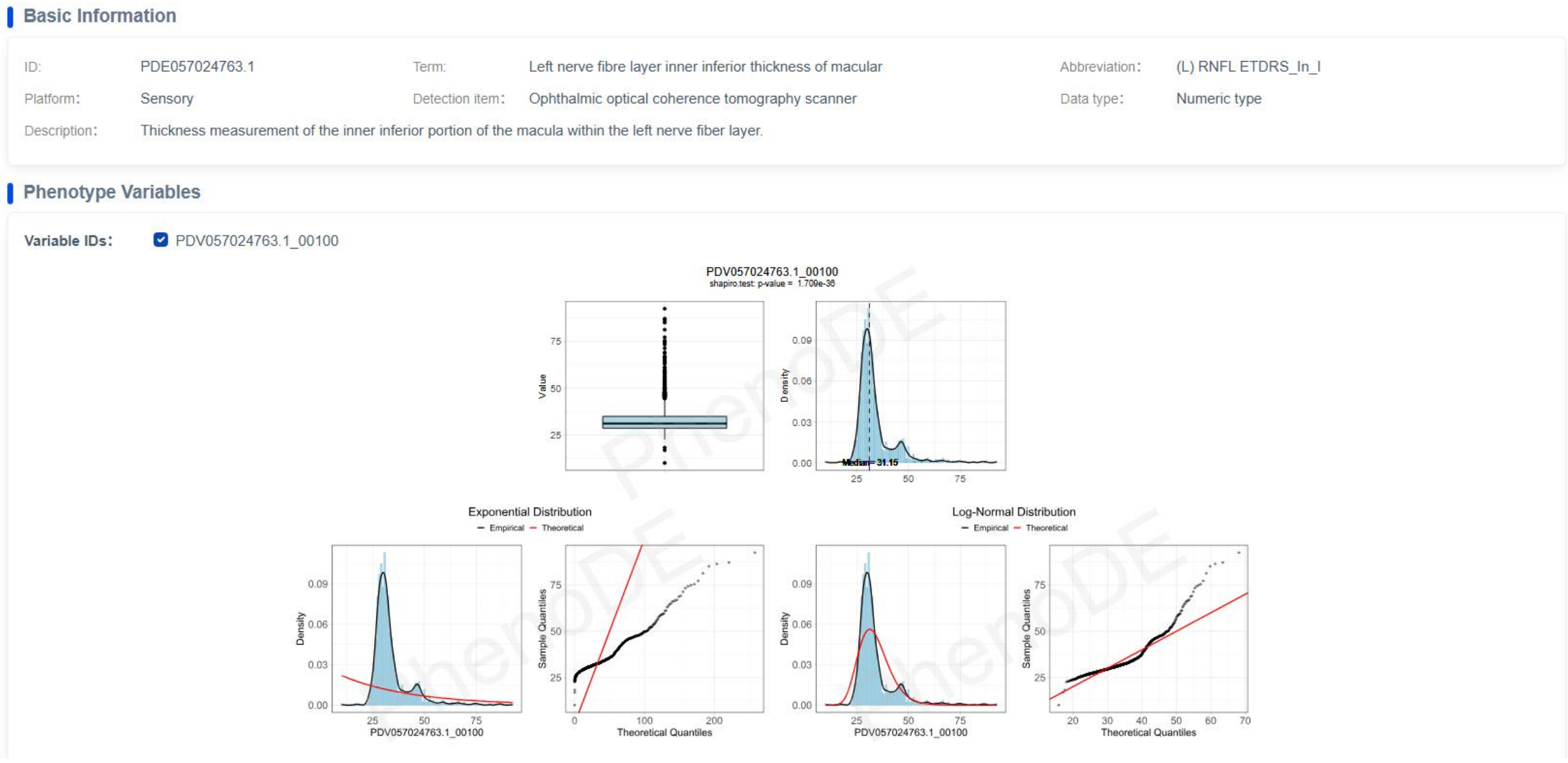

Snapshot of PhenoAM/PhenoDE: feature-qualifier representation of PDE057024763.1

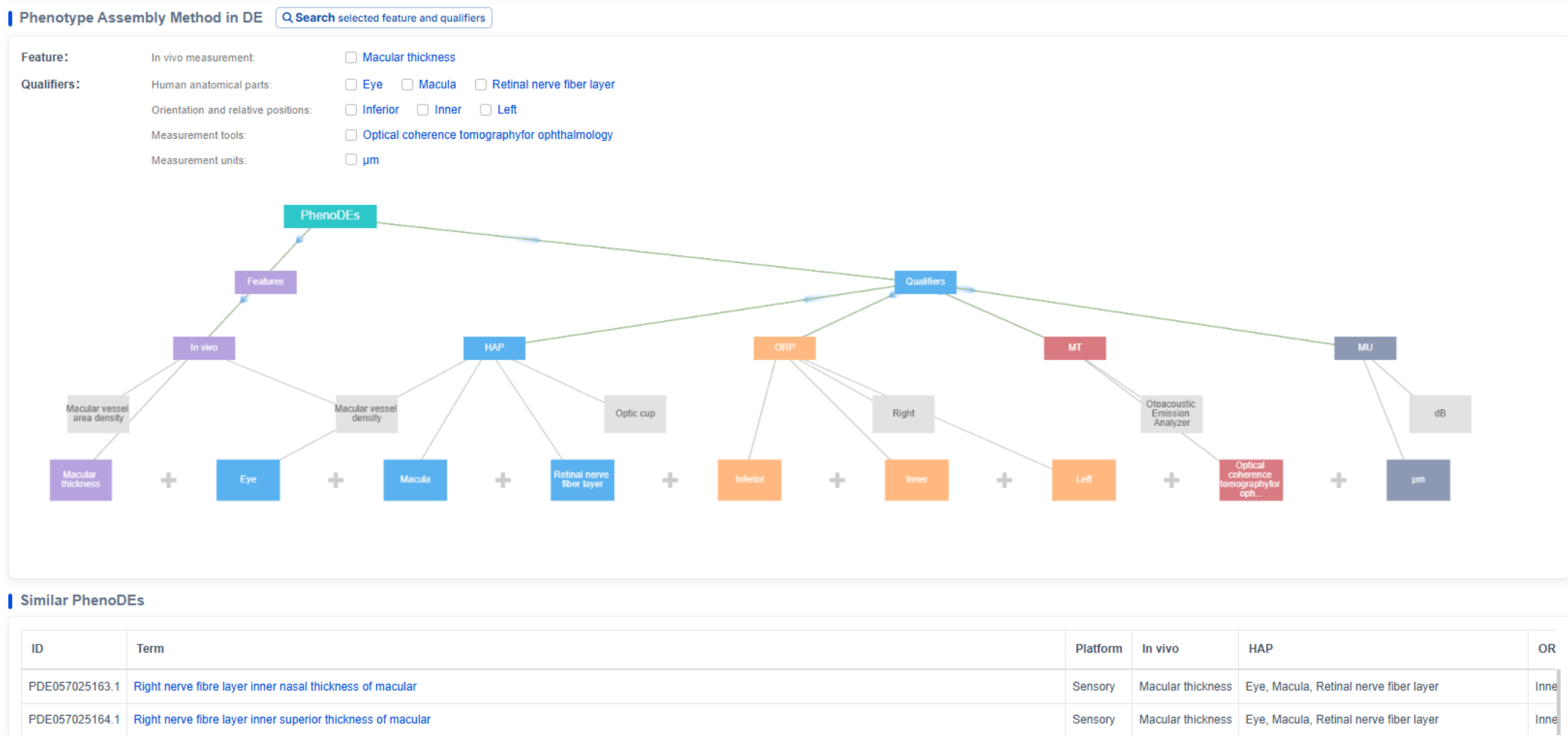

Example 2. Fasting C-peptide.

HPO terms represent abnormal or elevated circulating C-peptide rather than the raw fasting serum measurement. The Phenopackets schema can encode a populated Measurement for exchange, but the record creator must supply the assay, biosample, fasting context, observation value, unit, and any additional analyzer or measurement details through available schema fields, ontology terms, or external metadata. The NIH CDE (ID: Q1WQFm5sHC) captures the fasting C-peptide construct but not serum, analyzer, or unit. PhenoDE PDE024002618.1 explicitly records fasting C-peptide together with serum, fasting status, analyzer, and ng/mL, and specifies a numeric data type.

Sources for this example:

HPO: https://hpo.jax.org/browse/term/HP:0030794; https://hpo.jax.org/browse/term/HP:0030796

Snapshot of HPO: Abnormal circulating C-peptide concentration (HP:0030794)

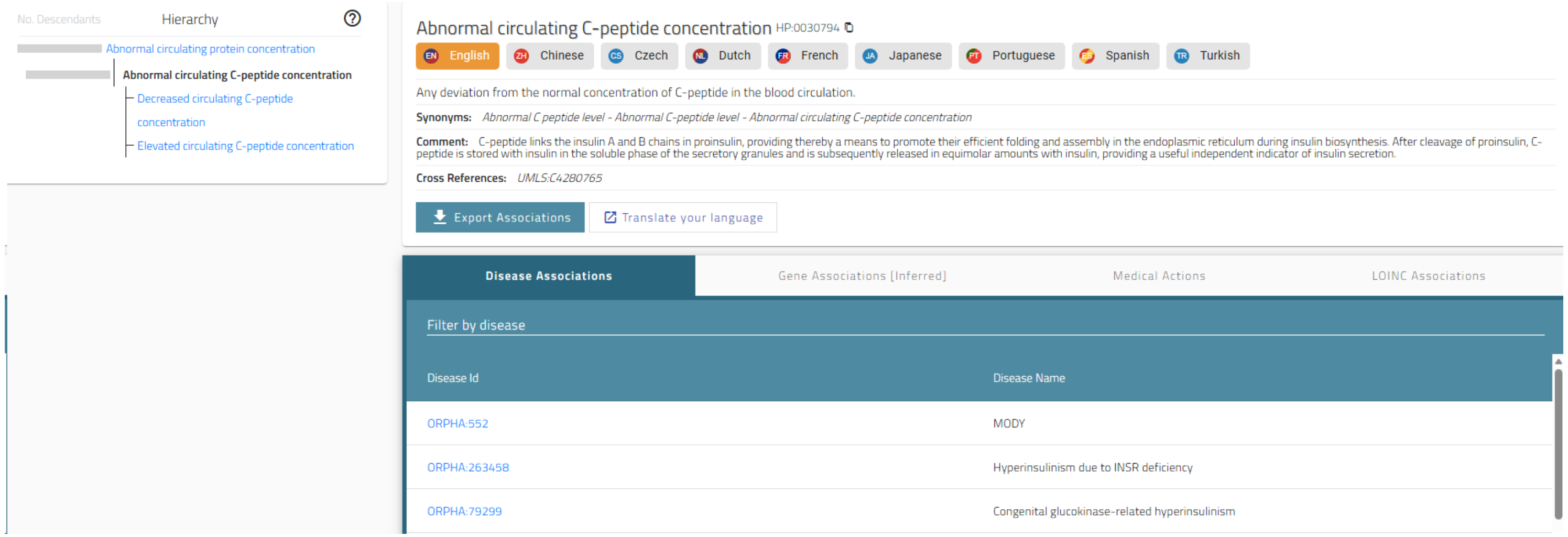

Snapshot of HPO: Elevated circulating C-peptide concentration (HP:0030796)

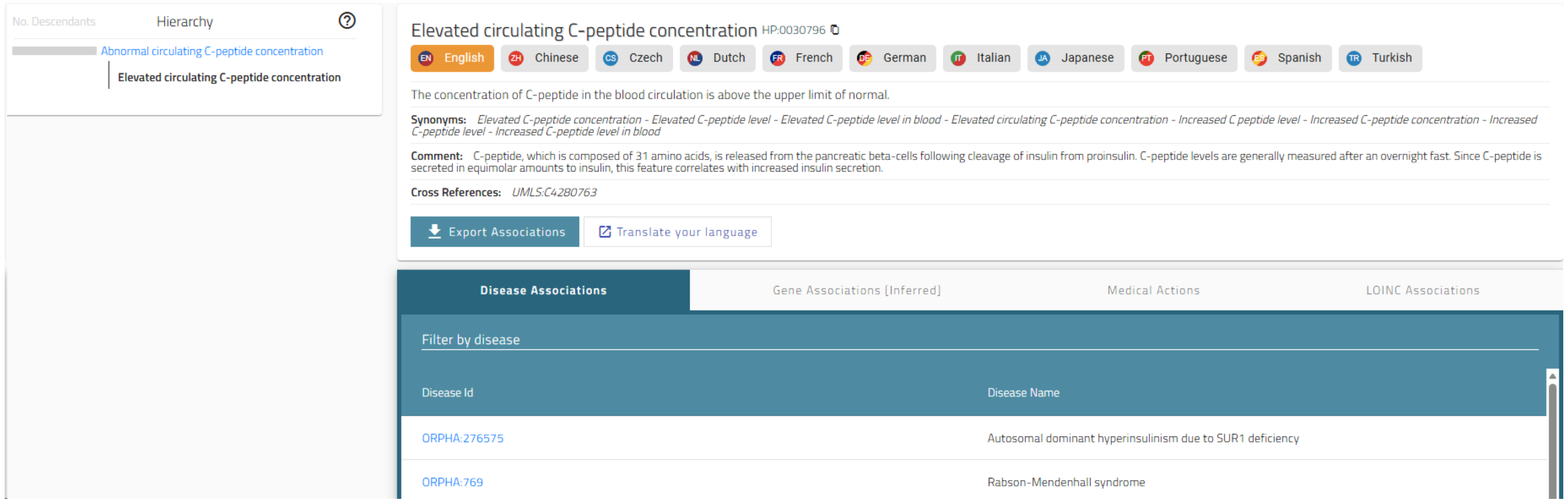

Phenopackets: https://phenopacket-schema.readthedocs.io/en/latest/measurement.html; https://phenopacket-schema.readthedocs.io/en/latest/biosample.html; https://phenopacket-schema.readthedocs.io/en/latest/procedure.html

For the Measurement schema screenshot, see Example 1.

Snapshot of Phenopackets: Biosample schema and example

Biosample

A Biosample refers to a unit of biological material from which the substrate molecules (e.g. genomic DNA, RNA, proteins) for molecular analyses (e.g. sequencing, array hybridisation, mass-spectrometry) are extracted. Examples would be a tissue biopsy, a single cell from a culture for single cell genome sequencing or a protein fraction from a gradient centrifugation. Several instances (e.g. technical replicates) or types of experiments (e.g. genomic array as well as RNA-seq experiments) may refer to the same Biosample.

Data model

Field	Type	Multiplicity	Description
id	string	1..1	Arbitrary identifier. REQUIRED.
individual_id	string	0..1	Arbitrary identifier. RECOMMENDED.
derived_from_id	string	0..1	id of the biosample from which the current biosample was derived (if applicable)
description	string	0..1	arbitrary text
sampled_tissue	OntologyClass	0..1	Tissue from which the sample was taken
sample_type	OntologyClass	0..1	type of material, e.g., RNA, DNA, Cultured cells
phenotypic_features	PhenotypicFeature (List)	0..*	List of phenotypic abnormalities of the sample. RECOMMENDED.
measurements	Measurement (List)	0..*	List of measurements of the sample
taxonomy	OntologyClass	0..1	Species of the sampled individual
time_of_collection	TimeElement	0..1	Age of the proband at the time the sample was taken. RECOMMENDED.
histological_diagnosis	OntologyClass	0..1	Disease diagnosis that was inferred from the histological examination. RECOMMENDED.
tumor_progression	OntologyClass	0..1	Indicates primary, metastatic, recurrent. RECOMMENDED.
tumor_grade	OntologyClass	0..1	Term representing the tumor grade
pathological_stage	OntologyClass	0..1	Pathological stage, if applicable. RECOMMENDED.
pathological_tnm_finding	OntologyClass (List)	0..*	Pathological TNM findings, if applicable. RECOMMENDED.
diagnostic_markers	OntologyClass (List)	0..*	Clinically relevant biomarkers. RECOMMENDED.
procedure	Procedure	0..1	The procedure used to extract the biosample. RECOMMENDED.
files	File (List)	0..*	list of files related to the biosample, e.g. VCF or other high-throughput sequencing files
material_sample	OntologyClass	0..1	Status of specimen (tumor tissue, normal control, etc.). RECOMMENDED.
sample_processing	OntologyClass	0..1	how the specimen was processed
sample_storage	OntologyClass	0..1	how the specimen was stored

Example

The staging system most often used for bladder cancer is the American Joint Committee on Cancer (AJCC) TNM system. The overall stage is assigned based on the T, N, and M categories (Cancer stage grouping). For instance, stage II (pathological staging) is defined as T2a or T2b, N0, and M0, meaning the cancer has spread into the wall of the bladder.


```yaml
biosample:
  id: "sample1"
  individualId: "patient1"
  description: "Additional information can go here"
  sampledTissue:
      id: "UBERON_0001256"
      label: "wall of urinary bladder"
  histologicalDiagnosis:
      id: "NCIT:C39853"
      label: "Infiltrating Urothelial Carcinoma"
  tumorProgression:
      id: "NCIT:C84509"
      label: "Primary Malignant Neoplasm"
  tumorGrade:
      id: "NCIT:C36136"
      label: "Grade 2 Lesion"
  procedure:
      code:
          id: "NCIT:C5189"
          label: "Radical Cystoprostatectomy"
  files:
      - uri: "file:///data/genomes/urothelial_ca_wgs.vcf.gz"
      individualToFileIdentifiers:
          patient1: "NA12345"
      fileAttributes:
          description: "Urothelial carcinoma sample"
          htsFormat: "VCF"
          genomeAssembly: "GRCh38"
  materialSample:
      id: "EFO:0009655"
      label: "abnormal sample"
  timeOfCollection:
      age:
          iso8601duration: "P52Y2M"
  pathologicalStage:
      id: "NCIT:C28054"
      label: "Stage II"
  pathologicalTnmFinding:
  - id: "NCIT:C48726"
      label: "T2b Stage Finding"
  - id: "NCIT:C48705"
      label: "N0 Stage Finding"
  - id: "NCIT:C48699"
      label: "M0 Stage Finding"
```

Snapshot of Phenopackets: Procedure schema and example

Procedure

The Procedure element represents a clinical procedure performed on a subject. For example a surgical or diagnostic procedure such as a biopsy.

By preference a single `code` to indicate both the procedure and the body site should be used. In cases where this is not possible, the body site should be indicated using a separate ontology class.

Data model

Field	Type	Multiplicity	Description
code	OntologyClass	1..1	clinical procedure performed. REQUIRED.
body_site	OntologyClass	0..1	specific body site where the procedure was performed
performed	TimeElement	0..1	age/time when the procedure was performed

Example

In this example, a skin biopsy from the skin of the forearm is perfomed on an individual who is 25 years old.

```
procedure:
    code:
        id: "NCIT:C28743"
        label: "Punch Biopsy"
    bodySite:
        id: "UBERON:0003403"
        label: "skin of forearm"
    performed:
        age:
            iso8601duration: "P25Y"
```

In this case, the `code` for "Punch biopsy" is not specific to a location, so providing `bodySite` gives additional useful information.

NIH CDE: https://cde.nlm.nih.gov/deView?tinyId=Q1WQFm5sHC

Snapshot of NIH CDE: Laboratory procedure fasting C-peptide value

Pin to Board | Export

12 Views
Updated: 04/13/2021

Laboratory procedure fasting C-peptide value

Warning: This version of the CDE might be out of date. For the most current version, please visit https://www.commondataelements.ninds.nih.gov

Question Text

Submitter did not provide a Question Text

Definition

Subjects/Participants amount of fasting C-peptide in the blood.

Data Type: Number

Version:

1

Steward: NINDS

Used By: NINDS

Origin:

Data Type Details

Data Type: Number

Minimum Value:

Maximum Value:

Precision:

Unit of Measure:

CDE Details

Other Names & Definitions

Name: Laboratory procedure fasting C-peptide value

Tags:

Name Source:

Definitions

Definition: Subjects/Participants amount of fasting C-peptide in the blood.

Tags:

Name Source:

Concepts

There are no concepts.

Identifiers

Source	Id	Version
NLM	Q1WQFm5sHC	1
NINDS	C19727	1
BRICS Variable Name	LabPrcdFastCPeptideVal	

PhenoAM/PhenoDE: https://www.biosino.org/PhenoDE/browser/detail/PDE024002618.1

Snapshot of PhenoAM/PhenoDE: PDE024002618.1 record and data profile

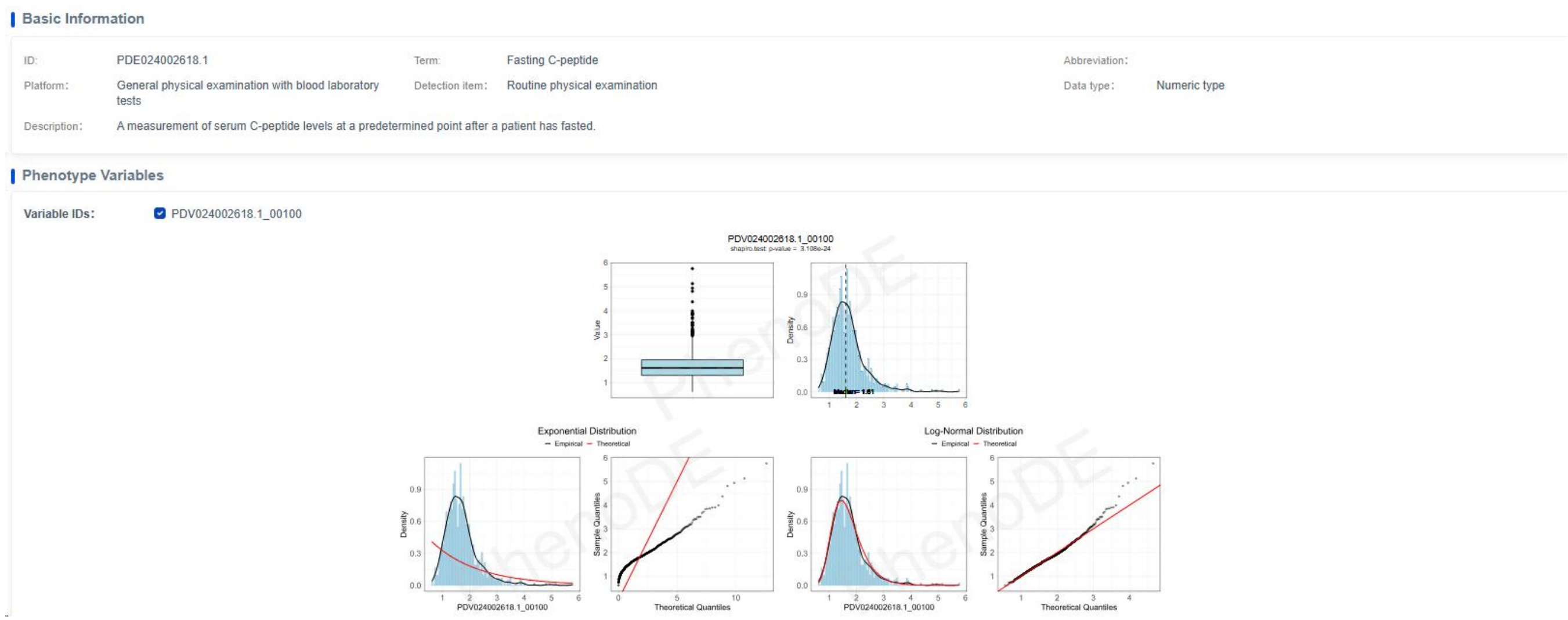

Snapshot of PhenoAM/PhenoDE: feature-qualifier representation of PDE024002618.1

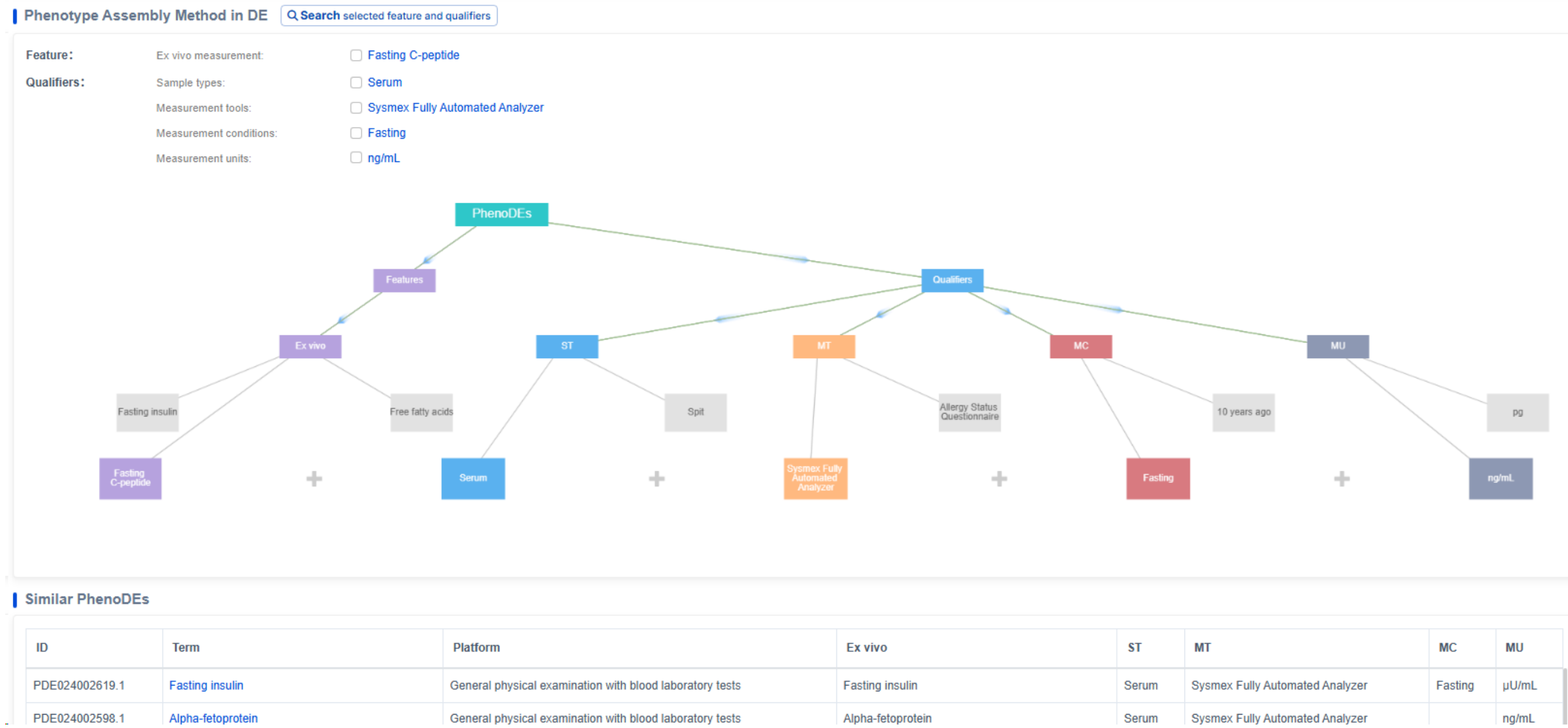

Example 3. Age when started drinking beer.

HPO provides an abnormal-alcohol-consumption term rather than the beer-specific initiation-age questionnaire variable. The Phenopackets schema can carry a populated response for exchange, but question wording, drinker applicability, beverage specificity, skip logic, and response values must be supplied by the record creator or represented through external descriptions. The NIH CDE (ID: FtNNFMKHCM4) captures alcohol-use initiation age but is beverage-general. PhenoDE PDE025002687.1 retains the beer-specific variable through an initiation-age feature together with Drinker, Drinking and tea consumption history, and Beer qualifiers, and specifies a numeric data type.

Sources for this example:

HPO: https://hpo.jax.org/browse/term/HP:0430037

Snapshot of HPO: Abnormal alcohol consumption (HP:0430037)

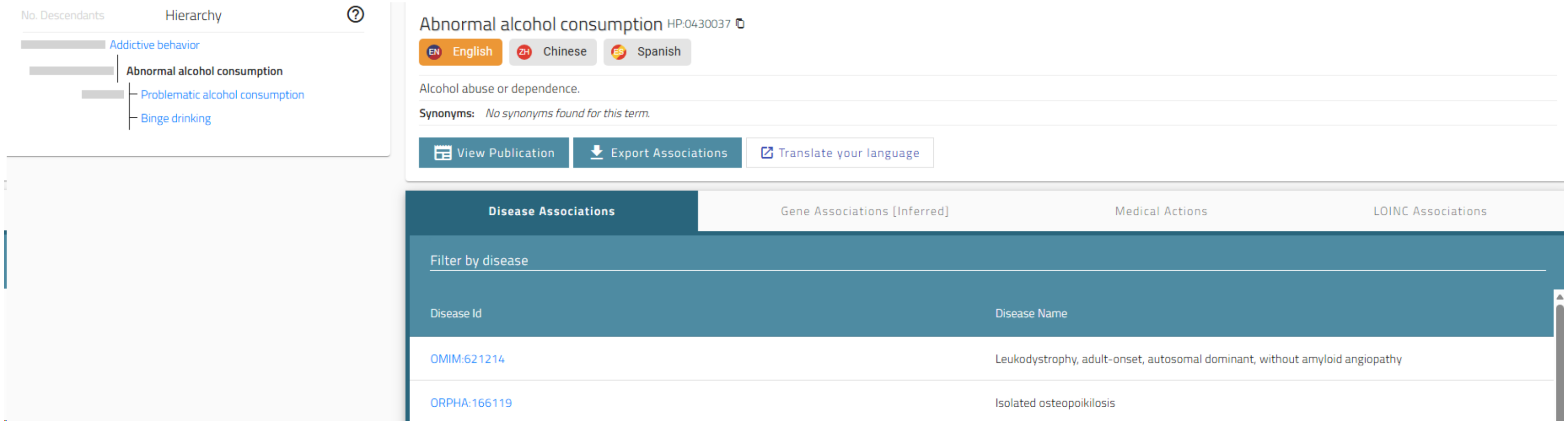

Phenopackets: https://phenopacket-schema.readthedocs.io/en/latest/measurement.html

For the Measurement schema screenshot, see Example 1.

NIH CDE: https://cde.nlm.nih.gov/deView?tinyId=FtNNFMKHCM4

Snapshot of NIH CDE: Alcohol use started age value

Pin to Board   Export

3 Views
Updated: 04/13/2021

Alcohol use started age value

Warning: This version of the CDE might be out of date. For the most current version, please visit https://www.commondataelements.ninds.nih.gov

Question Text

Submitter did not provide a Question Text

Definition

Age in years when participant/subject started ingesting alcoholic beverages, including social drinking

Data Type: Number

Version:

3.1

Steward: NINDS

Used By: NINDS

Origin:

Data Type Details

Data Type: Number

Minimum Value: 0

Maximum Value: 120

Precision:

Unit of Measure: year

CDE Details

Other Names & Definitions

Name: Alcohol use started age value

Tags:

Name Source:

Definitions

Definition: Age in years when participant/subject started ingesting alcoholic beverages, including social drinking

Tags:

Name Source:

Concepts

There are no concepts.

Identifiers

Source	Id	Version
NLM	FtNNFMKHCM4	3.1
NINDS caDSR	2008447	
BRICS Variable Name	AlcUseStrtAgeVal	
NINDS	C00702	3

PhenoAM/PhenoDE: https://www.biosino.org/PhenoDE/browser/detail/PDE025002687.1

Snapshot of PhenoAM/PhenoDE: PDE025002687.1 record and data profile

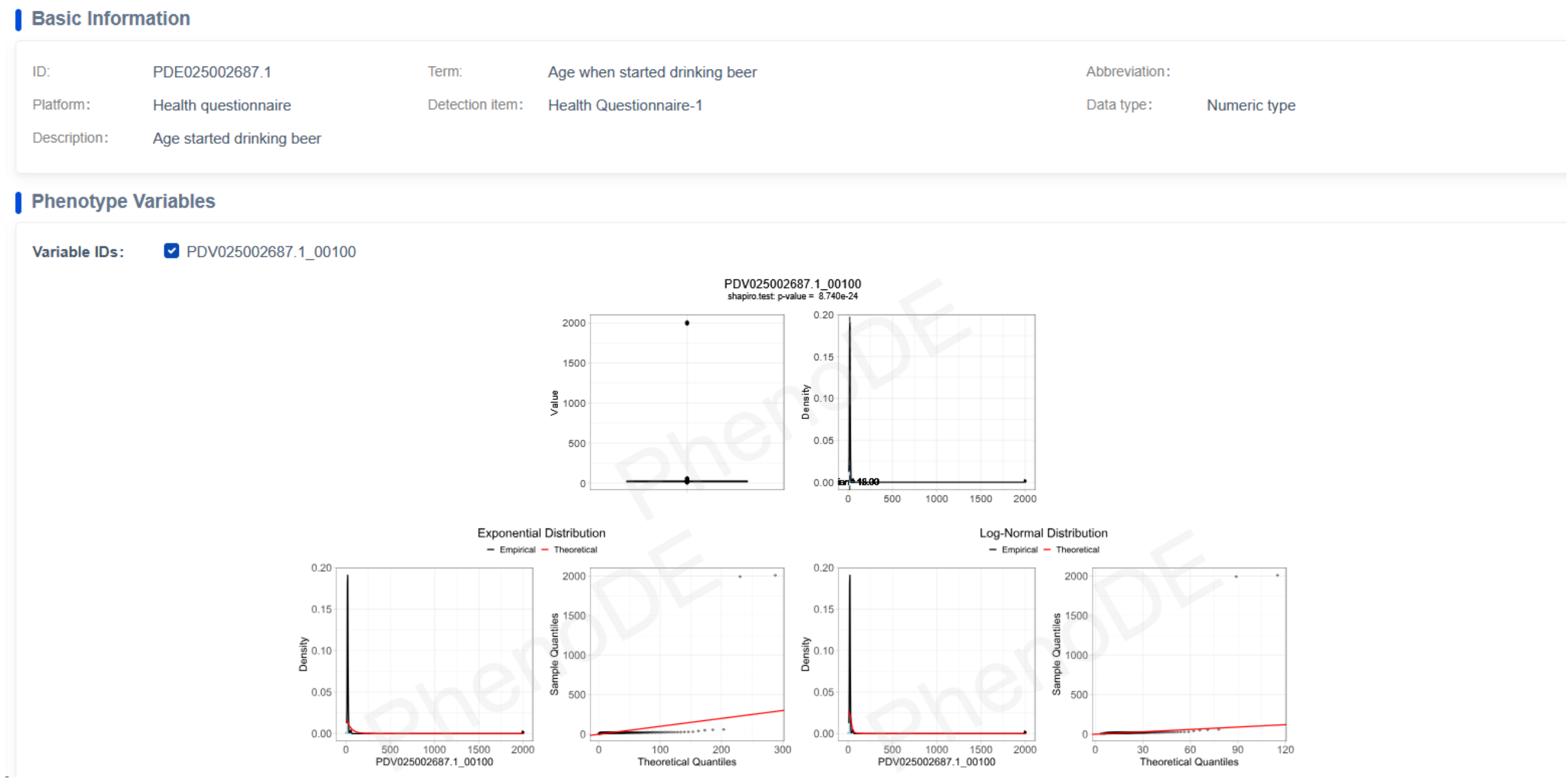

Snapshot of PhenoAM/PhenoDE: feature-qualifier representation of PDE025002687.1

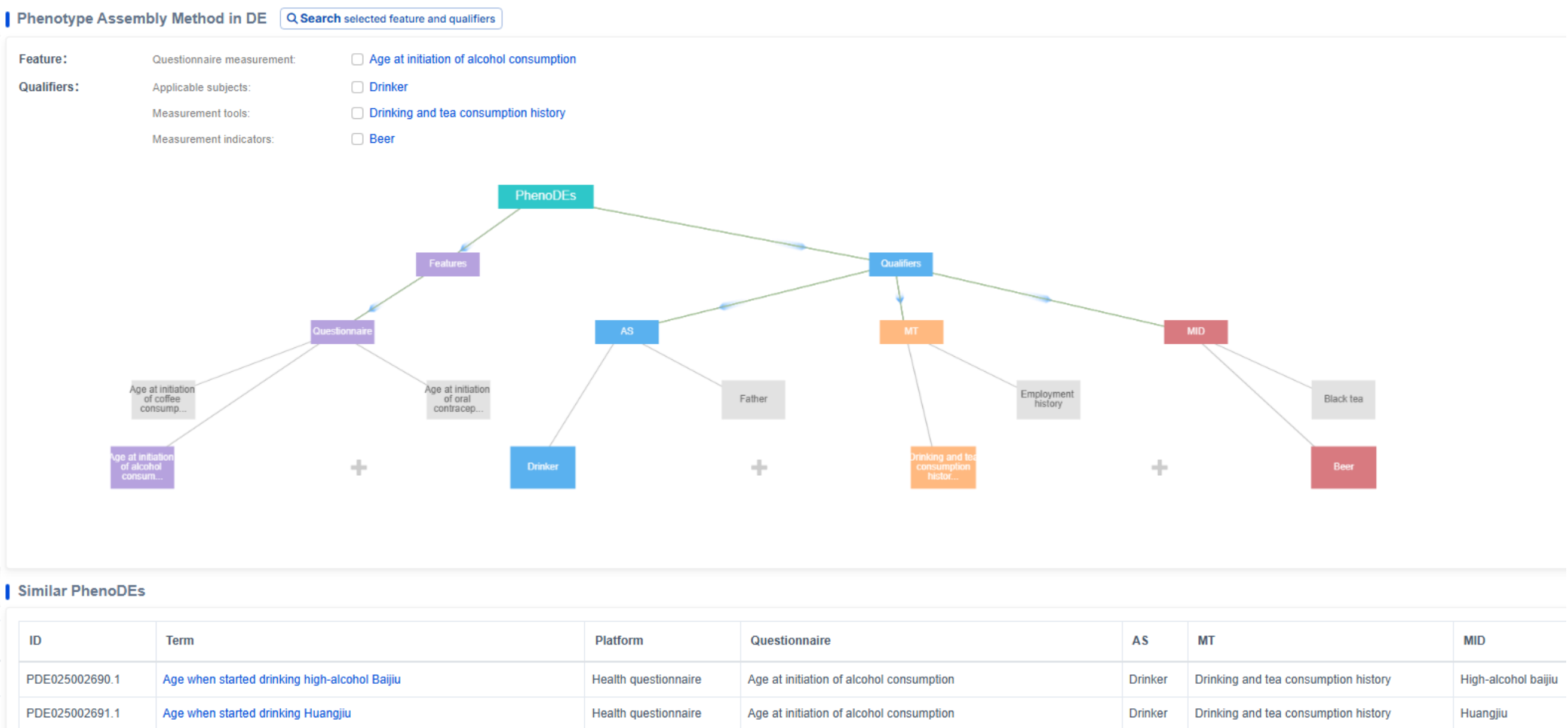

ID	Term	Platform	Questionnaire	AS	MT	MID
PDE025002690.1	Age when started drinking high-alcohol Baijiu	Health questionnaire	Age at initiation of alcohol consumption	Drinker	Drinking and tea consumption history	High-alcohol baijiu
PDE025002691.1	Age when started drinking Huangjiu	Health questionnaire	Age at initiation of alcohol consumption	Drinker	Drinking and tea consumption history	Huangjiu

Note S2. Anatomical classification of HAP qualifiers and associations

For Figure 2d, human anatomical part (HAP) qualifiers were aggregated by body region for descriptive statistical visualization. The scheme contains five groups and 27 subclasses. Each of the 220 unique HAP qualifiers was assigned to one primary subclass, allowing 6,134 HAP association records to be summarized without double counting. This grouping does not define a formal anatomical hierarchy.

Classification procedure

Assignments were based on the most specific body region identifiable from each HAP term. Specific subregions took precedence over broader regional labels. Explicit broad terms were assigned to General head, General trunk, or General upper limb. Terms denoting the entire body were assigned to Whole body. Terms spanning more than one body region were assigned to Multiple body regions. Terms that could not be assigned to a unique body region from the HAP term alone were assigned to Body region unspecified. Body region unspecified is an operational residual category for descriptive aggregation and does not indicate missing source data.

The five groups and 27 subclasses are descriptive aggregation categories. Anatomical entity type remains conceptually separate from body region and is not used as a grouping dimension in Figure 2d.

Table S33. Summary of body region groups

Body region group	Number of body region subclasses	Number of unique HAP qualifiers	Number of HAP associations
Head and neck	8	122	3621
Trunk	5	42	1399
Upper limb	6	22	343
Lower limb	5	17	443
Whole-body and multi-region	3	17	328
**Total**	**27**	**220**	**6134**

Table S34. Detailed subclass structure

Body region group	Body region subclass	Number of unique HAP qualifiers	Number of HAP associations
Head and neck	Intracranial region	72	285
Head and neck	Facial region	5	51
Head and neck	Ocular and orbital region	16	2188
Head and neck	Auricular region	2	588
Head and neck	Oral region	11	129
Head and neck	Nasal region	6	126
Head and neck	General head	1	6
Head and neck	Cervical region	9	248
Trunk	Thoracic region	19	477
Trunk	Abdominal region	8	454
Trunk	Pelvic and perineal region	8	223
Trunk	Dorsal trunk region	5	129
Trunk	General trunk	2	116
Upper limb	Shoulder region	4	14
Upper limb	Arm region	3	109
Upper limb	Elbow region	1	2
Upper limb	Forearm region	2	6
Upper limb	Hand region	11	201
Upper limb	General upper limb	1	11
Lower limb	Hip region	6	193
Lower limb	Thigh region	3	131
Lower limb	Knee region	1	7

Body region group	Body region subclass	Number of unique HAP qualifiers	Number of HAP associations
Lower limb	Leg region	3	91
Lower limb	Foot region	4	21
Whole-body and multi-region	Whole body	1	100
Whole-body and multi-region	Multiple body regions	8	170
Whole-body and multi-region	Body region unspecified	8	58

Note: Definition of summary measures. Unique HAP qualifier counts refer to distinct qualifier terms. HAP association counts refer to the number of associations assigned to each subclass in the Figure 2d source data. The totals are 220 unique HAP qualifiers and 6,134 HAP associations.

Note S3. Positioning PhenoAM and PhenoDE relative to SNOMED CT postcoordination

SNOMED CT postcoordination and PhenoAM both represent complex meaning using multiple semantic components, but they serve different representational functions. SNOMED CT postcoordination combines SNOMED CT concepts into a single expression governed by Compositional Grammar and concept-model constraints. PhenoAM organizes a research phenotype measurement specification as one measurable feature plus typed qualifiers and materializes the curated combination as a PhenoDE. The comparison below therefore focuses on representational role and operational use rather than treating the two models as equivalent.

Table S35. Comparison of SNOMED CT postcoordination and PhenoAM with PhenoDE

Comparison dimension	SNOMED CT postcoordination	PhenoAM and PhenoDE	Comparison note
Representational target	Represents a clinical meaning, including observable meanings, within SNOMED CT terminology.	Represents a research phenotype measurement specification as a reusable data element.	SNOMED CT formalizes terminology meaning; PhenoAM and PhenoDE organize the specification of a research measurement item.
Composition structure	Combines one or more focus concepts with refinements expressed as attribute–value pairs according to SNOMED CT Compositional Grammar and concept-model rules.	Combines one measurable feature with typed qualifiers assigned to predefined semantic fields.	The distinction is formal terminology composition through relationships versus explicit organization through phenotype-specific metadata fields.
When an exact precoordinated concept is unavailable	A postcoordinated expression may represent a more specific clinical meaning. Alternatively, a new precoordinated concept may be authored in an appropriate SNOMED CT extension.	A study-specific feature–qualifier combination may be registered as a PhenoDE while individual components are linked to available terminology or other resources.	SNOMED CT addresses the gap through terminology expression or extension; PhenoDE addresses it through data-element registration and component-level resource linkage.
Organization of measurement metadata	Observable Entity modeling can use attributes such as Component, Property, Inheres in or Direct site, Technique, Scale type, and Time aspect, subject to the applicable concept-model and MRCM rules. Additional implementation data may be carried by the surrounding information model.	Qualifiers organize measurement object, process, and result information, including site, orientation, sample type, subject applicability, method, tool, condition, indicator, statistical summary, and unit.	PhenoAM provides a consistent phenotype-specific organization of heterogeneous research measurement metadata alongside the terminology-level representation supported by SNOMED CT.
Terminology and resource linkage	A postcoordinated expression is constructed from SNOMED CT concepts; mappings to non-SNOMED resources are outside SNOMED CT Compositional Grammar.	Features and qualifiers may link independently to SNOMED CT, other ontologies, specialized databases, cohort dictionaries, datasets, or literature.	PhenoAM and PhenoDE provide cross-resource metadata organization and component-level linkage when different components are represented most appropriately by different resources.

Comparison dimension	SNOMED CT postcoordination	PhenoAM and PhenoDE	Comparison note
Connection to study variables and identifiers	SNOMED CT expressions can populate fields in an information model, while subject, time, result values, and local variable identifiers are managed by the surrounding data model.	A PhenoDE defines the measurement item, receives a stable identifier, and links to source variable identifiers. Participant values remain in the cohort data table.	Both can separate coded meaning from instance values, while PhenoDE directly integrates the measurement specification with cohort-variable management.
Validation and computational semantics	Compositional Grammar and the MRCM support syntactic and concept-model validation; suitable terminology services can support classification and subsumption testing.	Structural schemas and curation rules support record validation, component mapping, search, and retrieval, but do not provide SNOMED CT-equivalent description-logic classification or semantic-equivalence reasoning.	PhenoAM and PhenoDE provide complementary metadata organization and data-element representation alongside SNOMED CT formal terminology semantics.

Supplementary Example 1. Diameter of left vertebral artery

PhenoDE PDE078057965.1 is an ultrasonography data element with the term “Diameter of left vertebral artery.” Its PhenoAM representation contains feature = Arterial diameter; HAP = Vertebral artery; ORP = Left; MT = Doppler Ultrasound Diagnostic System (Philips EPIQ7); and MU = cm.

Aspect	Confirmed SNOMED CT information	PhenoAM and PhenoDE representation	Comparison note
Core observable	302286009 |Arterial diameter (observable entity)| is an existing SNOMED CT concept.	feature: Arterial diameter	The core measurement property has a direct terminology match.
Measurement object metadata	SNOMED CT contains body-structure concepts for the vertebral artery and the left vertebral artery. Their use to refine an arterial-diameter observable must follow the applicable Observable Entity and MRCM rules.	HAP: Vertebral artery<br>ORP: Left	PhenoAM records anatomical site and laterality in separate phenotype-specific fields that can be searched and compared independently.
Measurement process and result metadata	SNOMED CT can represent terminology-level measurement technique where permitted by the model; implementation details such as a specific instrument model and expected unit may also be carried by the surrounding observation information model.	MT: Doppler Ultrasound Diagnostic System (Philips EPIQ7)<br>MU: cm	PhenoDE retains the study-specific instrument model and expected unit as part of the measurement specification.

Note: A complete SNOMED CT postcoordinated expression is not asserted for this example. Any such expression would require validation against the relevant SNOMED CT edition and the applicable MRCM rules.

Supplementary Example 2. Forced expiratory flow at 50% of FVC

This example illustrates the role of PhenoDE when the core observable meaning is already represented by a single SNOMED CT concept.

**Aspect**	**Confirmed SNOMED CT information**	**PhenoAM and PhenoDE representation**	**Comparison note**
Core observable	1367695001 |Forced expiratory flow rate at 50 percent of vital capacity| is an existing SNOMED CT concept.	feature: Forced expiratory flow at 50% of FVC	The core observable meaning is already represented by a single SNOMED CT concept.
Measurement object metadata	The SNOMED CT concept represents the pulmonary-function observable. Additional anatomical context can be represented through terminology content or the surrounding information model when required.	HAP: Lung	PhenoDE retains the project-level anatomical context as an explicit metadata field.
Measurement process and result metadata	Implementation-specific instrument information and the recorded unit can be represented in the surrounding observation information model, with terminology bindings where appropriate.	MT: Pulmonary Function Tester<br>MU: L/s	PhenoDE places the observable within an explicit cohort measurement specification and retains project-level metadata.

Relation to a conventional Entity-Attribute-Value model

A conventional Entity-Attribute-Value (EAV) model serves a different representational purpose. It provides a flexible storage representation for sparse and heterogeneous data by storing facts as entity, attribute, and value records. Production EAV systems may include rich attribute metadata, data types, permissible values, and validation rules. However, the EAV storage pattern itself does not prescribe which attributes should represent a phenotype feature, anatomical site, measurement condition, method, instrument, statistical summary, or unit. PhenoAM may be implemented using an EAV structure, a relational structure, or a JSON structure; its role is to define and organize phenotype-specific semantic components and the resulting data elements.

Note S4. Positioning PhenoAM and PhenoDE relative to established data-element, terminology, and clinical observation frameworks

The standards and frameworks below support different but connected functions in biomedical information representation and are compared to clarify their respective roles and potential points of alignment with PhenoAM and PhenoDE.

Table S36. Comparison of PhenoAM and PhenoDE with established data-element, terminology, and clinical observation frameworks

Framework	Primary function and purpose	Principal constructs	Representation approach	Governance and interoperability mechanisms	Potential alignment with PhenoAM and PhenoDE
**Conventional CDE approach**	Study data collection and data-element standardization.	CDE specification comprising a designation or question, definition, datatype, and permissible value set.	Predefined data-element specification. CDEs may be assembled into forms or instruments.	Registry-dependent identifiers, versioning, registration status, terminology linkage, and value set governance.	PhenoAM can make measurement semantics associated with a CDE explicit as typed feature and qualifier components. PhenoDE entries could be linked to, or represented within, existing CDE registries after field-level mapping.
**ISO/IEC 11179 metadata registry family**	Domain-independent metadata registry metamodel and governance framework.	Data Element; Data Element Concept, including Object Class and Property; Conceptual Domain; and Value Domain, including datatypes, units, and permissible values.	Formal concept-based metadata registry metamodel separating conceptual meaning from value representation.	Formal mechanisms for identification, definition, registration, classification, versioning, and mapping of metadata items.	PhenoAM components could inform phenotype-specific data-element concepts and associated metadata. PhenoDE identifiers, definitions, versions, concepts, and value domains could be aligned with ISO/IEC 11179 registry constructs after a formal crosswalk is defined.
**NCI caDSR**	Operational biomedical metadata registry and repository for NCI data standards.	CDEs, together with case report forms, data collection templates, and data models as managed repository assets.	ISO/IEC 11179-based concept and value domain metadata registration within an operational biomedical repository.	Managed identifiers, versions, registration status, terminology linkage, value domains, repository services, and machine-accessible interfaces.	PhenoAM could structure the measurement semantics used to define phenotype-oriented CDEs. PhenoDE entries could potentially be registered or linked within a caDSR-like environment, subject to field-level mapping and registry governance requirements.

Framework	Primary function and purpose	Principal constructs	Representation approach	Governance and interoperability mechanisms	Potential alignment with PhenoAM and PhenoDE
**LOINC**	Standard terminology for identifying laboratory tests, clinical measurements, survey items, and other observations.	LOINC term or code defined by Component, Property, Time Aspect, System, Scale, and optional Method Parts.	Observation terminology using a six-part definitional model.	Persistent terminology identifiers, curated term maintenance, and use in clinical interoperability standards including HL7 and FHIR.	A PhenoAM feature and selected qualifiers may align with a LOINC term or its defining Parts when an appropriate concept exists. PhenoDE can retain such mappings as curated external references, while semantics without direct LOINC counterparts remain represented in PhenoDE.
**FHIR Observation**	Instance-level clinical information exchange resource for representing observed values and related context.	Observation resource comprising status, code, subject or focus, effective[x], value[x] or component, and contextual elements such as bodySite or bodyStructure, method, specimen, device, interpretation, and referenceRange.	Resource-based exchange using coded elements, references, profiles, terminology bindings, and extensions.	Machine exchange in JSON, XML, or RDF with profiles, cardinalities, terminology bindings, and validation constraints.	PhenoAM could inform the semantic decomposition of an observation definition. After formal field-level mapping, PhenoDE definitions could potentially inform the coding and contextual metadata used when participant-level observations are represented with FHIR Observation; individual qualifiers may correspond to standard elements or may require profiles or extensions.
**PhenoAM and PhenoDE**	Phenotype-specific semantic decomposition method and standardized data-element resource for heterogeneous deep phenotyping.	PhenoAM: core feature and typed qualifiers covering measurement object, process, result, and applicable subject. PhenoDE: identifiable and reusable data elements assembled using these components.	Compositional representation that externalizes measurement context as reusable semantic components across heterogeneous phenotype domains.	Controlled vocabularies, stable PhenoDE identifiers and versions, external resource mappings, and machine-readable structural specifications.	

Note S5. PhenoAM Annotation Rules

1. Purpose and representation model

These rules define how PhenoAM separates the core measurable characteristic from contextual information when curating PhenoDEs from ex vivo, in vivo, and questionnaire measurements. Each PhenoDE contains exactly one feature and one or more qualifiers. Accordingly, the total qualifier cardinality is 1..n. No individual qualifier category is universally mandatory across all phenotype types; each category is included when applicable, subject to modality- and protocol-specific requirements. Sample Type (ST) is mandatory for ex vivo records. If ST is unavailable in the source metadata, the record is considered incomplete and requires further curation before it is finalized as a PhenoDE.

2. Feature-Qualifier definitions and boundary rule

Feature. The core measurable characteristic that answers what is measured, observed, asked, or derived. Each PhenoDE contains exactly one feature. The feature term should preferably be expressed in singular form where appropriate. Feature class is recorded separately as ex vivo measurement, in vivo measurement, or questionnaire measurement.

Qualifier. A typed contextual component that further specifies the feature with respect to the measurement object, measurement process, measurement result, or applicable subject.

Boundary rule. Use the most concise feature term that still identifies a biologically meaningful and reusable measurand. A familiar compound term, such as arm length, may be retained when a shorter term such as length would be overly generic and would no longer identify a biologically meaningful measurand on its own. If a term can be removed without changing the identity of what is measured, assign it to the appropriate qualifier category.

Semantic overlap. Avoid unnecessary duplication between the feature and qualifiers. Limited overlap may be retained when needed to preserve an established feature label or faithfully represent the source metadata.

3. Assignment workflow

1. **Identify the feature.** Select a concise term that clearly identifies what is measured and record its feature class separately.
2. **Separate contextual information.** Move removable information about the object, process, result, or applicable subject into qualifiers.
3. **Assign qualifier categories.** Use the eleven category definitions and examples provided in Table 1.

4. Check completeness. Record at least one applicable qualifier for every PhenoDE. ST is mandatory for ex vivo records; an ex vivo record without ST is incomplete and requires further curation.

5. Normalize and order. Use controlled terms, avoid unnecessary duplication, and present the feature before the qualifiers.

4. Combination, cardinality, and presentation order

Feature cardinality. Exactly one feature is recorded for each PhenoDE; the feature term is preferably written in singular form where appropriate.

Overall qualifier cardinality. One or more qualifiers are required for each PhenoDE (1..n). Each individual qualifier category has cardinality 0..n unless a modality- or protocol-specific rule makes that category mandatory.

Ex vivo requirement. ST has cardinality 1..n for ex vivo records. An ex vivo record without ST is incomplete and requires further curation before finalization.

Repeatability. A qualifier category may contain multiple normalized values when all values apply simultaneously to the same measurement. Exact duplicates should not be repeated.

Mutual exclusivity. Qualifier categories are not globally mutually exclusive. Alternative or incompatible values should be represented in separate PhenoDEs unless the source explicitly defines a combined measurement.

Presentation order. List the feature first, followed by measurement-object qualifiers (ST, HAP, ORP, AS), measurement-process qualifiers (MM, MT, MC), measurement-result qualifiers (MID, SI, MU), and Others last. This order is organizational and does not imply logical priority.

Mapping-scope distinction. Structural cardinality and mapping eligibility are separate. In the current external mapping analysis, MT is retained in the PhenoDE representation but excluded from qualifier mapping. Accordingly, the label “Only qualifier MT” in the mapping tables denotes records for which MT was the sole qualifier. Because MT was excluded from external mapping, no qualifier mapping result was assigned; this label does not denote a PhenoDE with zero qualifiers.

5. Worked examples

Example 1 - retinal measurement (PhenoDE PDE057024763.1). PhenoDE Term: Left nerve fibre layer inner inferior thickness of macular. Feature class: in vivo measurement. Feature term: macular thickness. Qualifiers: HAP = eye; macula; retinal nerve fibre layer; ORP = left; inner; inferior; MT = optical coherence tomography for ophthalmology; MU = μm. The anatomical structures, orientation, tool, and unit refine the measurement, while the feature identifies the core measurable characteristic.

https://www.biosino.org/PhenoDE/browser/detail/PDE057024763.1

Example 2 - carotid ultrasonography (PhenoDE PDE078057999.1). PhenoDE Term: Intima-media thickness of left distal common carotid artery. Feature class: in vivo measurement. Feature term: intima-media thickness. Qualifiers: HAP = carotid artery; common carotid artery; ORP = left; distal; MT = Doppler Ultrasound Diagnostic System (Philips EPIQ7); MU = cm. The anatomical site and relative position are represented separately from the measurement tool and unit.

https://www.biosino.org/PhenoDE/browser/detail/PDE078057999.1

6. Essential boundary cases

HAP versus ORP. HAP identifies an anatomical structure, whereas ORP identifies laterality, direction, relative position, or spatial relationship. HAP and ORP do not overlap; directional or positional information should not be assigned to HAP.

MM versus MT versus MC. MM is the procedure or rule; MT is the instrument, device, software, or scale; MC is the timing, state, environment, or setting in which measurement occurs.

MC versus MID. MC describes measurement context; MID identifies a measured component, channel, item category, or result dimension.

MID versus SI. MID is not a statistical operation. Average, maximum, standard deviation, and percentile belong to SI.

AS versus MC. AS identifies the applicable population or subject attribute; MC describes the circumstances of measurement.

Others. Use only when a term cannot be assigned to any category defined in Table 1.
